# Crystal Engineering of a Chiral Crystalline Sponge That Enables Absolute Structure Determination and Enantiomeric Separation

**DOI:** 10.1021/acs.cgd.3c00446

**Published:** 2023-05-16

**Authors:** Chenghua Deng, Bai-Qiao Song, Matteo Lusi, Andrey A. Bezrukov, Molly M. Haskins, Mei-Yan Gao, Yun-Lei Peng, Jian-Gong Ma, Peng Cheng, Soumya Mukherjee, Michael J. Zaworotko

**Affiliations:** † Bernal Institute, Department of Chemical Sciences, University of Limerick, Limerick V94 T9PX, Ireland; ‡ Department of Chemistry and Key Laboratory of Advanced Energy Material Chemistry, College of Chemistry, Nankai University, Tianjin 300071, China

## Abstract

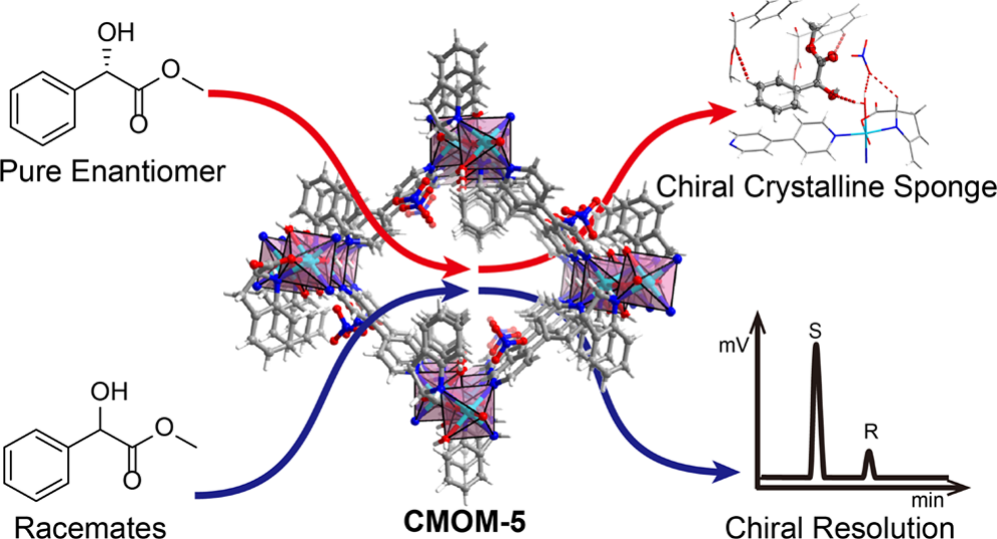

Chiral metal–organic materials (CMOMs), can offer molecular binding sites that mimic the enantioselectivity exhibited by biomolecules and are amenable to systematic fine-tuning of structure and properties. Herein, we report that the reaction of Ni(NO_3_)_2_, *S*-indoline-2-carboxylic acid (*S*-IDECH), and 4,4′-bipyridine (bipy) afforded a homochiral cationic diamondoid, **dia**, network, [Ni(*S*-IDEC)(bipy)(H_2_O)][NO_3_], **CMOM-5**. Composed of rod building blocks (RBBs) cross-linked by bipy linkers, the activated form of **CMOM-5** adapted its pore structure to bind four guest molecules, 1-phenyl-1-butanol (1P1B), 4-phenyl-2-butanol (4P2B), 1-(4-methoxyphenyl)ethanol (MPE), and methyl mandelate (MM), making it an example of a chiral crystalline sponge (CCS). Chiral resolution experiments revealed enantiomeric excess, *ee*, values of 36.2–93.5%. The structural adaptability of **CMOM-5** enabled eight enantiomer@**CMOM-5** crystal structures to be determined. The five ordered crystal structures revealed that host–guest hydrogen-bonding interactions are behind the observed enantioselectivity, three of which represent the first crystal structures determined of the ambient liquids *R*-4P2B, S-4P2B, and *R*-MPE.

## Abstract 2

A new chiral metal−organic material provides a blueprint for the design of families of chiral crystalline sponges that enable enantiomeric selectivity, and the determination of the crystal structures of liquid enantiomers.

## Introduction

Enantiomers of chiral molecules can behave very differently in biological systems, e.g., one enantiomer might drive a physiological function, while the other is toxic. This means that high enantiomeric purity can be a requirement in specialty chemicals such as pharmaceuticals, fragrances, condiments, and agrochemicals.^1−4^ Although natural products tend to be homochiral, this is not typically the case for synthetic compounds. In the context of pharmaceutical compounds, 167 small-molecule drug products were approved by the US Food and Drug Administration, USFDA, from 2018 to 2022 (Table S1). Of these, 101 are homochiral, whereas only nine are racemic. This can cause challenges for purification as the identical physical properties of enantiomers mean that separation of racemic mixtures into their enantiomerically pure components is difficult and costly, tending to rely upon enantiomeric separation^5−7^ and/or asymmetric synthesis.^8,9^

In the context of separations, the early promise of chiral cyclodextrin and polysaccharide derivatives as scaffolds for separating racemates through supramolecular chemistry has not been fully realized, with poor stability and high cost handicapping their commercial utility.^8,10^ Chiral porous materials have the potential to overcome these challenges if they exhibit the right pore size and chemistry to enable effective chiral separation performances. Further, they can afford insight into selective binding mechanisms.^11−15^

Single-crystal X-ray diffraction (SCXRD) studies can offer direct structural information with atomic-level precision. Although absolute structure determination of crystalline chiral compounds is feasible by SCXRD,^16,17^ it is not always possible to readily obtain suitable single crystals, especially when one is dealing with liquids or solid compounds (e.g., natural products or potential drug candidates) that are only available in small quantities.^18^ In this context, the introduction of “*crystalline sponges*” represents a seminal breakthrough that offers promise to address the limitations of SCXRD.^19−24^ This is because, in a typical crystalline sponge experiment, guest-accessible metal–organic materials (MOMs) can adsorb and orient their pores to accommodate organic molecules in an ordered manner, thereby enabling spatial precision.^25^ The prototypal crystalline sponges were metal–organic frameworks (MOFs),^20^ but other classes of compounds such as hydrogen-bonded organic frameworks (HOFs) and metal–macrocycle frameworks can also function as crystalline sponges.^26−28^

The prototypal crystalline sponge, the achiral MOF [(ZnI_2_)_3_(tpt)_2_*x*(solvent)]*_n_* (tpt = tris(4-pyridyl)-1,3,5-triazine), **ZnI**_**2**_**-tpt**, enabled structural determination of several chiral molecules.^29−33^ To observe the effective anomalous scattering from the host, **ZnI**_**2**_**-tpt** was preinstalled with a chiral reference, following which the absolute guest molecule configurations could be determined.^34^ Our group has developed chiral crystalline sponges (CCSs) based on homochiral MOMs (CMOMs) for the structure determination of chiral compounds, and chiral separation performances thereof.^35−37^

A general feature of MOMs, including MOFs, is that, because they are sustained by the coordination of linker ligands to metal nodes (or clusters), they are inherently amenable to design from first principles, i.e., crystal engineering.^38−41^ This is a desirable feature since it means that families (platforms) of related materials can be prepared^42−48^ and their functional properties can be studied systematically.^46,49,50^ An increasingly important subset of MOMs is chiral MOMs (CMOMs), which are composed of homochiral ligands or chiral channels that arise from the crystal packing of achiral components. Since the report of **POST-1** in 2000,^51^ it has been realized that CMOMs offer potential utility in asymmetric catalysis, chiral detection, and enantiomeric separations.^9,52−55^ However, the use of high-cost homochiral ligands, especially derivatives of binaphthyl and Schiff bases, is a hindrance to the development of CMOMs into higher technological readiness levels.^52,56,57^ Synthetic derivatives of amino acids (particularly glycine, alanine, and histidine) have also been used as ligands to build flexible CMOMs and further studied as crystalline sponges (e.g., **ZnGGH**)^58−60^ and enantiomeric separation materials (e.g. **TAMOF-1**).^60−62^

**Scheme 1:**
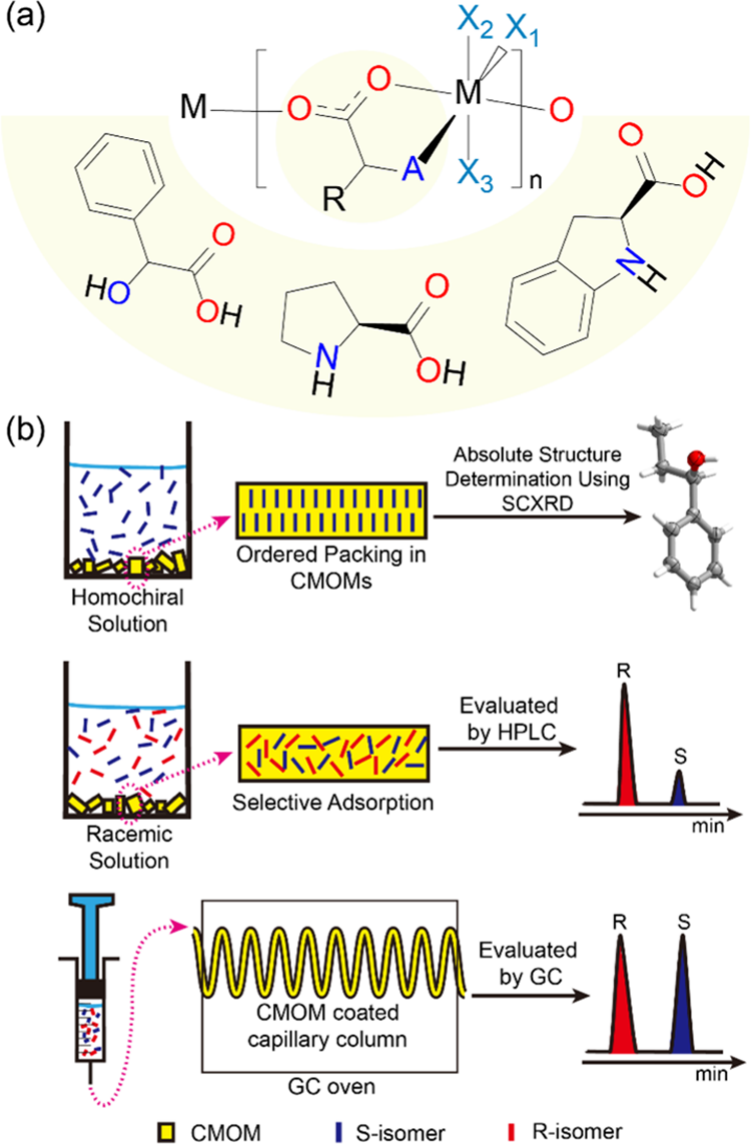
(a) Crystal Engineering of Octahedral Metal Cations with α-Hydroxy and Prolinato Ligands Can Afford Related Platforms of RBB-Based CMOMs; (b) CMOMs as CCS Materials (Top), Enantioselective Adsorbents (Middle), and Chiral Stationary Phases (Bottom)

In our group, we have targeted low-cost homochiral ligands such as mandelic acid, which forms a platform of CMOMs sustained by rod building blocks (RBBs).^35−37,63^ Mandelic acid, an α-hydroxy acid, can build RBBs through simultaneous chelation of a metal cation and bridging to an adjacent metal cation. Mandelate anions thereby occupy three coordination sites of each metal cation in an RBB, typically in a *mer*- configuration for an octahedral cation (Scheme 1a, left, A = OH, Figure S2 and Table S2).^35,64^ The remaining coordination sites of each metal cation are therefore available to be linked with N-donor linker ligands such as 4,4′-bipyridine, bipy, to form two-dimensional (2D) or three-dimensional (3D) coordination networks. In the case of mandelate CMOMs, Co^2+^ and Zn^2+^ RBBs were linked by 1.5 equivalents of bipy to afford 5-connected, 5-c, cationic **bnn** networks in which all octahedral coordination sites are occupied. These materials were found to function as adaptive CCSs, affording ordered chiral guest molecules, insight into host–guest binding, and determination of absolute configurations (Scheme 1b).^35−37^ They were also found to exhibit potential for enantio-separation by gas chromatography (GC).^36^

That the coordination geometry in Scheme 1a might also occur for A = NH is suggested by the existence of RBB structures for prolinato complexes of transition metals. Specifically, our CSD survey^65^ revealed 14 RBB-sustained structures (Figure S3 and Table S3). In one of these examples, **[Cd(l-prolinato)(bipy)(NO_3_)]**, in which the prolinato ligand coordination in a *fac*- manner, the RBB was linked by one equivalent of bipy to generate a square lattice, **sql**, topology coordination network.^66^ In this contribution, we report the first use of the chiral compound *S*-indoline-2-carboxylic acid, *S*-IDECH (Scheme 1a, right), to build an RBB-sustained coordination network in combination with one equivalent of bipy, [Ni(*S*-IDEC)(bipy)(H_2_O)][NO_3_], **CMOM-5**, and its CCS properties (Scheme 1b). We were attracted to *S*-IDECH as it is an abundant natural product found in *Strychnos cathayensis*.^67,68^ Our CSD search revealed that *S*-IDEC has not been previously used as a ligand. We selected several homochiral aromatic alcohols to evaluate the properties of CMOM-5. In general, such molecules are key precursors to enantiopure pharmaceuticals.^69−71^ The isomers of 1-phenyl-1-butanol (1P1B), 4-phenyl-2-butanol (4P2B), 1-(4-methoxyphenyl)ethanol (MPE), and methyl mandelate (MM) are key enantiopure reagents for the total synthesis of chiral pharmaceuticals or bioactive nature products, e.g., corticotropin-releasing factors, chiral arylamines, fluorohexestrol, and (−)-disorazole C1.^72−80^ Under ambient conditions, all six isomers of 1P1B, 4P2B, and MPE are liquids, and only one enantiomer, *S*-1P1B, has had its structure crystallographically determined, in ***S*****-1P1B@CMOM-3S** (Table S4).^36^

## Experimental Section

All reagents and solvents were obtained from commercial vendors and used without further purification. More details of the experimental procedures are described in the Supporting Information (SI).

### Characterization

SCXRD data were collected at 150 K using a Bruker D8 Quest diffractometer equipped with a Cu Kα IμS micro-focus source (λ = 1.54178 Å) and Photon II detector. Temperature was controlled by an Oxford Cryosystem with liquid nitrogen flow. In all cases, the data was indexed by APEX4 (v2021.10–0). Integrations were conducted by SAINT V8.40A in APEX4. Absorption corrections were performed by SADABS in APEX4. Space group determination was performed by XPREP in APEX4. Structures were solved using the Olex2–1.5 software package and SHELXT through Intrinsic Phasing. Refinement was conducted by SHELXL through full-matrix least-squares on *F*^2^.^81−83^ Electron density corresponding to highly disordered guest molecules was addressed by PLATON SQUEEZE.^84,85^ Occupancy of chiral guest molecules was determined by considering the MASK-calculated electrons and the ratios among the guests and the ligands reflected in the corresponding ^1^H nuclear magnetic resonance (NMR) spectra.^25,26,86^

### Synthesis

An ethanolic solution of Ni(NO_3_)_2_·6H_2_O and *S*-indoline-2-carboxylic acid (also known as l-indoline-2-carboxylic acid) was mixed with an *N*,*N*-dimethylformamide (DMF) solution of bipy in a 15 mL glass vial. The reaction mixture was heated at 60 °C for 24 h. Blue single crystals of **CMOM-5** were obtained upon cooling to RT. Single crystals of **CMOM-5** were soaked in acetonitrile (MeCN) for 5 days, with fresh solvent exchanged daily, before proceeding with crystalline sponge and chiral resolution experiments.

### Chiral Resolution

90 mg of MeCN-exchanged **CMOM-5** crystals was soaked in 0.5 mL of MeCN containing 400 mg (or μL) of the racemates. The screw cap was loosened to enable slow evaporation of MeCN over 5 days. Crystals were then filtered and washed with ethyl acetate and then *n*-hexane to remove the residual chiral molecules on the surface of the crystals. Guest molecules in **CMOM-5** were extracted by soaking the crystals in 10 mL of methanol (MeOH) for 3 days, following which the crystals were filtered and washed with MeOH. The filtrates were combined, and the solvent was removed by a rotary evaporator. The dried fractions were dissolved in 1 mL of MeOH for *ee* analysis.

### CCS Experiment

MeCN-exchanged **CMOM-5** crystals were soaked in 0.5 mL of MeCN containing 40 mg of isomers of MM, or 40 μL of isomers of 1P1B, 4P2B, and MPE. The screw cap was loosened to enable slow evaporation of MeCN over 3 days; single crystals were isolated for SCXRD experiments; see Figure S1.

## Results and Discussion

**Figure 1:**
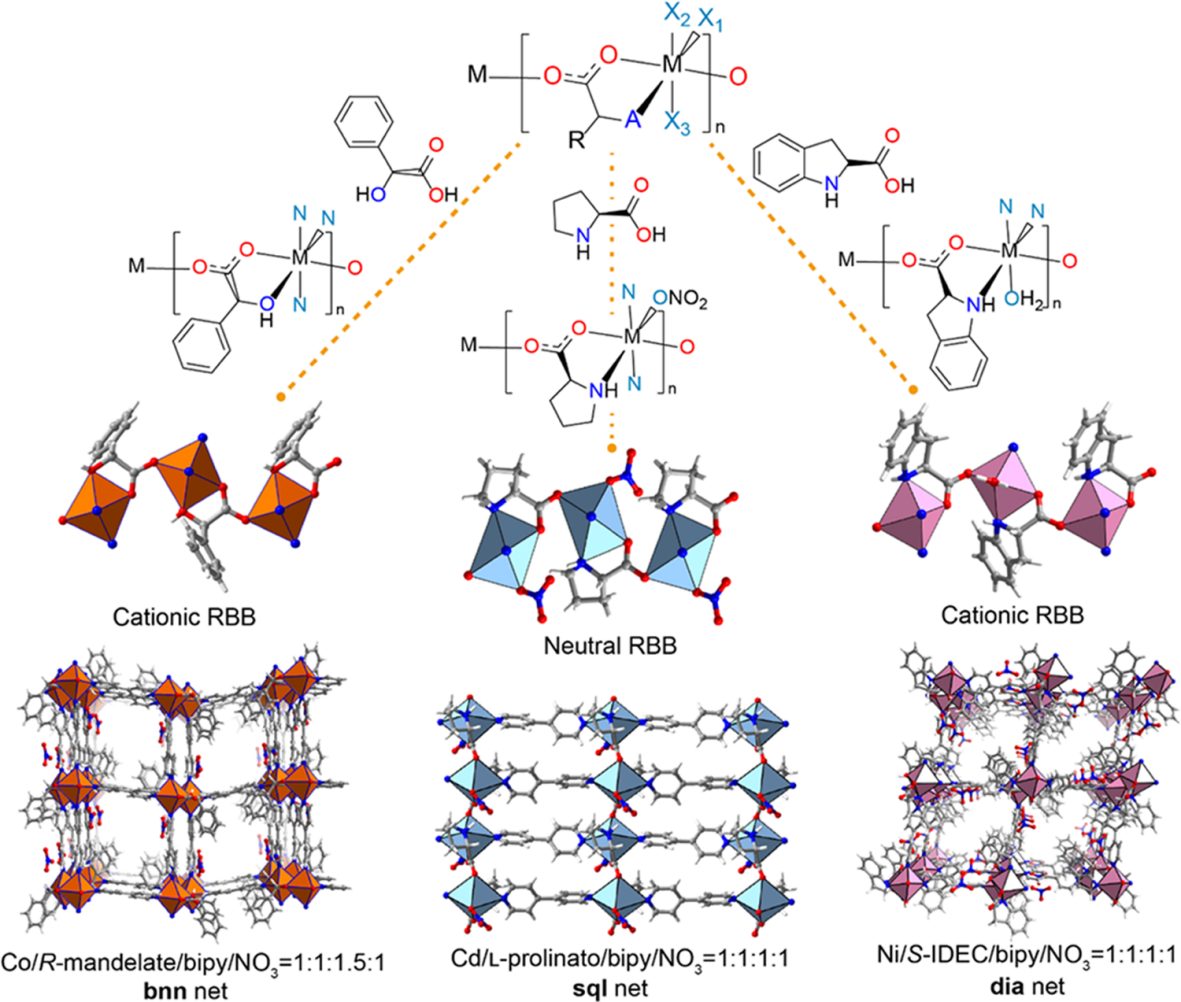
**Top**: Schematic of the repeating unit of an RBB based upon an octahedral metal cation; “A” represents O or N and X_1_–X_3_ are the coordination sites occupied by atoms from linkers or terminal ligands. **Bottom**: Crystal engineering of RBB-based coordination networks can afford CMOMs sharing a common RBB (top): **CMOM-1R** (left), **[Cd(l-prolinato)(bipy)(NO_3_)]** (middle), and **CMOM-5** (right).

The coordination geometry of RBB nodes sustained by mandelate or prolinate anions and octahedral metal cations is generalized in Scheme 1a. For mandelate CMOMs, three coordination sites are occupied by oxygen atoms of chelating and bridging mandelate anions, and three by nitrogen atoms of bipy linkers (X_1_, X_2_, X_3_ = N, A = O, Scheme 1a and Figure 1, left). As mentioned above, the resulting cationic coordination network can be regarded as a 5-*c***bnn** topology net.^35^ With respect to the 14 RBB structures involving prolinato ligands, the coordination geometries were found to be octahedral (6-coordinated, 4 examples) or pyramidal (5-coordinated, 10 examples). Thirteen one-dimensional RBB structures are comprised of Cu^2+^ or Zn^2+^ central cations that are also coordinated to terminal ligands besides prolinato. Perhaps the most relevant example is the coordination network **[Cd(l-prolinato)(bipy)(NO_3_)]** in which the prolinato ligand coordinates as a *mer*- isomer, whereas the pyridyl moieties occupy *trans*- positions (sites X_2_, X_3_).^66^ The nitrate counter anion occupies the final coordination site as a nitrato ligand (site X_1_). The resulting coordination network is based upon 4-c nodes, while the linearity of the bipy ligands affords **sql** topology (Figure 1, middle).

**CMOM-5** crystallized in the orthorhombic space group *P2*_1_2_1_2_1_ (Table S5) and the crystal structure revealed that Ni(II) cations coordinate to S-IDEC to form one-dimensional (1D) cationic RBBs in a manner similar to that of the prolinato RBBs discussed above with prolinato ligands adopting mer- geometry. The pyridyl moieties of **CMOM-5** exhibit *cis*-geometry (sites X1, X3), which has important consequences as **sql** topology is in effect precluded. Rather, a different 4-connected topology, diamondoid, **dia**, is exhibited by **CMOM-5** (Figures 1(right) and S6). The final coordination site is occupied by an aqua ligand (site X_2_), which forms hydrogen bonds (H-bonds) with extra-framework nitrate counterions (Figure S12 and Table S13). The channels represent *ca*. 41.3% of the void volume of the unit cell from PLATON,^84^ which were found to be also occupied by solvent molecules. Thermogravimetric analysis (TGA), revealed 17% weight loss by 156 °C corresponding to the loss of DMF molecules, and thermal decomposition starting at ca. 250 °C (Figure S26). Bond distances and angles are within expected ranges (Tables S12).

**Figure 2:**
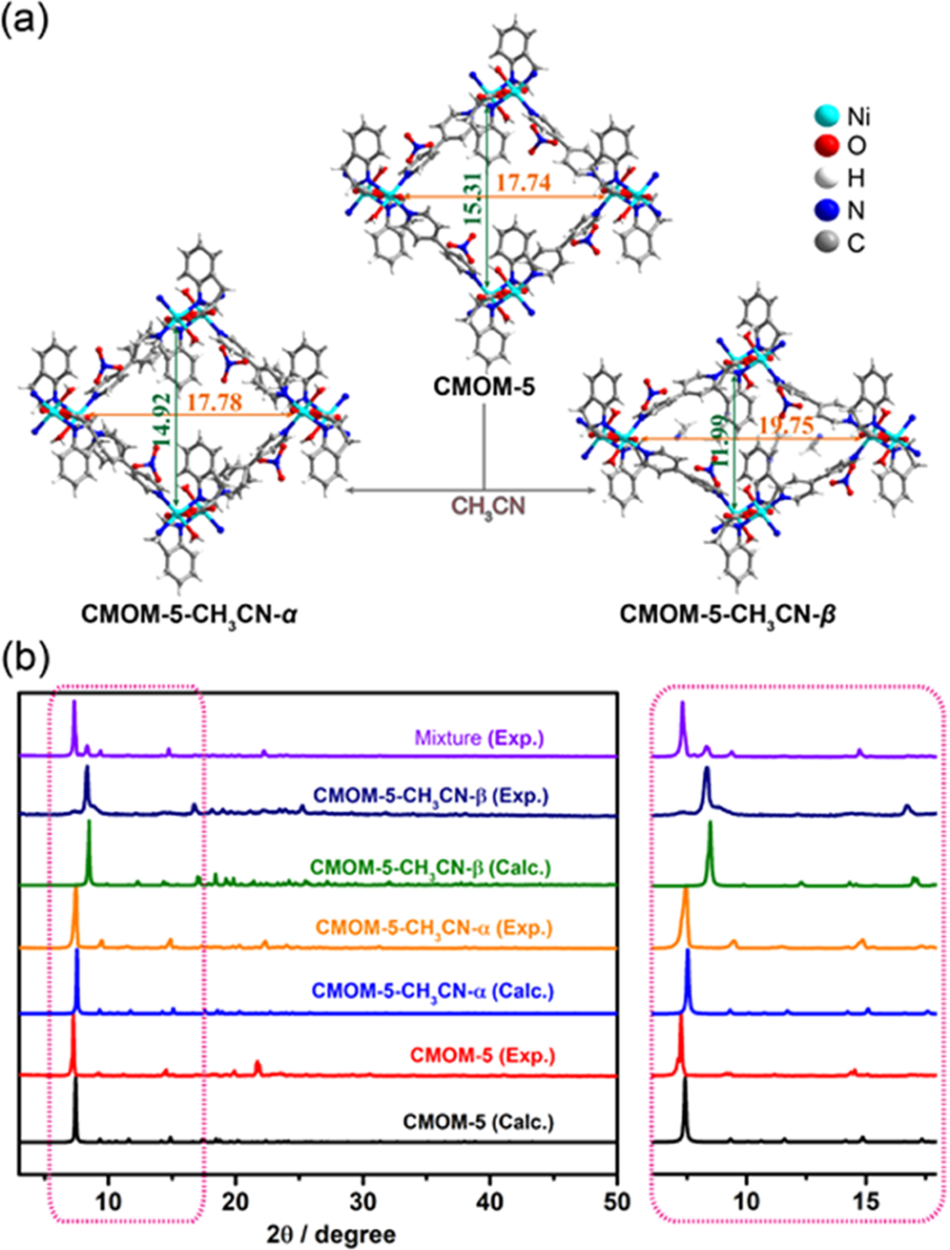
Structural differences between **CMOM-5**, **CMOM-5-CH**_**3**_**CN-α** and **CMOM-5-CH**_**3**_**CN-β**. (a) Structures of **CMOM-5**, **CMOM-5-CH**_**3**_**CN-α**, and **CMOM-5-CH**_**3**_**CN-β** viewed along the channels (distances in Å). (b) PXRD patterns of **CMOM-5**, **CMOM-5-CH**_**3**_**CN-α**, **CMOM-5-CH**_**3**_**CN-β**, and the mixture of **CMOM-5-CH**_**3**_**CN-α** and **CMOM-5-CH**_**3**_**CN-β**; the zoomed region on the right is magnified for clarity.

Crystals of **CMOM-5** were soaked in MeCN to remove DMF solvent molecules prior to further studies. After solvent exchange, **CMOM-5** was found to transform into two phases: **CMOM-5-CH**_**3**_**CN-α** and **CMOM-5-CH**_**3**_**CN-β**. Both phases exhibited the same space group and coordination connectivity as the as-synthesized phase (Table S6). The structural differences between **CMOM-5**, **CMOM-5-CH3CN-α**, and **CMOM-5-CH3CN-β** are reflected in the distances between the nickel atoms along the opposite ends of the quadrangular channel (Figure 2a). Powder X-ray diffraction (PXRD) patterns reflected the phase change from the as-synthesized crystals to the MeCN-exchanged crystals (Figure 2b). The weight loss of MeCN in **CMOM-5-CH**_**3**_**CN-α** and **CMOM-5-CH**_**3**_**CN-β** below 81 °C (the boiling point of MeCN) was about 17 and 14 wt %, respectively. That more MeCN was lost in **CMOM-5-CH**_**3**_**CN-α** than **CMOM-5-CH**_**3**_**CN-β** is consistent with the relative unit cell volumes. In **CMOM-5-CH3CN-β**, a pyridine ring of bipy was found to be 2-fold disordered with occupancies of 68.9 and 31.1%. The dihedral angles between the pyridine rings in the disordered bipy are shown in Figure S11b,c. The nitrate anion formed H-bonds with the aqua ligand (Figure S13 and Table S14). CH_3_CN molecules were found disordered in the channel, leaving *ca*. 33.7% void volume of the unit cell according to PLATON SQUEEZE data.^84^ In **CMOM-5-CH**_**3**_**CN-β**, two MeCN molecules were refined anisotropically in the asymmetric unit. MeCN molecules interacted with the host framework through H-bonds and C–H···π interactions (Figure S14 and Table S15).^87^

After soaking the MeCN-exchanged crystals in *n*-hexane, a new phase, **CMOM-5-Hex**, was obtained (Table S7), in which *n*-hexane was present in the asymmetric unit. Hexane molecules were found to engage in C–H···π interactions (Figure S16 and Table S17).^87^ Upon soaking the MeCN-exchanged crystals in isopropanol (IPA)/hexane (5:95), the crystals transformed into **CMOM-5-IPA_Hex** (Table S7). An IPA molecule was crystallographically identified in **CMOM-5-IPA_Hex**, interacting with aqua ligands through O–H···O H-bonds (Figure S17 and Table S18). The experimental PXRD patterns of **CMOM-5-Hex** and **CMOM-5-IPA_Hex** confirmed bulk phase purity and indicated that both **CMOM-5-CH3CN-α** and **CMOM-CH3CN-β** transform into the same phase after solvent exchange (Figures S28 and S29). That solvent molecules were refined despite structural changes suggests the potential to serve as a self-adaptive crystalline sponge and identification of chiral molecules.

**Figure 3:**
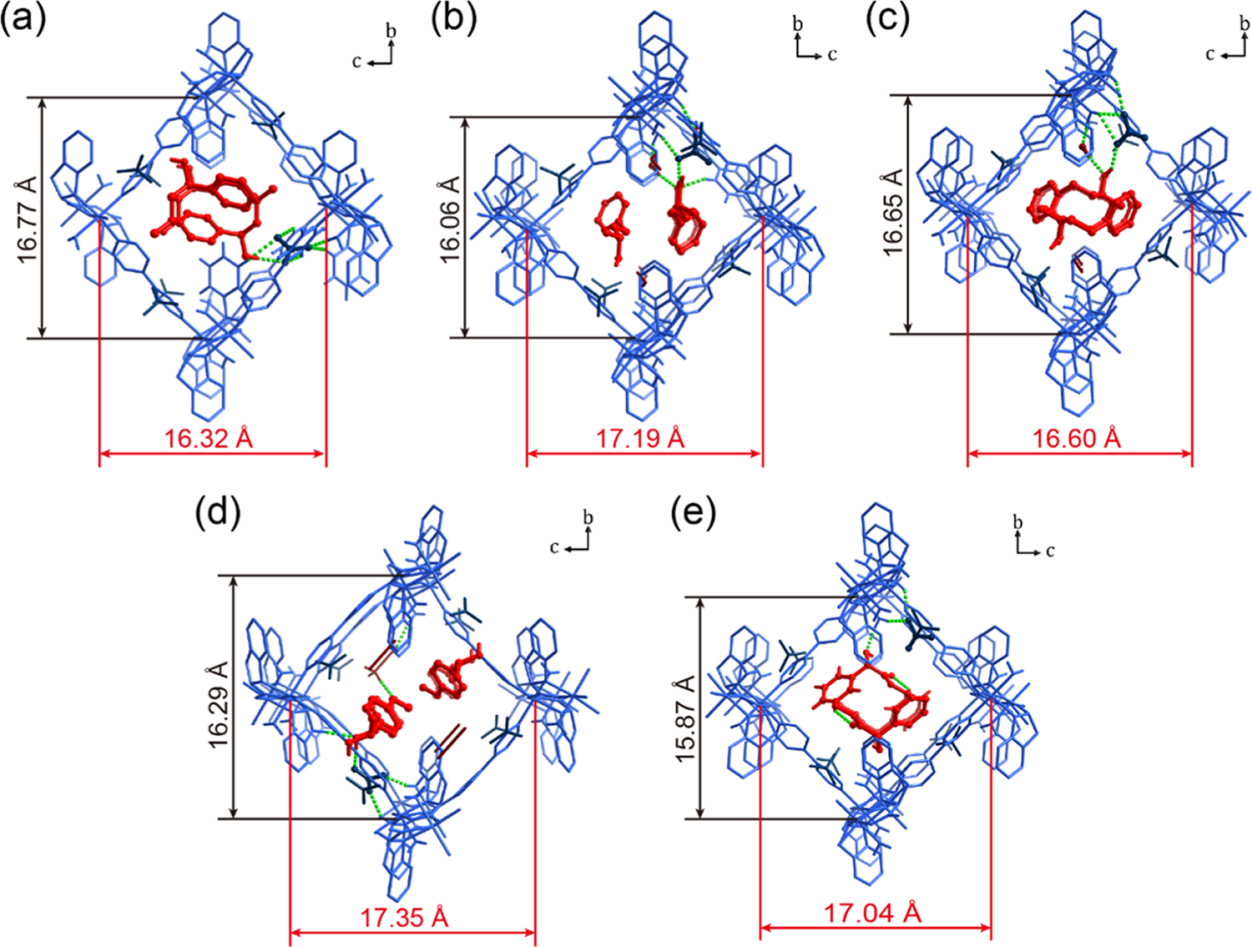
Binding sites of the chiral guests in the channels of **CMOM-5**. (a–e) **CMOM-5-*****R*****-1P1B**, **CMOM-5-*****R*****-4P2B**, **CMOM-5-*****S*****-4P2B, CMOM-5-*****R*****-MPE**, and **CMOM-5-*****S*****-MM**, respectively. The host frameworks are labeled in blue, nitrate anions are labeled in dark teal, chiral guests are labeled in red, and solvent molecules are labeled in brown. Hydrogen atoms on the C–H bonds and N–H bonds were selectively omitted for clarity. Short contact interactions between H atom and acceptor moiety of H-bonds (between the guests and the framework) are shown by red dashed lines.

CCS experiments were conducted on the isomers of 1P1B, 4P2B, MPE, and MM. Each structure crystallized in the *P2*_1_2_1_2_1_ space group (Tables S8–S11). As noted above, *S*-1P1B was studied via the crystalline sponge method^36^ but no structures have been reported for the other molecules (Figures S4 and S5 and Table S4). The PXRD patterns reflect that each chiral isomer had induced **CMOM-5** to transform to a chiral guest-loaded phase (Figures S30–S33). In **CMOM-5-*****R*****-1P1B**, **CMOM-5-*****R*****-4P2B**, **CMOM-5-*****S*****-4P2B**, **CMOM-5-*****R*****-MPE**, and **CMOM-5-*****S*****-MM**, the chiral guests were observed by SCXRD, the nonhydrogen atoms on the chiral molecules being refined anisotropically (Figures S21–S25). Each asymmetric unit contains one chiral guest molecule. The positions of chiral guests, the nitrate anion, and solvent molecules in the channels are illustrated in Figure 3. **CMOM-5** was found to adapt to chiral molecules by changing the shape of its framework (Figures 3 and S9) and the position of the nitrate anion (Figures 3 and S18–S25). Indeed, each homochiral guest induced structural transformations in various ways as indicated by the differences between unit cell parameters and volumes (Figures S7 and S8), and configurations of the coordinated ligands (bipy and S-IDEC) (Figures S10 and S11). Compared to the two MeCN-loaded phases, **α** and **β**, all of the chiral guest-loaded structures exhibited larger unit cell volumes (Figure S8).

**Figure 4:**
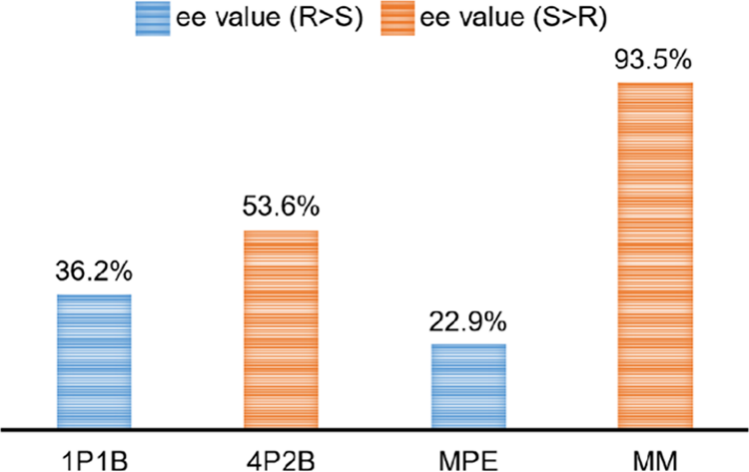
Chiral resolution studies involving **CMOM-5** revealed *ee* values determined by HPLC using chiral columns as the chiral stationary phase and a UV detector.

Chiral resolution experiments were conducted to study the chiral discrimination properties of **CMOM-5** as an enantioselective material after being exchanged with MeCN for 5 days. The uptake of the chiral molecules was determined by ^1^H NMR spectra of the solids digested in a mixture of deuterium chloride and deuterated dimethyl sulfoxide (Figures S42–S45). The ratio of the chiral guest, *S*-IDEC, and bipy was observed to be *ca*. 1:1:1, consistent with one chiral molecule per cavity. The enantioselective adsorption performance was evaluated by *ee* values (Figure 4) calculated from high-performance liquid chromatography (HPLC) of the solution-extracted loaded samples of **CMOM-5** (Figures S46–S61). **CMOM-5** exhibited preference for the *R*- isomers of 1P1B and MPE with *ee* values of 36.2 and 22.9%, respectively. **CMOM-5** was selective for the *S*-isomers of 4P2B and MM with *ee* values are 53.6 and 93.5%, respectively.

After conducting chiral resolution experiments, MeOH-washed crystals were found to be stable under ambient conditions, and the SCXRD crystal structure was revealed to be **CMOM-5-MeOH**. **CMOM-5-MeOH** retained the same space group and coordination connectivity as the as-synthesized structure (Tables S5 and S16 and Figure S15). The PXRD pattern of **CMOM-5-MeOH** was consistent with the bulk sample preserving both crystallinity and phase purity (Figure S27).

To the best of our knowledge, there are no previous reports of chiral resolution experiments involving 1P1B.^11,12^ Kinetic resolution studies in the presence of catalysts that selectively transformed one isomer of the racemate have been reported.^88^ For MPE and 4P2B, **CMOM-5** exhibited lower *ee* values than **(Δ**_**12**_**)-PCC-57**, a chiral metal–organic cage (MOC), which preferred *S*-4P2B and *S*-MPE with 99.9% *ee* (Table S27).^89^ For **CMOM-5** and MM, the *ee* compares to **[DyNaL(H**_**2**_**O)**_**4**_**] 6H**_**2**_**O** (H_4_L = 3,3′-di-*tert*-butyl-5,5′-di(3,5-carboxyphenyl-1-yl)-6,6-dimethylbiphenyl-2,2-diol), which preferred *S*-MM with 93.1% *ee* (Table S27).^90^ Relative to **CMOM-5**, **(Δ**_**12**_**)-PCC-57** and **[DyNaL(H**_**2**_**O)**_**4**_**] 6H**_**2**_**O** require more complex synthesis conditions for their preparation.

**Figure 5:**
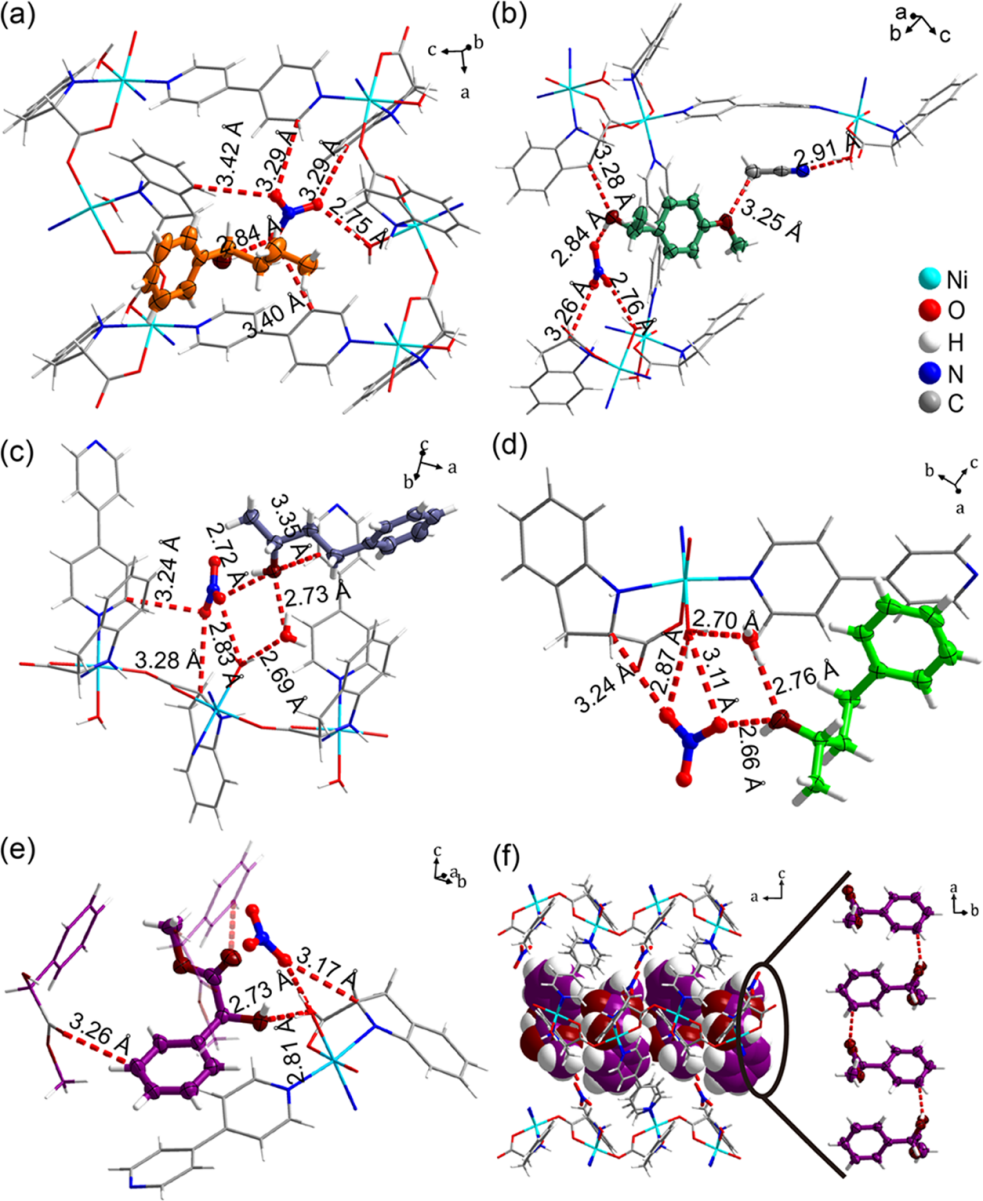
H-bond interactions around the chiral guests in **CMOM-5-*****R*****-1P1B** (a); **CMOM-5-*****R*****-MPE** (b); **CMOM-5-*****R*****-4P2B** (c); **CMOM-*****S*****-4P2B** (d); **CMOM-5-S-MM** (e). H-bond sustained chain of *S*-MM along the **CMOM-5-*****S*****-MM** channel (f). For (a–f), the nonhydrogen atoms of the refined guest molecules were drawn in thermal ellipsoids at 50% probability. H-bond interactions between the guest molecules were labeled by red dashed lines.

We next analyzed the guest-loaded crystal structures to gain insight into the observed chiral resolution performance. The hydroxy group of *R*-1P1B was found to interact with a nitrate anion through O–H···O hydrogen bonding (2.84 Å, Figure 5a and Table S19). *R*-1P1B also formed host–guest and guest–guest C–H···π interactions (Figure S21). In **CMOM-5-*****S*****-1P1B**, guest molecules were disordered in the channel (Figure S18 and Table S20).^84^ That the unit cell of **CMOM-5-*****R*****-1P1B** is different than **CMOM-5-*****S*****-1P1B**, *a* and *b* longer by 0.20 and 1.03 Å, respectively, and *c* shorter by 1.13 Å (Table S8), reveals the adaptive nature of **CMOM-5**. ^1^H NMR spectra of digested **CMOM-5-*****S*****-1P1B** crystals revealed a 1:1.01:1.15 ratio of bipy/*S*-IDEC/1P1B, consistent with the crystal structure (Figure S34) and surface bound *R*-1P1B. A 1:1.01:0.5 ratio was found for **CMOM-5-*****S*****-1P1B** (Figure S35). The higher loading of *R* isomer loaded could be ascribed to guest–guest C–H···π interactions.^91−93^

In **CMOM-5-*****R*****-MPE**, the methylene C–H moiety from *S*-IDEC interacts with the hydroxy group of *R-*MPE through C–H···O H-bonds (3.28 Å), and the hydroxy group interacts with a nitrate anion through O–H···O H-bonds (2.84 Å). The oxygen atom in the ether group of *R*-MPE exhibits guest–guest C–H···O H-bonds (3.25 Å) and MeCN molecules reside in channels (Figure 5b and Table S23). Bipy and *S*-IDEC ligands of the host framework exhibit C–H···π interactions with the chiral guest *R*-MPE. Further, there are guest–guest C–H···π interactions between *R*-MPE molecules (2.99 and 3.69 Å, Figure S24). For the structures with ordered chiral guests and solvent molecules, **CMOM-5-*****R*****-MPE** exhibited the largest unit cell volume (Figure S8). The *a*, *b*, and *c* axes in **CMOM-5-*****S*****-MPE** are 0.07, 0.15, and 0.41 Å, respectively, less than those of **CMOM-5-*****R*****-MPE** (Table S10). Additionally, unlike the other chiral guest-loaded structures, the dihedral angle formed by the two pyridine rings in **CMOM-5-*****R*****-MPE** orients in the opposite direction (Figure S11). In **CMOM-5-*****S*****-MPE**, the 42.1% void volume of the unit cell (calculated by PLATON SQUEEZE) is occupied by *S*-MPE and MeCN molecules.^83^ The nitrate anion interacts with the aqua ligand through O–H···O H-bonding (Figure S19 and Table S24). ^1^H NMR data revealed the ratio of bipy/*S*-IDEC/MPE in **CMOM-5-*****R*****-MPE** and **CMOM-5-*****S*****-MPE** to be 1:0.99:0.96 and 1:1.01:0.5, respectively (Figures S38 and S39). The higher uptake of *R* isomer and the guest–guest interactions could explain the higher ratio of *R* isomer from the chiral resolution experiments.^91−93^

In **CMOM-5-*****R*****-4P2B** and **CMOM-*****S*****-4P2B**, *R*-4P2B and *S*-4P2B were bound in a similar manner. The hydroxy groups of *R*-4P2B and *S*-4P2B interact nitrate anions through O–H···O H-bonds of 2.72 and 2.66 Å, respectively, while the channel water molecules interact with the hydroxy groups of *R*-4P2B and *S*-4P2B through O–H···O H-bonds of 2.73 and 2.76 Å, respectively (Figure 5c,d and Tables S21 and S22). In **CMOM-5-*****R*****-4P2B**, bipy ligands interact with *R*-4P2B through C–H···O H-bonds of 3.35 Å (Figure 5c and Table S21). The occupancy of both isomers was 100% in the corresponding structures, as supported by ^1^H NMR data revealing that the bipy/*S*-IDEC/4P2B ratio is 1:0.98:1.07 and 1:0.99:1.15 in **CMOM-5-*****R*****-4P2B** and **CMOM-5-*****S*****-4P2B**, respectively (Figures S36 and S37). Compared to **CMOM-*****R*****-4P2B**, the unit cell parameters of **CMOM-5-*****S*****-4P2B** are reduced by 0.13 and 0.65 Å along *a* and *c*, respectively, whereas *b* is increased by 0.6 Å (Table S9), resulting in a reduced unit cell volume for **CMOM-5-*****S*****-4P2B**. C–H···π interactions were found between *R*-4P2B molecules (3.65 Å, Figure S22). C–H···π interactions between *S*-4P2B molecules of 3.73 and 3.45 Å were observed (Figure S23). Stronger guest–guest C–H···π interactions in **CMOM-5-*****S*****-4P2B** could explain the higher loading of *S*-4P2B in the chiral resolution experiments.^91−93^

In **CMOM-5-*****R*****-MM**, the chiral guest molecules and solvent molecules were found to be disordered in channels (Figure S20 and Table S25). ^1^H NMR data revealed the ratio of bipy/*S*-IDEC/MM in **CMOM-5-*****R*****-MM** to be 1:1.03:0.52 (Figure S40), while in **CMOM-5-*****S*****-MM**, the ratio was 1:1:1.01 (Figure S41). The unit cell volume of **CMOM-5-*****S*****-MM** was determined to be smaller than the other chiral guest-loaded structures (Figure S8). Compared to **CMOM-5-*****R*****-MM**, *a* and *c* in **CMOM-5-*****S*****-MM** were found 0.12 Å and 0.44 Å shorter, respectively, while *b* increased by 0.12 Å. (Table S11). *S*-MM was bound to the host framework through O–H···O H-bonds from the hydroxy moiety to the aqua ligand (2.81 Å) and C–H···π interactions with bipy and *S*-IEDC (Figure S25 and Table S26). Intermolecular C–H···O H-bonds between the chiral guest molecules were observed (3.26 Å, Figure 5e). In the channel, guest–guest H-bonds meant that *S*-MM molecules formed infinite chains (Figure 5f). That **CMOM-5-*****S*****-MM** exhibited guest–guest H-bonding could be behind the strong separation performance.^93−95^

## Conclusions

In this work, the low-cost natural product *S*-IDECH is introduced as a homochiral ligand for MOMs, affording **CMOM-5**. Thanks to the adaptive properties of **CMOM-5**, it functions as a CCS for several solvents and chiral guests, enabling us to observe ordered enantiomers in five crystal structures for five of the eight guests studied, i.e.*, R*-1P1B, *R*-4P2B, *S*-4P2B, *R*-MPE, and *S*-MM. This report also represents the first time that the crystal structures of *R*-1P1B, *R*-4P2B, *S*-4P2B, and *R*-MPE have been determined. Overall, our study shows that a crystal engineering approach to the development of families of CMOMs from naturally abundant homochiral ligands could overcome the high cost^54,55^ of alternative approaches.

## Supporting Information Available

Experimental details; characterization results; chiral resolution experiment; data analysis; and computational calculations (PDF)

## Abbreviations

CMOMs: chiral metal–organic materials
*S*-IDEC: *S*-indoline-2-carboxylicate
bipy: 4,4′-bipyridine
RBB: rod building block
1P1B: 1-phenyl-1-butanol
4P2B: 4-phenyl-2-butanol
MPE: 1-(4-methoxyphenyl)ethanol
MM: methyl mandelate
CCS: chiral crystalline sponge
USFDA: US Food and Drug Administration
SCXRD: single-crystal X-ray diffraction
MOMs: metal–organic materials
MOFs: metal–organic frameworks
tpt: tris(4-pyridyl)-1,3,5-triazine
HOFs: hydrogen-bonded organic frameworks
GC: gas chromatography
NMR: nuclear magnetic resonance
DMF: *N*,*N*-dimethylformamide
dia: diamondoid
TGA: thermogravimetric analyses
PXRD: powder X-ray diffraction
IPA: isopropanol
CSD: Cambridge Structural Database
CSP: chiral stationary phase
*ee*: enantiomeric excess
HPLC: high-performance liquid chromatography
RT: room temperature
