# Supplementary material for: Crystal Engineering of a Chiral Crystalline Sponge That Enables Absolute Structure Determination and Enantiomeric Separation
Source: Cryst Growth Des. 2023 May 16;23(7):5211–20. doi: 10.1021/acs.cgd.3c00446 (PMC10326857; doi:10.1021/acs.cgd.3c00446)
Supplement: Supplementary file 1 — cg3c00446_si_001.pdf [file cg3c00446_si_001.pdf]

Crystal Engineering of a Chiral Crystalline Sponge That Enables Absolute Structure Determination and Enantiomeric Separation

Chenghua Deng,† Bai-Qiao Song,† Matteo Lusi,† Andrey A. Bezrukov,† Molly M. Haskins,† Mei-Yan Gao,† Yun-Lei Peng,† Jian-Gong Ma,‡ Peng Cheng,‡ Soumya Mukherjee,*,† Michael J. Zaworotko*,†

† Bernal Institute, Department of Chemical Sciences, University of Limerick, Limerick V94 T9PX, Ireland. Corresponding authors' emails: soumya.mukherjee@ul.ie; xtal@ul.ie

‡ Department of Chemistry and Key Laboratory of Advanced Energy Material Chemistry, College of Chemistry, Nankai University; Tianjin, 300071, China.

Table of Content

Materials 1
Synthesis and Solvent Exchange 1
Crystalline Sponge Experiment 2
Chiral Resolution Experiment 2
Analysis of USFDA-approved Drugs 3
CSD Data mining 3
Crystallographic Analysis Methods and Tables 14
Structural Details 23
Thermogravimetric Analysis 40
Powder X-ray Diffusion Analysis 41
NMR Spectra 45
HPLC Methods for the Analysis of Chiral Resolution Experiment Samples 57
References 66

Materials

Nickel (II) nitrate hexahydrate (Ni 79.8% min) was purchased from Alfa Aesar. 1-phenyl-1-butanol (1P1B, > 98.0%), 4-phenyl-2-butanol (4P2B, > 99.0%), 4,4'-bipyridine (bipy, > 98.0%). *R*-methyl-mandelate (*R*-MM, ≥ 98.0%) and *S*-methyl-mandelate (*S*-MM, ≥ 98.0%) were purchased from TCI. Methyl mandelate (MM, 95%), 1-(4-methoxyphenyl)ethanol (MPE, 97.0%), *S*-indoline-2-carboxylic acid (*S*-IDECH, 97.0%), *R*-1-(4-methoxyphenyl)ethanol (*R*-MPE, 95.0%) and *S*-1-(4-Methoxyphenyl)ethanol (*S*-MPE, 95.0%) were purchased from Fluorochem. Deuterium chloride solution (DCl, 35 wt. % in D_2O, ≥ 99 atom % D), d_6-dimethyl sulfoxide (d_6-DMSO, 99.9 atom % D), *R*-1-phenyl-1-butanol (*R*-1P1B, 97%), *S*-1-phenyl-1-butanol (*S*-1P1B, 97%), *R*-4-phenyl-2-butanol (*R*-4P2B, 98%) and *S*-4-phenyl-2-butanol (*S*-4P2B, 97%) were purchased from Sigma-Aldrich. *N,N*-dimethylformamide (DMF, for HPLC, ≥ 99.9%), ethanol (for HPLC, ≥ 99.8%), acetonitrile (for HPLC, ≥ 99.9%), methanol (for HPLC, ≥ 99.9%), ethyl acetate (for HPLC, ≥ 99.7%), n-hexane (for HPLC, ≥ 97.0%) and isopropanol (IPA, for HPLC, ≥ 99.0%) were purchased from Honeywell. All of them were used directly without further purification.

Synthesis and Solvent Exchange

(1) Synthesis of $\{[Ni(S\text{-IDEC})(bipy)(OH_2)](NO_3)(EtOH)(DMF)_{1.25}\}_n$ (**CMOM-5**)

$Ni(NO_3)_2{\cdot}6(H_2O)$ (87 mg, 0.3 mmol) and *S*-IDECH (50 mg, 0.3 mmol) were dissolved in 3 mL ethanol, and 4,4'-bipyridine (bipy, 0.3 mmol, 47 mg) was dissolved in 3 mL DMF. The two solutions were mixed in a vial, enclosed the cap, then move to the 60 °C oven for 24 hours. Needle shaped blue crystals were obtained, dried crystals were about 112 mg (0.25 mmol), about 81.68% yield based on Ni.

(2) Preparing of $\{[Ni(S\text{-IDEC})(bipy)(OH_2)](NO_3)(CH_3CN)_3\}_n$ (**CMOM-5-CH$_3$CN-*α***) and $\{[Ni(l\text{-IDEC})(bipy)(OH_2)](NO_3)(CH_3CN)_2\}_n$ (**CMOM-5-CH$_3$CN-*β***)

Crystals of as-synthesized **CMOM-5** were soaked in 10 mL acetonitrile for five days and the solvent were replaced by the flesh every day to completely remove DMF. **CMOM-5-CH$_3$CN-*α*** and **CMOM-5-CH$_3$CN-*β*** would obtain randomly.

(3) Preparing of $\{[Ni(S\text{-IDEC})(bipy)(OH_2)](NO_3)(n\text{-hexane})\}_n$ (**CMOM-5-Hex**)

Crystals of acetonitrile exchanged **CMOM-5** were soaked in 6 mL n-hexane for 5 days. The acetonitrile would be exchanged by n-hexane, then **CMOM-5-Hex** would be obtained.

(4) Preparing of $\{[Ni(S\text{-IDEC})(bipy)(OH_2)](NO_3)(IPA)(n\text{-hexane})_{0.5}\}_n$ (**CMOM-5-IPA_Hex**)

Crystals of acetonitrile exchanged **CMOM-5** were soaked in 3 mL n-hexane: IPA = 95:5 mixed solvent for 3 days. The acetonitrile would be exchanged by n-hexane and IPA and **CMOM-5-IPA_Hex** would be obtained.

(5) Preparing of $\{[Ni(S\text{-IDEC})(bipy)(OH_2)](NO_3)(MeOH)_4\}_n$ (**CMOM-5-MeOH**)

Soaking the crystals of **CMOM-5** after chiral resolution experiment in methanol for 3 days with the fresh solvent exchange every day. The SCXRD data was collected on the sample after 4P2B chiral resolution experiment.

Crystalline Sponge Experiment

Preparing of **CMOM-5-*R*-1P1B**: A few single crystals of acetonitrile exchanged **CMOM-5** were soaked in 0.5 mL acetonitrile solution which contains 40 mg *R*-1P1B. The screw cap was loosened to enable slow evaporation of acetonitrile for 3 days.[1]

Preparing of **CMOM-5-*S*-1P1B**: the same steps as **CMOM-5-*R*-1P1B** with the replacement of *R*-1P1B by *S*-1P1B.

Preparing of **CMOM-5-*R*-4P2B**: the same steps as **CMOM-5-*R*-1P1B** with the replacement of *R*-1P1B by *R*-4P2B.

Preparing of **CMOM-5-*S*-4P2B**: the same steps as **CMOM-5-*R*-1P1B** with the replacement of *R*-1P1B by *S*-4P2B.

Preparing of **CMOM-5-*R*-MPE**: A few single crystals of acetonitrile exchanges **CMOM-5** were soaked in 0.5 mL acetonitrile solution which contains 40 μL *R*-MPE. The screw cap was loosened to enable slow evaporation of acetonitrile for 3 days.

Preparing of **CMOM-5-*S*-MPE**: the same steps as **CMOM-5-*R*-MPE** with the replacement of *R*-1PE by *S*-MPE.

Preparing of **CMOM-5-*R*-MM**: the same steps as **CMOM-5-*R*-1P1B** with the replacement of *R*-1P1B by *R*-MM.

Preparing of **CMOM-5-*S*-MM**: the same steps as **CMOM-5-*R*-1P1B** with the replacement of *R*-1P1B by *S*-MM.

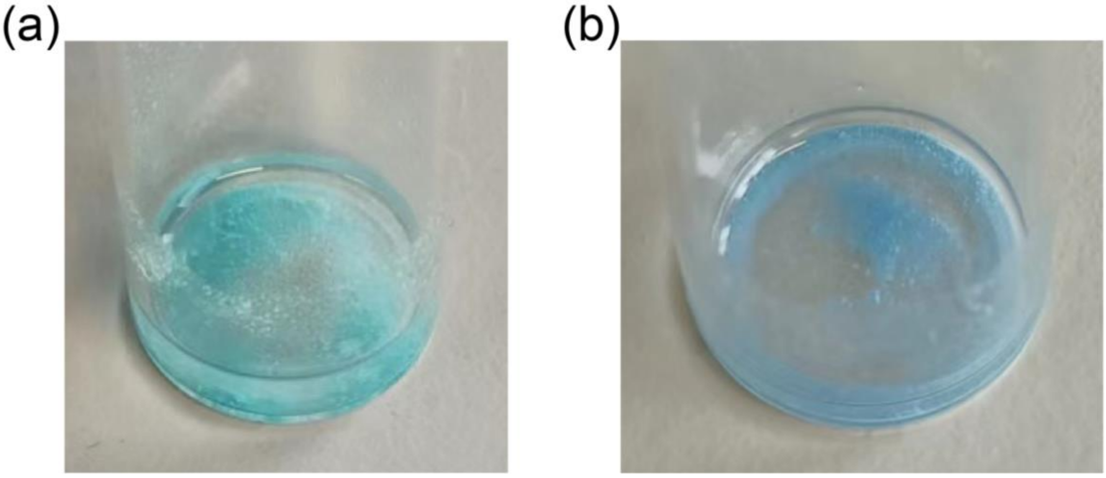

Figure S1. Crystals in the solution for crystalline sponge experiment. (a) Blue single crystals of acetonitrile exchanged **CMOM-5** were soaked in the acetonitrile solution of *S*-MM at the beginning. (b) The single crystals in the solution after solvent evaporation for 3 days.

Chiral Resolution Experiment

Dissolving the racemic 1P1B (400 μL), 4P2B (400 μL), MM (400 mg) or MPE (400 μL) in 0.5 mL acetonitrile to prepare racemic solutions. Acetonitrile exchanged **CMOM-5** crystals (90 mg) were immersed into those racemic solutions for 5 days without stirring or shaking. Subsequently, crystals were filtered and washed with ethyl acetate (3 × 1 mL) then n-hexane (3 × 10 mL) to remove the residual chiral substance on the surface of the crystals. Guest molecules were extracted by soaking the crystals in 10 mL methanol for 3 days, then crystals were filtered and washed with methanol (3 × 10 mL). The filtrates were combined, then the solvent was removed by rotary evaporator and re-dissolved in 1 mL methanol to be analyzed by chiral HPLC to determine the *ee* (enantiomeric excess) values. The *ee* values of the binary isomeric systems were calculated by the following formula:

$$ee = \frac{F_{(+)} - F_{(-)}}{F_{(+)} + F_{(-)}} \times 100\%$$

Herein, *ee* stands for the *ee* value, F(+) and F(-) stand for the mole or weight fractions of the isomers relatively more and less respectively.

Analysis of USFDA-approved Drugs

USFDA-approved (USFDA = U.S. Food and Drug Administration) drugs from 2018 to 2022 were publicized in the following websites:

1. https://www.fda.gov/drugs/new-drugs-fda-cders-new-molecular-entities-and-new-therapeutic-biological-products/novel-drug-approvals-2018
2. https://www.fda.gov/drugs/new-drugs-fda-cders-new-molecular-entities-and-new-therapeutic-biological-products/novel-drug-approvals-2019
3. https://www.fda.gov/drugs/new-drugs-fda-cders-new-molecular-entities-and-new-therapeutic-biological-products/novel-drug-approvals-2020
4. https://www.fda.gov/drugs/new-drugs-fda-cders-new-molecular-entities-and-new-therapeutic-biological-products/novel-drug-approvals-2021
5. https://www.fda.gov/drugs/new-drugs-fda-cders-new-molecular-entities-and-new-therapeutic-biological-products/novel-drug-approvals-2022

Table S1. Statistic details of the USFDA-approved drugs from 2018 to 2022.

Year of approval	Number of Approved Drugs	Number of Small Molecules	Number of Homochiral Compound Containing Drugs	Number of Racemate Containing Drugs	Ratio of Racemates	Ratio of Homochiral/Total
2022	37	21	14	1	2.7%	37.8%
2021	50	32	22	2	4%	44.0%
2020	53	38	20	2	3.8%	37.7%
2019	48	36	21	0	0%	43.8%
2018	59	40	24	4	6.8%	40.7%
Total	247	167	101	9	3.6%	40.9%

CSD Data mining

Cambridge Structural Database (CSD) data mining was conducted through ConQuest software (version 2022.3.0): CSD database version 5.43 (November 2021) and the updates March 2022, June 2022, September 2022 and November 2022.[2]

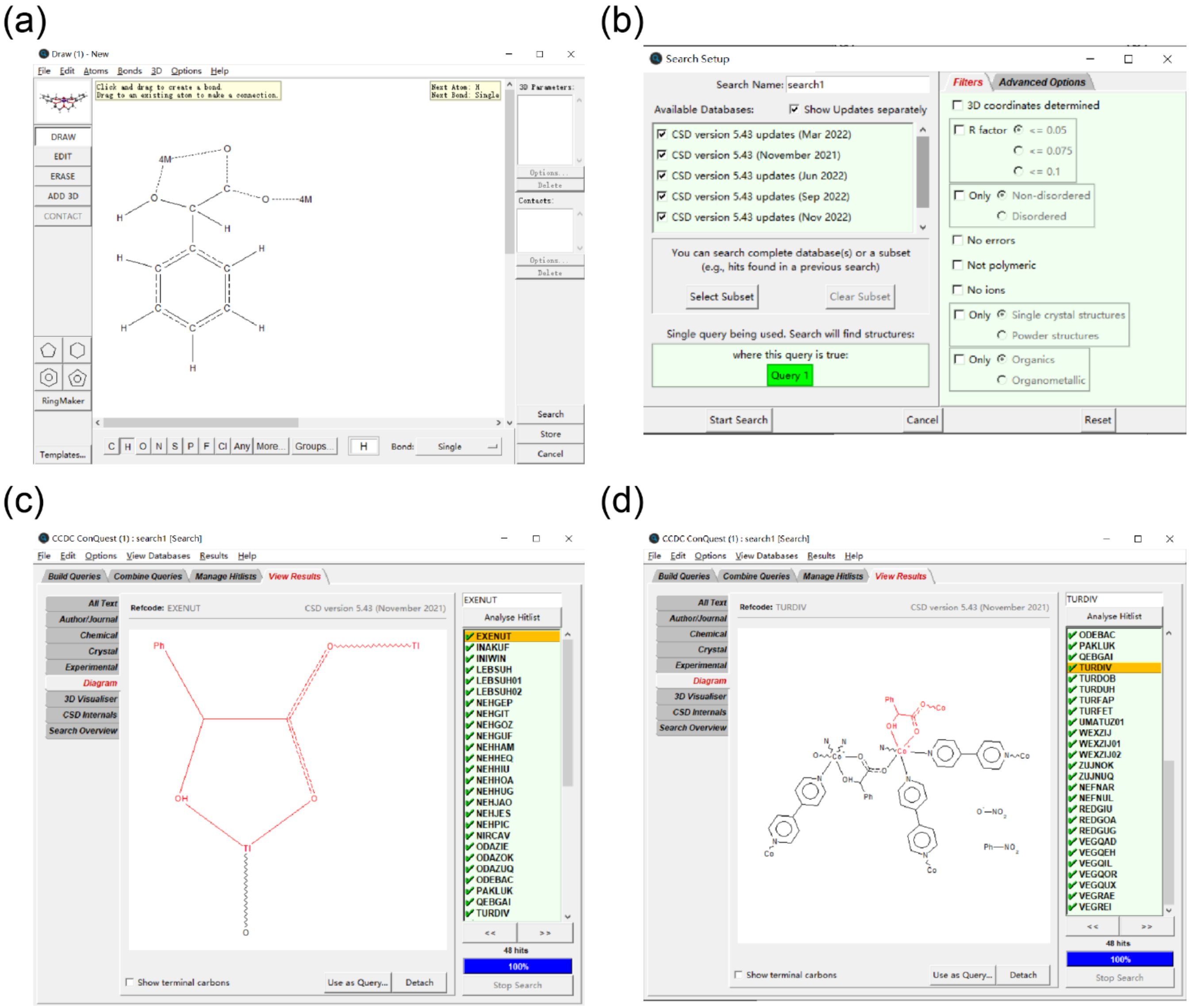

Figure S2. CSD database survey results for the mandelate ligands coordinated structures: (a) the input image; (b) search setup details; (c, d) search results.

Table S2. Summary of RBB-based compounds archived in the CSD that are based upon mandelate ligands.

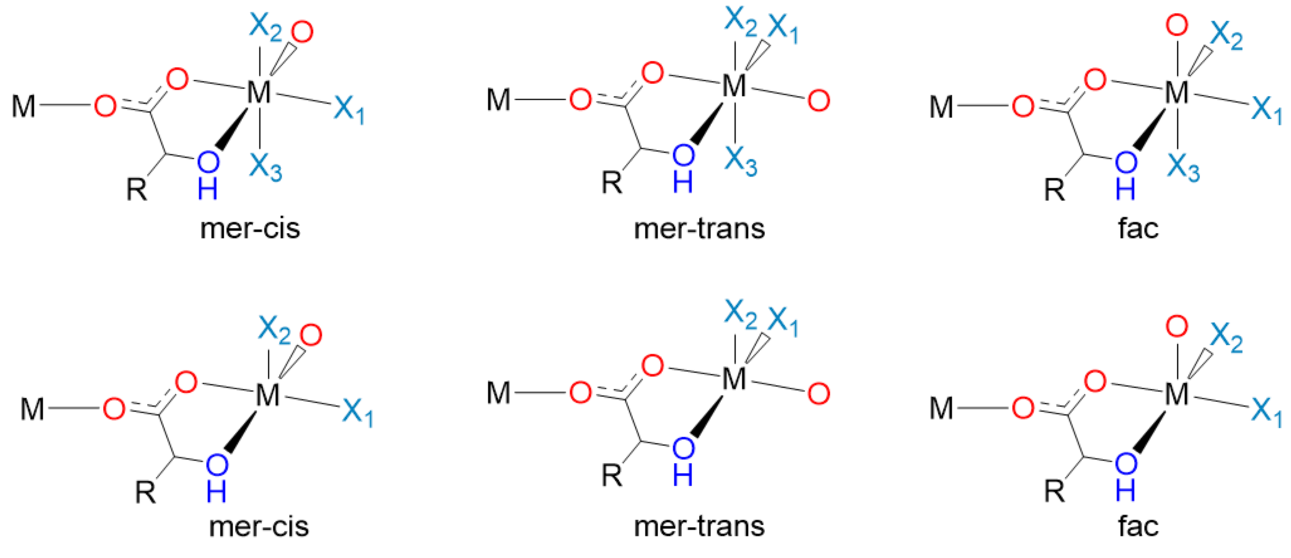

Refcode	Diagram	Formula	Dimension	Coordination Number of Central Metal Cation	Stereoisomerism	Reference
EXENUT	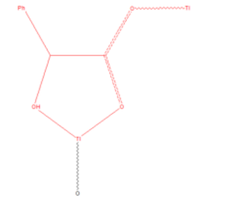	{[Tl(mandelate)]}$_n$	1	3	-	3
INIWIN	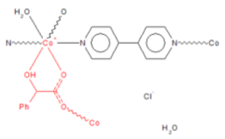	{[Co(*S*-mandelate)(bipy)(H_2O)](Cl)(H_2O)}$_n$	2 (**sql**)	6	mer-cis	4
LEBSUH02	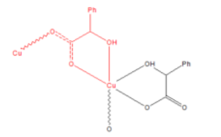	{[Cu(mandelate)$_2$]}$_n$	1	5	mer-cis	5
NEHGEP	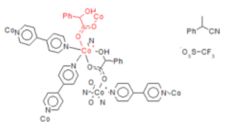	{[Co(*S*-mandelate)(bipy)$_{1.5}$](OTf)(2-phenylpropanenitrile)$_{0.41}$}$_n$	3 (**bnn**)	6	mer-trans	6
NEHGIT	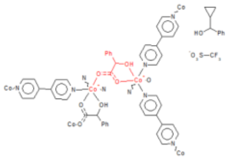	{[Co(*S*-mandelate)(bipy)$_{1.5}$](OTf)(α-cyclopropylbenzyl alcohol)$_{0.5}$}$_n$	3 (**bnn**)	6	mer-trans	6

NEHGOZ	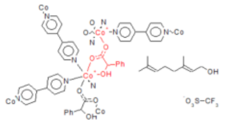	{[Co(*S*-mandelate)(bipy)$_{1.5}$](OTf)(geraniol)$_{0.5}$}$_n$	3 (**bnn**)	6	mer-trans	6
NEHGUF	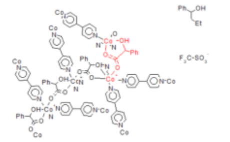	{[Co(*S*-mandelate)(bipy)$_{1.5}$](OTf)(*S*-1P1B)$_{0.42}$}$_n$	3 (**bnn**)	6	mer-trans	6
NEHHAM	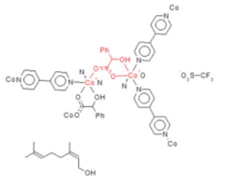	{[Co(*S*-mandelate)(bipy)$_{1.5}$](OTf)(nerol)$_{0.425}$}$_n$	3 (**bnn**)	6	mer-trans	6
NEHHEQ	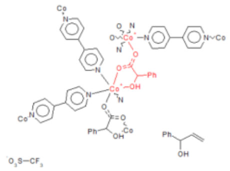	{[Co(*S*-mandelate)(bipy)$_{1.5}$](OTf)(α-vinylbenzyl alcohol)$_{0.5}$}$_n$	3 (**bnn**)	6	mer-trans	6
NEHHIU	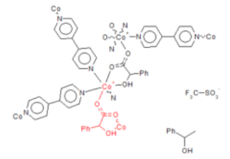	{[Co(*S*-mandelate)(bipy)$_{1.5}$](OTf)(1-phenylethanol)$_{0.5}$}$_n$	3 (**bnn**)	6	mer-trans	6
NEHHOA	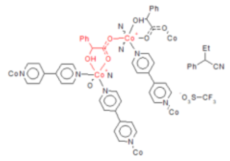	{[Co(*S*-mandelate)(bipy)$_{1.5}$](OTf)(2-phenylbutyronitrile)$_{0.455}$}$_n$	3 (**bnn**)	6	mer-trans	6
NEHHUG	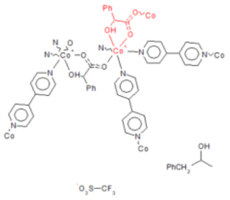	{[Co(*S*-mandelate)(bipy)$_{1.5}$](OTf)(1-phenyl-2-propanol)$_{0.5}$}$_n$	3 (**bnn**)	6	mer-trans	6
NEHJAO	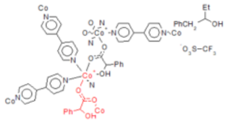	{[Co(*S*-mandelate)(bipy)$_{1.5}$](OTf)(1-phenyl-2-butanol)$_{0.39}$}$_n$	3 (**bnn**)	6	mer-trans	6
NEHJES	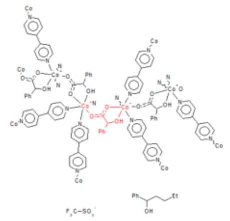	{[Co(*S*-mandelate)(bipy)$_{1.5}$](OTf)(*S*-1-phenyl-2-butanol)$_{0.5}$}$_n$	3 (**bnn**)	6	mer-trans	6

NEHPIC	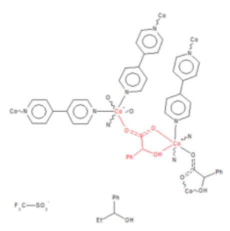	{[Co(*S*-mandelate)(bipy)$_{1.5}$](OTf)(*S*-1-phenylpropanol)$_{0.5}$}$_n$	3 (**bnn**)	6	mer-trans	6
NIRCAV	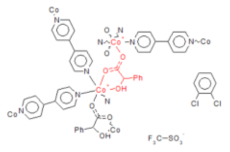	{[Co(*S*-mandelate)(bipy)$_{1.5}$](OTf)(1,2-dichlorobenzene)$_{0.95}$}$_n$	3 (**bnn**)	6	mer-trans	6
ODAZIE	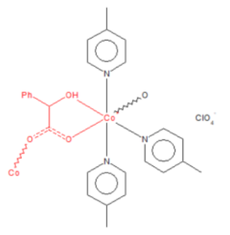	{[Co(*R*-mandelate)(4-methylpyridine)$_3$](ClO_4)}$_n$	1	6	mer-trans	7
ODAZOK	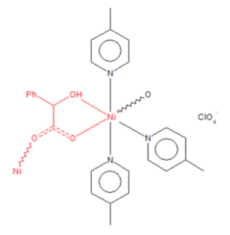	{[Ni(*R*-mandelate)(4-methylpyridine)$_3$](ClO_4)}$_n$	1	6	mer-trans	7
ODAZUQ	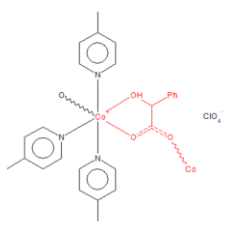	{[Co(*R*-mandelate)(4-methylpyridine)$_3$](ClO_4)}$_n$	1	6	mer-trans	7
ODEBAC	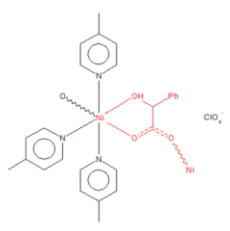	{[Ni(*R*-mandelate)(4-methylpyridine)$_3$](ClO_4)}$_n$	1	6	mer-trans	7
QEBGAI	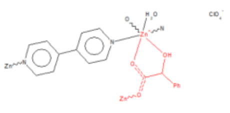	{[Zn(*S*-mandelate)(bipy)(H_2O)](ClO_4)}$_n$	2 (**sql**)	6	mer-cis	8
TURDIV	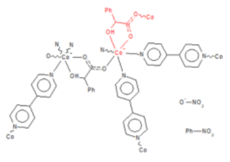	{[Co(*R*-mandelate)(bipy)$_{1.5}$](NO_3)(notribenzene)$_{1.205}$}$_n$	3 (**bnn**)	6	mer-trans	9

TURDOB	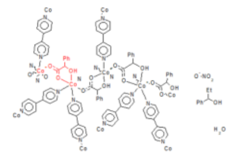	{[Co(*S*-mandelate)(bipy)$_{1.5}$](NO_3)(*R*-1-phenyl-1-propanol)$_{0.605}$(H_2O)$_{0.3625}$}$_n$	3 (**bnn**)	6	mer-trans	9
TURDUH	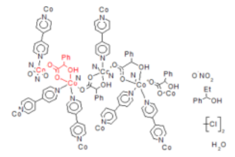	{[Co(*S*-mandelate)(bipy)$_{1.5}$](NO_3)(*S*-1-phenyl-1-propanol)$_{0.635}$(CH_2Cl_2)$_{0.1475}$(H_2O)$_{0.085}$}$_n$	3 (**bnn**)	6	mer-trans	9
TURFAP	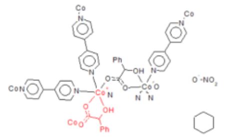	{[Co(*S*-mandelate)(bipy)$_{1.5}$](NO_3)(*cyclohexane*)$_{0.86}$}$_n$	3 (**bnn**)	6	mer-trans	9
TURFET	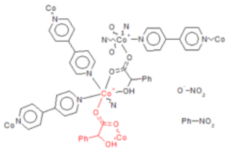	{[Co(*S*-mandelate)(bipy)$_{1.5}$](NO_3)(*notribenzene*)$_{0.765}$}$_n$	3 (**bnn**)	6	mer-trans	9
UMATUZ01	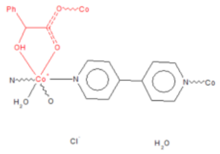	{[Co(*R*-mandelate)(bipy)(H_2O)](Cl)(H_2O)}$_n$	2 (**sql**)	6	mer-cis	4
ZUJNOK	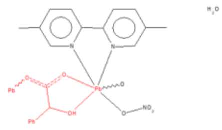	{[Pb(*S*-mandelate)(5,5'-dimethyl-2,2'-bipyridine)(NO_3)](H_2O)}$_n$	1	6	mer-trans	10
ZUJNUQ	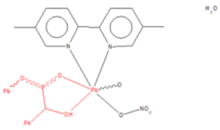	{[Pb(*R*-mandelate)(5,5'-dimethyl-2,2'-bipyridine)(NO_3)](H_2O)}$_n$	1	6	mer-trans	10
NEFNAR	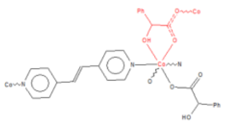	{[Co(*R*-mandelate)$_2$(4,4'-(ethene-1,2-diyl)dipyridine)]}$_n$	2 (**sql**)	6	mer-trans	11
NEFNUL	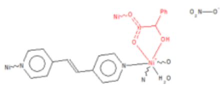	{[Ni(*R*-mandelate)(4,4'-(ethene-1,2-diyl)dipyridine)(H_2O)](NO_3)}$_n$	2 (**sql**)	6	mer-cis	11
REDGIU	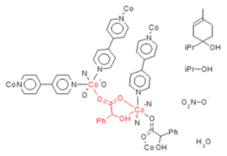	{[Co(*R*-mandelate)(bipy)$_{1.5}$](NO_3)(*S*-terpinen-4-ol)$_{0.5}$(*i-propanol*)$_{0.3}$}$_n$	3 (**bnn**)	6	mer-trans	12

REDGOA	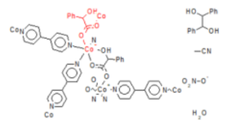	{[Co(*S*-mandelate)(bipy)$_{1.5}$](NO$_3$)(*R,R-1,2-diphenylethane-1,2-diol*)$_{0.5}$(CH$_3$CN)$_{0.825}$(H$_2$O)$_{0.328}$}$_n$	3 (**bnn**)	6	mer-trans	12
REDGUG	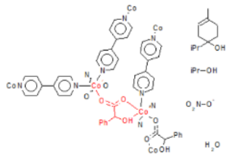	{[Co(*S*-mandelate)(bipy)$_{1.5}$](NO$_3$)(*R*-terpinen)$_{0.5}$(i-propanol)$_{0.5}$(H$_2$O)$_{0.88}$}$_n$	3 (**bnn**)	6	mer-trans	12
VEGQAD	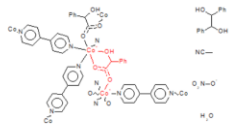	{[Co(*R*-mandelate)(bipy)$_{1.5}$](NO$_3$)(*S*,*S*-1,2-diphenylethane-1,2-diol)$_{0.5}$(CH$_3$CN)$_{0.5}$(H$_2$O)$_{0.175}$}$_n$	3 (**bnn**)	6	mer-trans	12
VEGQEH	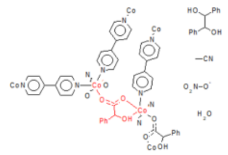	{[Co(*R*-mandelate)(bipy)$_{1.5}$](NO$_3$)(*R*,*S*-1,2-diphenylethane-1,2-diol)$_{0.5}$(CH$_3$CN)$_{0.5}$(H$_2$O)$_{0.3}$}$_n$	3 (**bnn**)	6	mer-trans	12
VEGQIL	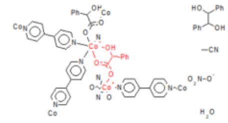	{[Co(*R*-mandelate)(bipy)$_{1.5}$](NO$_3$)(*R*,*R*-1,2-diphenylethane-1,2-diol)$_{0.5}$(CH$_3$CN)$_{0.5}$(H$_2$O)$_{0.118}$}$_n$	3 (**bnn**)	6	mer-trans	12
VEGQOR	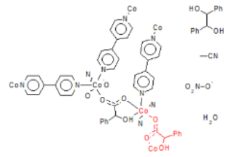	{[Co(*S*-mandelate)(bipy)$_{1.5}$](NO$_3$)(*S*,*S*-1,2-diphenylethane-1,2-diol)$_{0.5}$(CH$_3$CN)$_{0.175}$(H$_2$O)$_{0.434}$}$_n$	3 (**bnn**)	6	mer-trans	12
VEGQUX	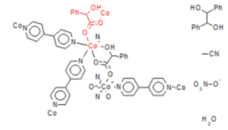	{[Co(*S*-mandelate)(bipy)$_{1.5}$](NO$_3$)(*R*,*S*-1,2-diphenylethane-1,2-diol)$_{0.5}$(CH$_3$CN)$_{0.5}$(H$_2$O)$_{0.247}$}$_n$	3 (**bnn**)	6	mer-trans	12
VEGRAE	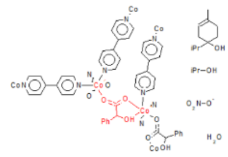	{[Co(*S*-mandelate)(bipy)$_{1.5}$](NO$_3$)(*S*-terpinen-4-ol)$_{0.5}$(i-propanol)$_{0.5}$(H$_2$O)$_{0.0935}$}$_n$	3 (**bnn**)	6	mer-trans	12
VEGREI	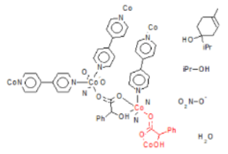	{[Co(*S*-mandelate)(bipy)$_{1.5}$](NO$_3$)(*R*-terpinen-4-ol)$_{0.5}$(i-propanol)$_{0.3395}$(H$_2$O)$_{0.2445}$}$_n$	3 (**bnn**)	6	mer-trans	12

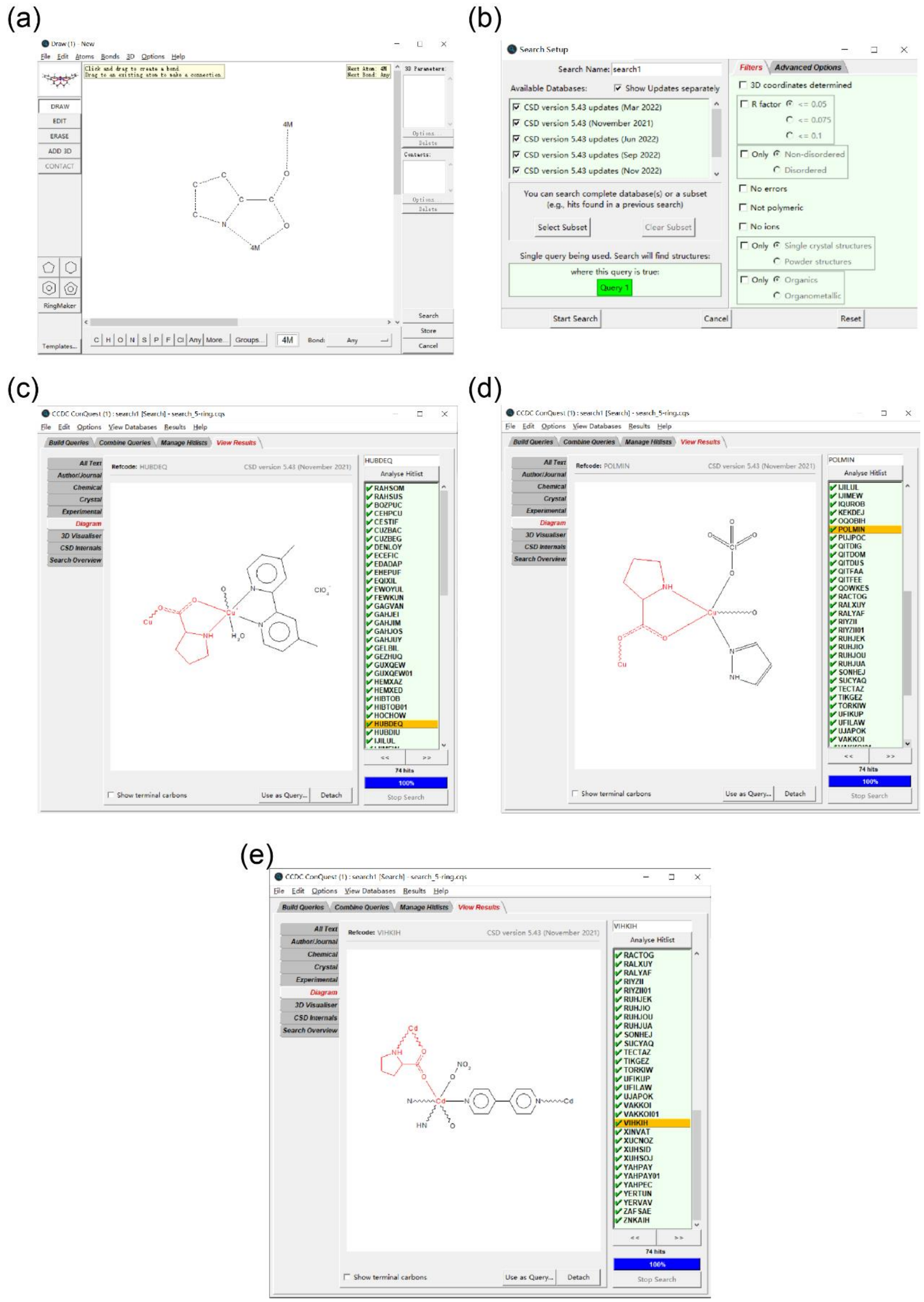

Figure S3. CSD database survey results for the prolinato ligands coordinated structures: (a) the input image; (b) search setup details; (c, d, e) results.

Table S3. Summary of RBB-based compounds archived in the CSD that are based upon prolinato ligands.

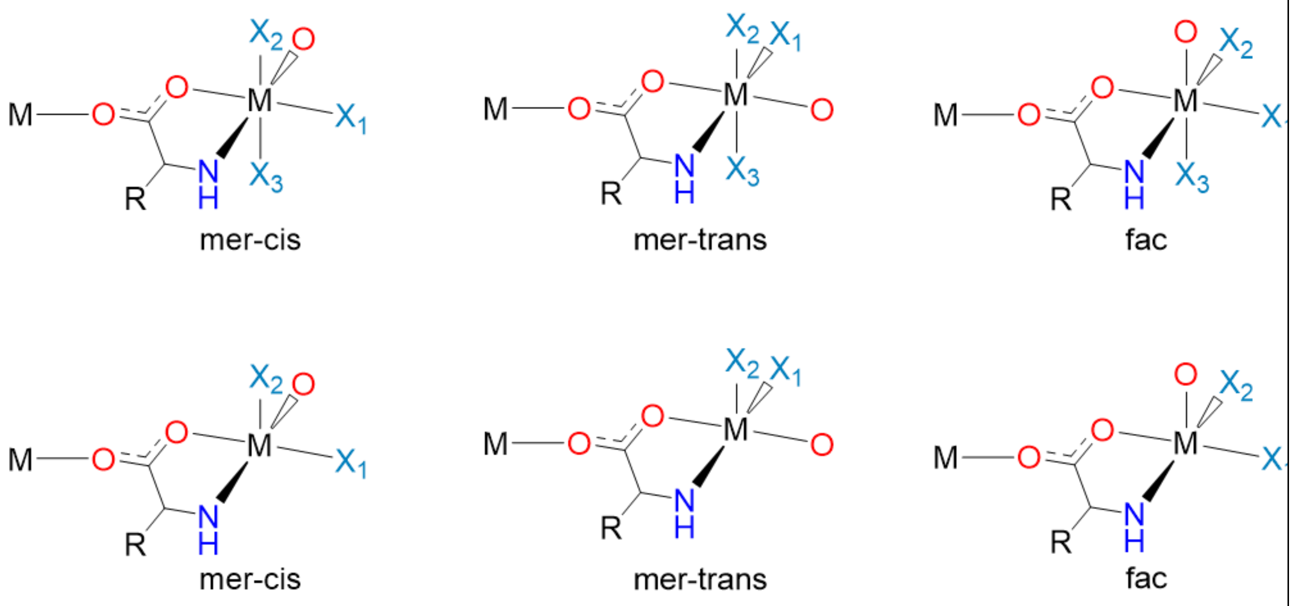

Refcode	Diagram	Formula	Dimension	Coordination Number of Central Metal Cation	Stereoisomerism	Reference
RAHSOM	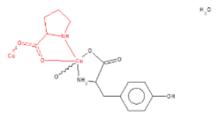	{[Cu(d-prolinato)(d-tyrosinato)](H_2O)}$_n$	1	5	fac-	13
RAHSUS	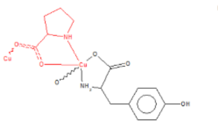	{[Cu(l-prolinato)(l-tyrosinato)](H_2O)}$_n$	1	5	fac-	13
FEWKUN	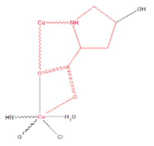	{[Cu(4-OH-prolinato)Cl(H_2O)]}$_n$	1	6	mer-cis	14
GAGVAN	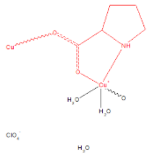	{[Cu(l-prolinato)(H_2O)$_2$](ClO_4)(H_2O)}$_n$	1	5	mer-trans	15
HIBTOB	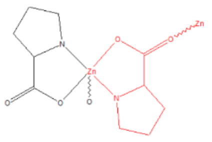	{[Zn(l-prolinato)$_2$]}$_n$	1	5	fac-	16
HIBTOB01	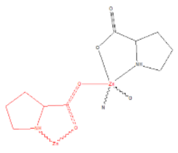	{[Zn(l-prolinato)$_2$]}$_n$	1	5	fac-	17

HUBDEQ	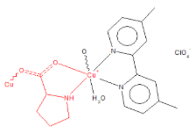	{[Cu(prolinato)(4,4'-dimethyl-2,2'-bipyridine)(H_2O)](ClO_4)}$_n$	1	6	fac-	18
HUBDIU	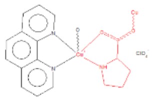	{[Cu(prolinato)(1,10-phenanthroline)](ClO_4)}$_n$	1	5	fac-	18
POLMIN	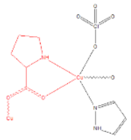	{[Cu(prolinato)(pyrazole)(ClO_4)]}$_n$	1	5	mer-trans	19
VIHKIH	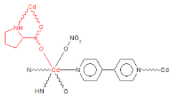	{[Cd(l-prolinato)(bipy)(NO_3)]}$_n$	2 (**sql** net)	6	mer-trans	20
YAHPAY	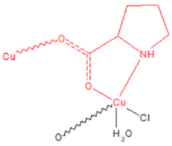	{[Cu(l-prolinato)Cl]}$_n$	1	5	fac-	21
YAHPAY01	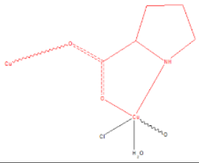	{[Cu(l-prolinato)Cl]}$_n$	1	5	fac-	22
YAHPEC	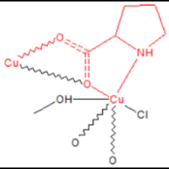	{[Cu(l-prolinato)(MeOH)Cl]}$_n$	1	6	mer-cis	21
ZAFSAE	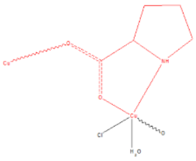	{[Cu(l-prolinato)(H_2O)Cl]}$_n$	1	5	fac-	23

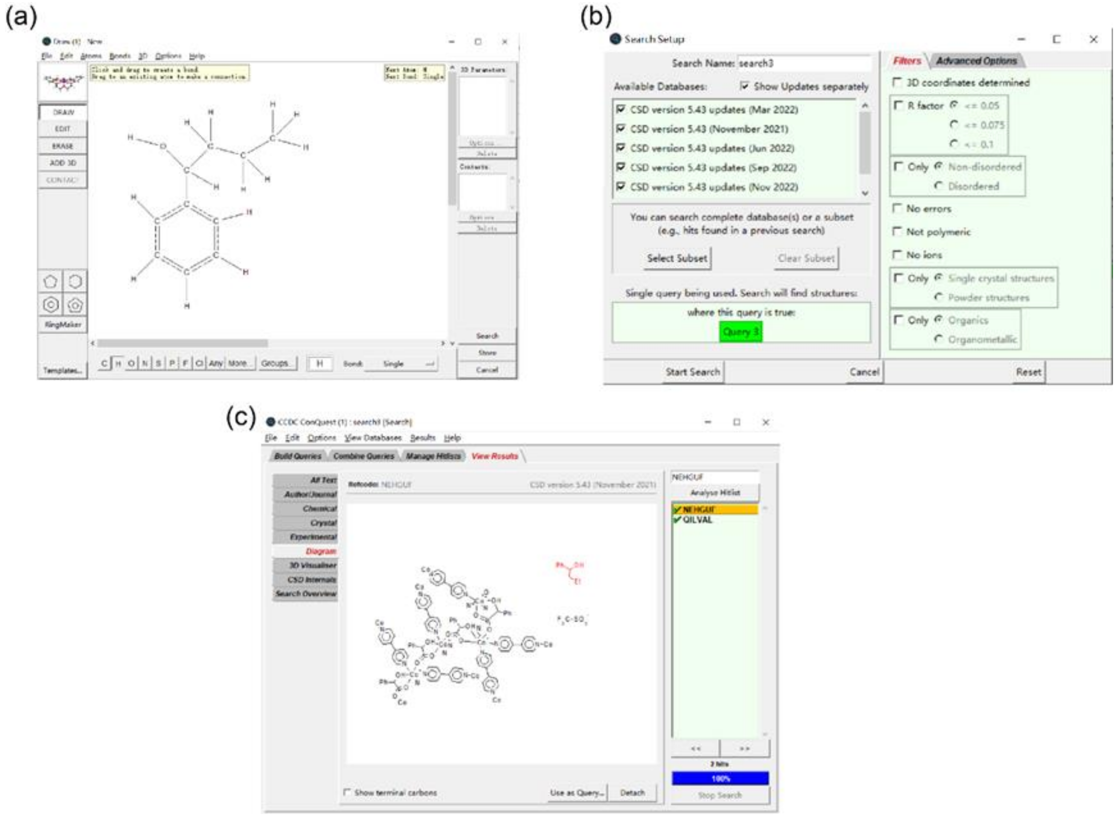

Figure S4. CSD database survey results for the 1P1B contained crystals: (a) the input image; (b) search setup details; (c) search results.

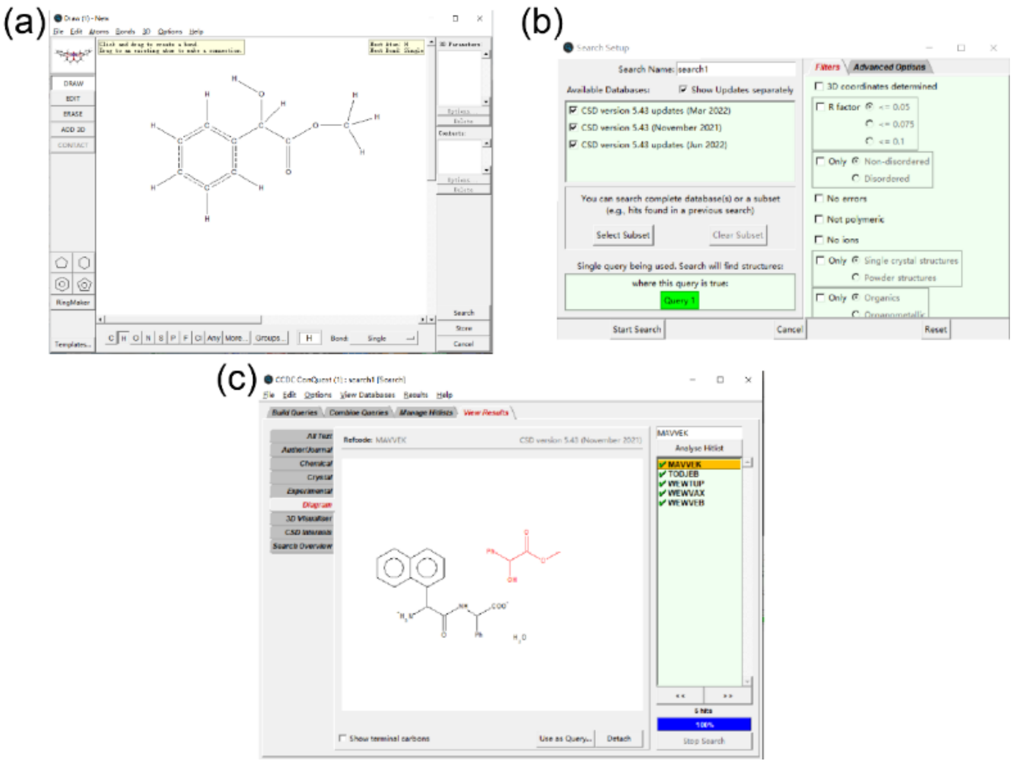

Figure S5. CSD database survey results for the MM contained crystals: (a) the input image; (b) search setup details; (c) search results.

Table S4. Summary of compounds archived in the CSD that contain the chiral ligands or guests.

Research Object	Number of Results	Refcode	Diagram	Occupancy	Note	Reference
IDEC and any metal	0	-	-		-	-
1P1B	2	NEHGUF	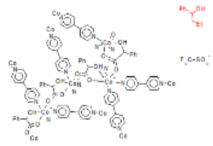	0.88/0.8	S-1P1B solved in CMOM-3S	6
		QILVAL	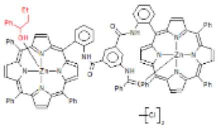	0.8+0.2 (two-fold racemically disordered)	racemate, hydroxy coordinated	24
4P2B	0	-	-		-	-
MPE	0	-	-		-	-
MM	5	MAVVEK	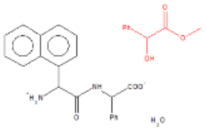	1	S-MM, co-crystal	25
		TODJEB	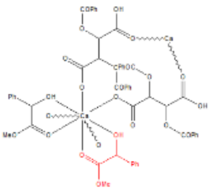	1	R-MM, carbonyl and hydroxy coordinated	26
		WEWTUP	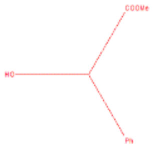	1	R-MM	27
		WEWVAX	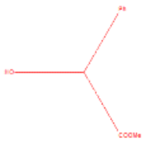	1	S-MM	27
		WEWVEB	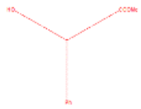	1	Racemic MM	27

Crystallographic Analysis Methods and Tables

Suitable single crystals of all compounds were chosen for single crystal X-ray diffraction measurements. All single crystal data were collected on a Bruker D8 Quest diffractometer equipped with a IμS micro-focus Cu anode Cu Kα (λ = 1.54178 Å) and Photon II detector. For low temperature measurements, an open-flow nitrogen attachment from Oxford Cryosystems was used. The data was

indexed in APEX4 (v2021.10-0). Integration was performed by SAINT V8.40A in APEX4. Absorption correction was performed by multi-scan method using SADABS in APEX4.[28] Space group determinations were performed with the assistance of XPREP, as implemented in APEX4.

Structures were solved using the intrinsic phasing method (SHELXT) and refined on F^2 using SHELXL least squares method as run in OLEX2 v1.3 programs packages.[29-31] All non-hydrogen atoms on the frameworks were refined anisotropically. Hydrogen atoms were added geometry at idealized positions and refined using the riding model. The thermal parameters and geometry of the guest molecules were restricted to reasonable limits using the ISOR, SIMU, DFIX, and FLAT instructions. The occupancies of the chiral isomers were determined by taking into consideration of the MASK-calculated electrons and the NMR spectra reflected ratio among the guests and the ligands. Crystallographic data for all compounds are summarized in the following crystallographic Tables. The SQUEEZE subroutine of the PLATON software suite was used to remove the scattering from the highly disordered guest molecules.[32] All crystal structures have been deposited with the Cambridge Crystallographic Data Centre (CCDC deposition numbers 2157776-2157785, 2157797-2157799, and 2236740).

Table S5. Crystallographic data of **CMOM-5** and **CMOM-5-MeOH**.

	**CMOM-5**	**CMOM-5-MeOH**
Formula	$C_{19}H_{18}N_4NiO_6$	$C_{19}H_{18}N_4NiO_6$
Formula weight	457.08	457.08
Temperature/K	150.0	150.0
Crystal system	orthorhombic	orthorhombic
Space group	$P2_12_12_1$	$P2_12_12_1$
a/Å	9.9260(3)	9.8738(4)
b/Å	15.3099(5)	15.3558(7)
c/Å	18.9509(5)	18.6497(8)
α/°	90	90
β/°	90	90
γ/°	90	90
Volume/Å^3	2879.89(15)	2827.7(2)
Z	4	4
ρ_{calc}g/cm^3	1.054	1.074
μ/mm^{-1}	1.249	1.273
F(000)	944.0	944.0
Crystal size/mm^3	0.095 × 0.05 × 0.047	0.216 × 0.068 × 0.065
Radiation	CuKα (λ = 1.54178)	CuKα (λ = 1.54178)
2θ range for data collection/°	7.422 to 136.62	7.458 to 133.128
Index ranges	$-11 \le h \le 8$, $-17 \le k \le 18$, $-22 \le l \le 20$	$-10 \le h \le 11$, $-18 \le k \le 13$, $-22 \le l \le 21$
Reflections Collected	19757	21263
Independent reflections	5260 [R_{int} = 0.0863, R_{sigma} = 0.0658]	4988 [R_{int} = 0.1413, R_{sigma} = 0.1010]
Data / restraints / parameters	5260/157/275	4988/452/272
Goodness-of-fit on F^2	1.033	1.014
Final R indexes [I > = 2σ(I)]	R_1 = 0.0572, wR_2 = 0.1528	R_1 = 0.0716, wR_2 = 0.1843
Final R indexes [all data]	R_1 = 0.0765, wR_2 = 0.1707	R_1 = 0.1237, wR_2 = 0.2240
Largest diff. peak/hole / e Å^{-3}	0.64/-0.47	0.56/-0.47
Flack parameter	0.01(3)	0.10(5)
CCDC number	2157776	2157777

Table S6. Crystallographic data of **CMOM-5-CH_3CN-*α*** and **CMOM-5-CH_3CN-*β***.

	**CMOM-5-CH_3CN-*α***	**CMOM-5-CH_3CN-*β***
Formula	$C_{19}H_{18}N_4NiO_6$	$C_{23}H_{24}N_6NiO_6$
Formula weight	457.08	539.19
Temperature/K	150.0	150.0
Crystal system	orthorhombic	orthorhombic
Space group	$P2_12_12_1$	$P2_12_12_1$
a/Å	9.8573(4)	9.8777(3)
b/Å	14.9224(9)	11.9911(4)
c/Å	18.9879(9)	21.0983(7)
α/°	90	90
β/°	90	90
γ/°	90	90
Volume/$Å^3$	2793.0(2)	2498.98(14)
Z	4	4
ρ_{calc}g/cm^3	1.087	1.433
μ/mm^{-1}	1.288	1.554
F(000)	944.0	1120.0
Crystal size/mm^3	0.306 × 0.095 × 0.087	0.138 × 0.132 × 0.095
Radiation	CuKα (λ = 1.54178)	CuKα (λ = 1.54178)
2θ range for data collection/°	7.534 to 133.486	8.382 to 136.868
Index ranges	$-10 \le h \le 11, -17 \le k \le 17, -19 \le l \le 22$	$-11 \le h \le 11, -14 \le k \le 14, -21 \le l \le 25$
Reflections Collected	16264	21568
Independent reflections	4919 [R_{int} = 0.1070, R_{sigma} = 0.0871]	4583 [R_{int} = 0.0898, R_{sigma} = 0.0621]
Data / restraints / parameters	4919/282/301	4583/0/328
Goodness-of-fit on F^2	1.049	1.068
Final R indexes [I > = 2σ(I)]	R_1 = 0.0906, wR_2 = 0.2498	R_1 = 0.0509, wR_2 = 0.1102
Final R indexes [all data]	R_1 = 0.1425, wR_2 = 0.2989	R_1 = 0.0629, wR_2 = 0.1159
Largest diff. peak/hole / e $Å^{-3}$	0.67/-0.71	0.40/-0.39
Flack parameter	0.18(4)	0.04(3)
CCDC number	2157778	2157779

Table S7. Crystallographic data of **CMOM-5-Hex** and **CMOM-5-IPA_Hex**.

	**CMOM-5-Hex**	**CMOM-5-IPA_Hex**
Formula	$C_{25}H_{32}N_4NiO_6$	$C_{22}H_{26}N_4NiO_7$
Formula weight	543.25	517.18
Temperature/K	150.0	150.0
Crystal system	orthorhombic	orthorhombic
Space group	$P2_12_12_1$	$P2_12_12_1$
a/Å	9.8605(3)	9.9080(4)
b/Å	15.9717(6)	14.9893(8)
c/Å	18.0744(6)	19.1610(8)
α/°	90	90
β/°	90	90
γ/°	90	90
Volume/Å^3	2846.52(17)	2845.7(2)
Z	4	4
ρ_{calc}g/cm^3	1.268	1.207
μ/mm^{-1}	1.341	1.346
F(000)	1144.0	1080.0
Crystal size/mm^3	0.189 × 0.065 × 0.063	0.124 × 0.057 × 0.039
Radiation	CuKα (λ = 1.54178)	CuKα (λ = 1.54178)
2θ range for data collection/°	7.386 to 133.244	7.488 to 133.426
Index ranges	$-11 \leq h \leq 10$, $-18 \leq k \leq 18$, $-21 \leq l \leq 21$	$-11 \leq h \leq 11$, $-17 \leq k \leq 16$, $-22 \leq l \leq 22$
Reflections Collected	21452	25587
Independent reflections	5005 [R_{int} = 0.1498, R_{sigma} = 0.1071]	5004 [R_{int} = 0.1584, R_{sigma} = 0.0966]
Data / restraints / parameters	5005/10/316	5004/195/299
Goodness-of-fit on F^2	1.036	1.021
Final R indexes [I > = 2σ(I)]	R_1 = 0.0631, wR_2 = 0.1498	R_1 = 0.0791, wR_2 = 0.2024
Final R indexes [all data]	R_1 = 0.1236, wR_2 = 0.1906	R_1 = 0.1421, wR_2 = 0.2623
Largest diff. peak/hole / e Å^{-3}	0.46/-0.66	0.59/-0.63
Flack parameter	0.01(4)	0.02(5)
CCDC number	2157780	2157781

Table S8. Crystallographic data of **CMOM-5-*R*-1P1B** and **CMOM-5-*S*-1P1B**.

	**CMOM-5-*R*-1P1B**	**CMOM-5-*S*-1P1B**
Formula	$C_{29}H_{32}N_4NiO_7$	$C_{19}H_{18}N_4NiO_6$
Formula weight	607.29	457.08
Temperature/K	150.0	150.0
Crystal system	orthorhombic	orthorhombic
Space group	$P2_12_12_1$	$P2_12_12_1$
a/Å	10.1164(3)	9.9180(3)
b/Å	16.7688(4)	15.7345(5)
c/Å	17.2364(5)	18.3672(5)
α/°	90	90
β/°	90	90
γ/°	90	90
Volume/Å^3	2923.98(14)	2866.29(15)
Z	4	4
ρ_{calc}g/cm^3	1.380	1.059
μ/mm^{-1}	1.396	1.255
F(000)	1272.0	944.0
Crystal size/mm^3	0.268 × 0.086 × 0.075	0.189 × 0.091 × 0.079
Radiation	CuKα (λ = 1.54178)	CuKα (λ = 1.54178)
2θ range for data collection/°	7.354 to 133.236	7.398 to 133.59
Index ranges	$-11 \leq h \leq 12$, $-18 \leq k \leq 19$, $-20 \leq l \leq 19$	$-11 \leq h \leq 10$, $-18 \leq k \leq 18$, $-21 \leq l \leq 21$
Reflections Collected	25795	25741
Independent reflections	5152 [R_{int} = 0.1138, R_{sigma} = 0.0783]	5058 [R_{int} = 0.0749, R_{sigma} = 0.0498]
Data / restraints / parameters	5152/7/380	5058/107/309
Goodness-of-fit on F^2	1.080	1.031
Final R indexes [I > = 2σ(I)]	R_1 = 0.0691, wR_2 = 0.1692	R_1 = 0.0577, wR_2 = 0.1546
Final R indexes [all data]	R_1 = 0.1169, wR_2 = 0.2132	R_1 = 0.0716, wR_2 = 0.1695
Largest diff. peak/hole / e Å^{-3}	1.43/-0.59	0.62/-0.33
Flack parameter	0.04(3)	0.12(2)
CCDC number	2157782	2157783

Table S9. Crystallographic data of **CMOM-5-*R*-4P2B** and **CMOM-5-*S*-4P2B**.

	**CMOM-5-*R*-4P2B**	**CMOM-5-*S*-4P2B**
Formula	$C_{29}H_{34}N_4NiO_8$	$C_{29}H_{34}N_4NiO_8$
Formula weight	625.31	625.31
Temperature/K	150.0	150.0
Crystal system	orthorhombic	orthorhombic
Space group	$P2_12_12_1$	$P2_12_12_1$
a/Å	10.0979(3)	9.9642(3)
b/Å	16.0566(5)	16.6523(4)
c/Å	18.2095(5)	17.6594(5)
α/°	90	90
β/°	90	90
γ/°	90	90
Volume/Å^3	2952.45(15)	2930.17(14)
Z	4	4
ρ_{calc}g/cm^3	1.407	1.417
μ/mm^{-1}	1.424	1.435
F(000)	1312.0	1312.0
Crystal size/mm^3	0.325 × 0.102 × 0.098	0.289 × 0.068 × 0.055
Radiation	CuKα (λ = 1.54178)	CuKα (λ = 1.54178)
2θ range for data collection/°	7.34 to 136.486	10.192 to 140.178
Index ranges	$-11 \le h \le 12$, $-19 \le k \le 19$, $-21 \le l \le 21$	$-11 \le h \le 10$, $-20 \le k \le 20$, $-21 \le l \le 21$
Reflections Collected	52210	28929
Independent reflections	5405 [R_{int} = 0.0519, R_{sigma} = 0.0240]	5532 [Rint = 0.0503, Rsigma = 0.0359]
Data / restraints / parameters	5405/1/385	5532/26/392
Goodness-of-fit on F^2	1.042	1.030
Final R indexes [I > = 2σ(I)]	R_1 = 0.0277, wR_2 = 0.0721	R_1 = 0.0377, wR_2 = 0.0927
Final R indexes [all data]	R_1 = 0.0293, wR_2 = 0.0734	R_1 = 0.0408, wR_2 = 0.0953
Largest diff. peak/hole / e Å^{-3}	0.30/-0.31	1.16/-0.58
Flack parameter	0.018(8)	0.049(11)
CCDC number	2157784	2157785

Table S10. Crystallographic data of **CMOM-5-*R*-MPE** and **CMOM-5-*S*-MPE**.

	**CMOM-5-*R*-MPE**	**CMOM-5-*S*-MPE**
Formula	$C_{30}H_{33}N_5NiO_8$	$C_{19}H_{18}N_4NiO_6$
Formula weight	650.32	457.06
Temperature/K	150.0	150.0
Crystal system	orthorhombic	orthorhombic
Space group	$P2_12_12_1$	$P2_12_12_1$
a/Å	10.0037(8)	9.9347(2)
b/Å	16.2849(14)	16.1133(3)
c/Å	18.7428(18)	18.3285(4)
α/°	90	90
β/°	90	90
γ/°	90	90
Volume/Å^3	3053.4(5)	2934.04(10)
Z	4	4
ρ_{calc}g/cm^3	1.415	1.035
μ/mm^{-1}	1.412	1.226
F(000)	1360.0	944.0
Crystal size/mm^3	0.124 × 0.051 × 0.043	0.203 × 0.084 × 0.076
Radiation	CuKα (λ = 1.54178)	CuKα (λ = 1.54178)
2θ range for data collection/°	7.19 to 133.406	7.304 to 133.63
Index ranges	-11 ≤ h ≤ 10, -15 ≤ k ≤ 19, -14 ≤ l ≤ 22	-11 ≤ h ≤ 11, -19 ≤ k ≤ 17, -20 ≤ l ≤ 21
Reflections Collected	18453	23199
Independent reflections	5306 [R_{int} = 0.1494, R_{sigma} = 0.1160]	5177 [R_{int} = 0.0806, R_{sigma} = 0.0582]
Data / restraints / parameters	5306/0/402	5177/62/272
Goodness-of-fit on F^2	1.089	1.045
Final R indexes [I > = 2σ(I)]	R_1 = 0.0976, wR_2 = 0.2418	R_1 = 0.0551, wR_2 = 0.1457
Final R indexes [all data]	R_1 = 0.1317, wR_2 = 0.2679	R_1 = 0.0641, wR_2 = 0.1527
Largest diff. peak/hole / e Å^{-3}	1.28/-0.52	0.53/-0.36
Flack parameter	0.13(7)	0.10(2)
CCDC number	2157797	2157798

Table S11. Crystallographic data of **CMOM-5-*R*-MM** and **CMOM-5-*S*-MM**.

	**CMOM-5-*R*-MM**	**CMOM-5-*S*-MM**
Formula	$C_{19}H_{18}N_4NiO_6$	$C_{28}H_{28}N_4NiO_9$
Formula weight	457.08	623.25
Temperature/K	150.0	150.0
Crystal system	orthorhombic	orthorhombic
Space group	$P2_12_12_1$	$P2_12_12_1$
a/Å	9.9688(3)	9.8500(4)
b/Å	15.7462(5)	15.8685(7)
c/Å	18.6000(5)	18.1599(9)
α/°	90	90
β/°	90	90
γ/°	90	90
Volume/$Å^3$	2919.66(15)	2838.5(2)
Z	4	4
$\rho_{calc}g/cm^3$	1.040	1.458
μ/mm^{-1}	1.232	1.511
F(000)	944.0	1296.0
Crystal size/mm^3	0.168 × 0.066 × 0.058	0.109 × 0.035 × 0.024
Radiation	CuKα (λ = 1.54178)	CuKα (λ = 1.54178)
2θ range for data collection/°	7.356 to 136.802	7.398 to 133.36
Index ranges	$-11 \leq h \leq 10$, $-18 \leq k \leq 16$, $-22 \leq l \leq 17$	$-10 \leq h \leq 11$, $-18 \leq k \leq 13$, $-19 \leq l \leq 21$
Reflections Collected	21219	22223
Independent reflections	5198 [R_{int} = 0.0784, R_{sigma} = 0.0692]	5018 [R_{int} = 0.1391, R_{sigma} = 0.0978]
Data / restraints / parameters	5198/44/272	5018/2/389
Goodness-of-fit on F^2	1.044	1.028
Final R indexes [I > = 2σ(I)]	R_1 = 0.0696, wR_2 = 0.1781	R_1 = 0.0565, wR_2 = 0.1262
Final R indexes [all data]	R_1 = 0.0860, wR_2 = 0.1916	R_1 = 0.0932, wR_2 = 0.1488
Largest diff. peak/hole / e $Å^{-3}$	0.79/-0.43	0.39/-0.59
Flack parameter	0.15(3)	-0.10(4)
CCDC number	2157799	2236740

Structural Details

Table S12. Bond lengths and angles for **CMOM-5**.

Bond Type	Length / Å	Bond Type	Length / Å	Bond Type	Length / Å	Bond Type	Length / Å
Ni1-O2	2.041(3)	N3-C17	1.354(10)	C15-C18	1.402(11)	C4-C3	1.466(13)
Ni1-O1	2.124(5)	N3-C19	1.364(10)	C14-C11	1.399(10)	C4-C9	1.390(12)
Ni1-O3^{1}	2.045(4)	N1-C5	1.459(8)	C14-C13	1.394(10)	C17-C16	1.339(11)
Ni1-N3^{2}	2.078(6)	N1-C2	1.497(8)	C5-C6	1.376(11)	C9-C8	1.327(17)
Ni1-N1	2.121(5)	N2-C10	1.338(8)	C5-C4	1.381(11)	C7-C8	1.388(15)
Ni1-N2	2.095(5)	N2-C12	1.320(9)	C11-C10	1.352(9)	C19-C18	1.369(12)
O2-C1	1.254(7)	C1-C2	1.529(7)	C6-C7	1.406(10)	N4-O4	1.305(15)
O3-C1	1.263(6)	C15-C14	1.466(9)	C2-C3	1.549(10)	N4-O5	1.175(12)
N3-C17	1.354(10)	C15-C16	1.384(11)	C13-C12	1.363(10)	N4-O6	1.236(13)

Bond Angle	Angle / °	Bond Angle	Angle / °	Bond Angle	Angle / °	Bond Angle	Angle / °
O2-Ni1-O1	88.01(16)	C1-O2-Ni1	116.7(3)	C16-C15-C14	116.6(7)	C5-C4-C3	110.1(7)
O2-Ni1-O3^{1}	175.68(17)	C1-O3-Ni1^{3}	126.8(3)	C18-C15-C14	121.4(6)	C5-C4-C9	119.6(9)
O2-Ni1-N3^{2}	91.54(18)	C17-N3-Ni1^{4}	119.7(5)	C11-C14-C15	123.6(6)	C9-C4-C3	130.3(9)
O2-Ni1-N1	80.47(16)	C17-N3-C19	116.4(7)	C13-C14-C15	120.9(6)	C16-C17-N3	125.3(8)
O2-Ni1-N2	92.36(19)	C19-N3-Ni1^{4}	123.8(5)	C13-C14-C11	115.4(6)	N2-C12-C13	125.2(7)
O3^{1}-Ni1-N2	87.69(16)	C5-N1-Ni1	123.9(4)	C6-C5-N1	125.4(7)	C4-C3-C2	104.8(6)
O3^{1}-Ni1-N3^{2}	87.94(18)	C5-N1-C2	105.5(5)	C6-C5-C4	122.8(7)	C17-C16-C15	119.1(8)
O3^{1}-Ni1-N1	99.52(17)	C2-N1-Ni1	107.7(3)	C4-C5-N1	111.8(6)	C8-C9-C4	118.4(10)
O3^{1}-Ni1-N2	91.93(19)	C10-N2-Ni1	123.7(4)	C10-C11-C14	120.2(7)	C8-C9-C6	119.8(11)
N3^{2}-Ni1-O1	88.8(2)	C12-N2-Ni1	120.4(5)	N2-C10-C11	124.1(6)	N3-C19-C18	120.8(8)
N3^{2}-Ni1-N1	169.6(2)	C12-N2-C10	115.5(6)	C5-C6-C7	116.2(10)	C9-C8-C7	123.2(11)
N3^{2}-Ni1-N2	89.8(2)	O2-C1-O3	125.3(5)	N1-C2-C1	109.7(5)	C19-C18-C15	121.5(8)
N1-Ni1-O1	84.30(19)	O2-C1-C3	118.6(4)	N1-C2-C3	105.6(5)	O5-N4-O4	112.3(16)
N2-Ni1-O1	178.6(2)	O3-C1-C2	116.0(5)	C1-C2-C3	112.6(5)	O5-N4-O6	124.6(18)
N2-Ni1-N1	97.1(2)	C16-C15-C14	121.9(6)	C12-C13-C14	119.3(6)	O6-N4-O4	123.1(17)

^{1}1/2+x, 1/2-y, 1-z; ^{2}1-x, -1/2+y, 1/2-z; ^{3}-1/2+x, 1/2-y, 1-z; ^{4}1-x, 1/2+y, 1/2-z.

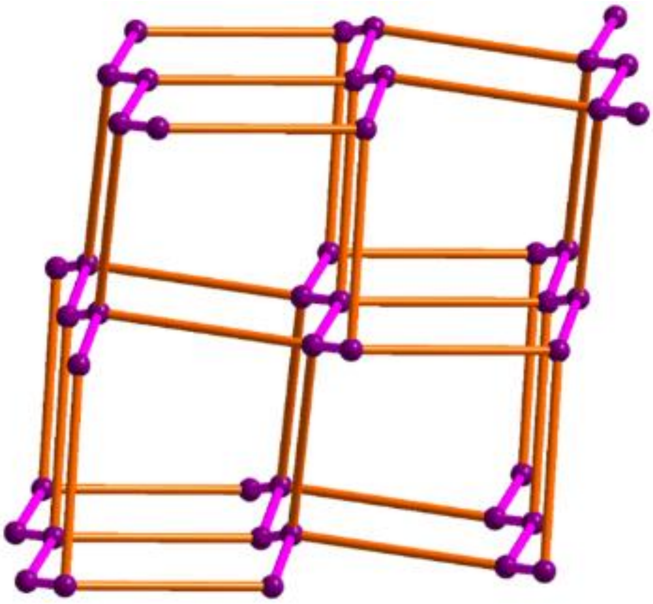

Figure S6. The topology plot of the ***dia*** net. The point symbol is {6^6}, and the vertex symbol is {6(2).6(2).6(2).6(2).6(2).6(2)}.

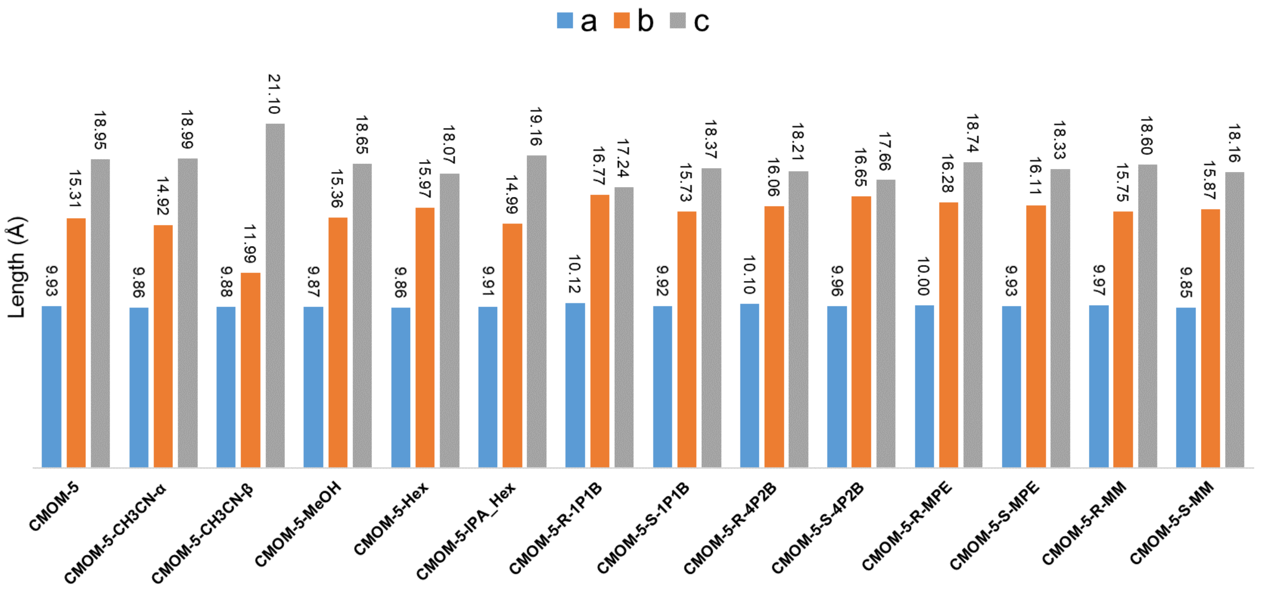

Figure S7. The length of *a*, *b*, and *c* edges of the unit cells of **CMOM-5** with different guest molecules loaded.

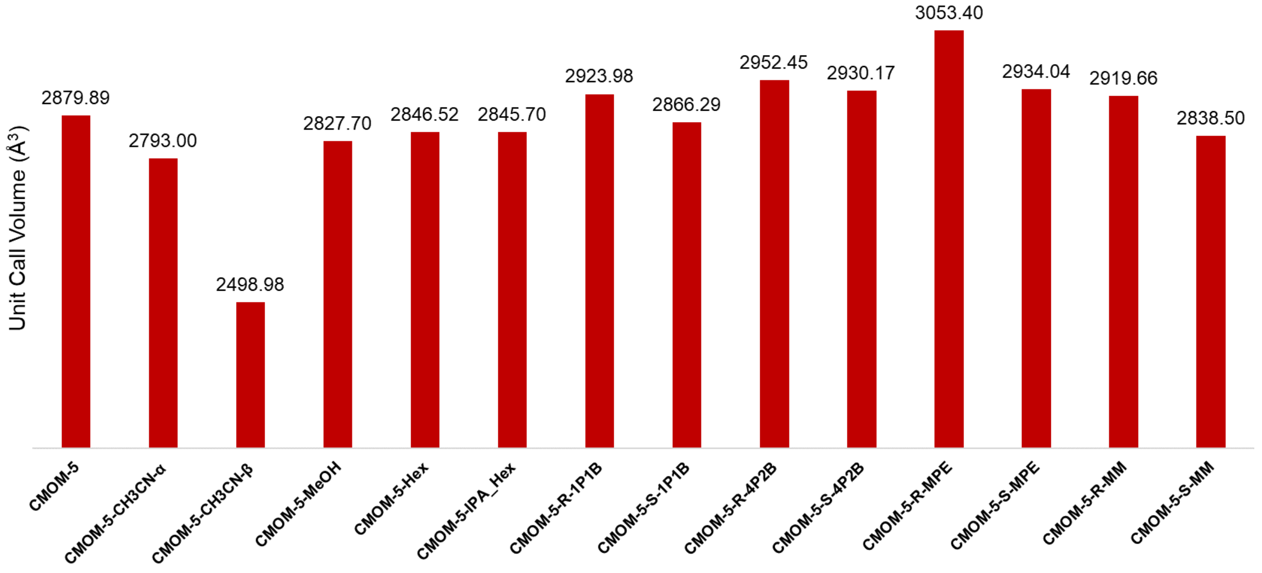

Figure S8. The unit cell volumes of **CMOM-5** with different guest molecules loaded.

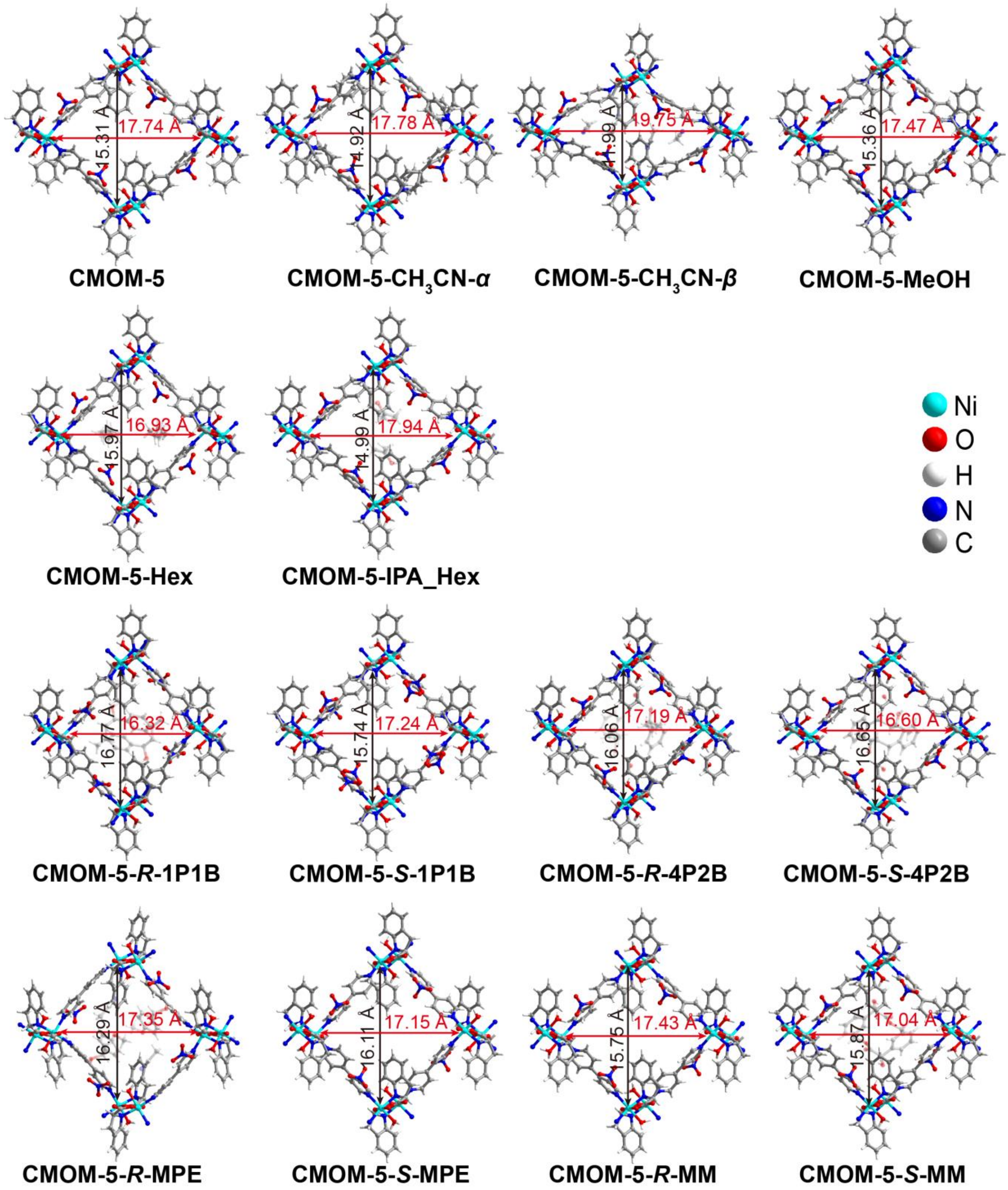

Figure S9. Phases of **CMOM-5** with different guest molecules loaded. Structures of each phase were viewed in the direction of the infinite channels. The distance of the nickel anions on the opposite side of the quadrangular channel was labelled.

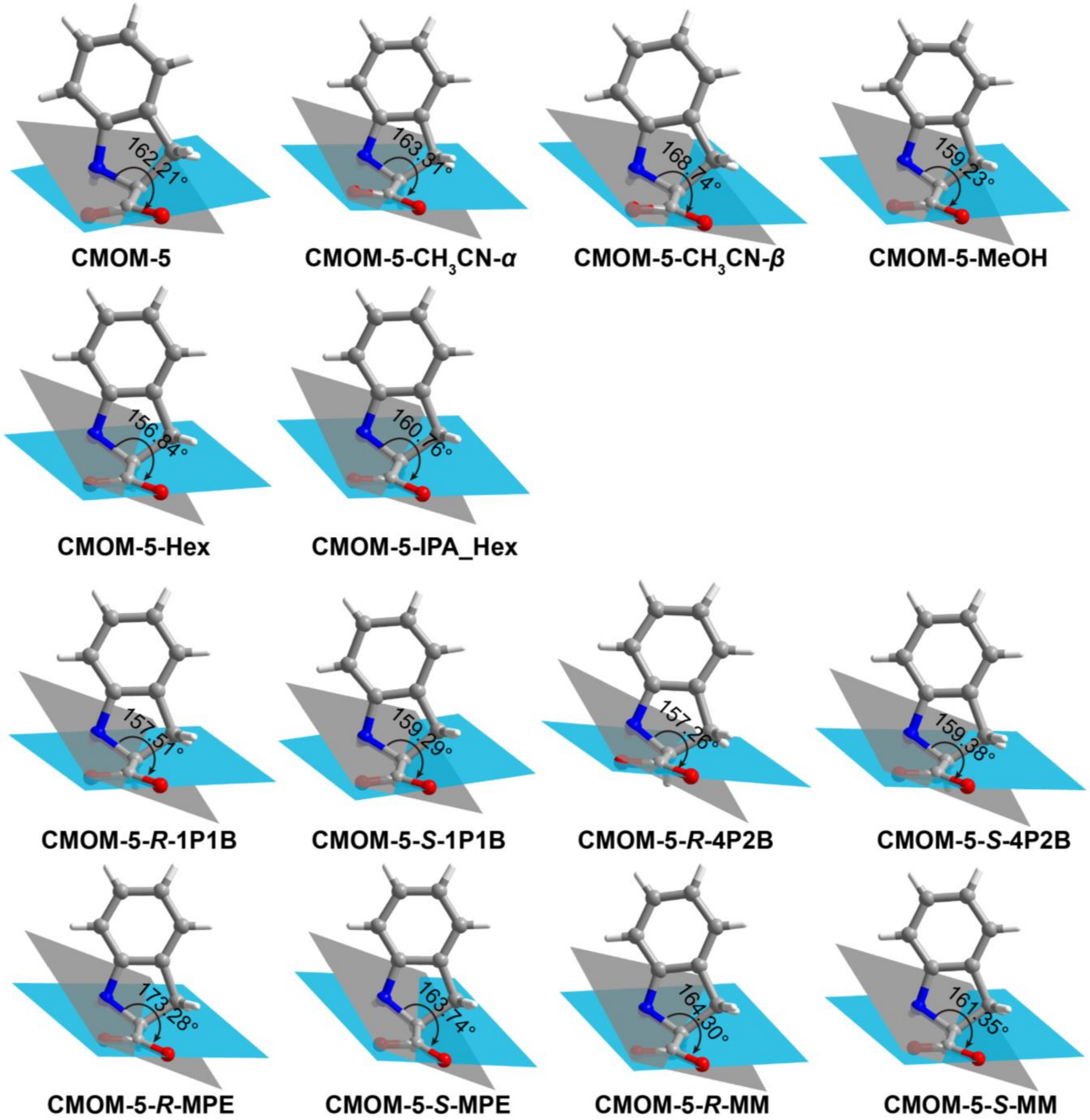

Figure S10. The torsion angles between the N-C bond and the C-O bond around the chiral carbon of *S*-IDEC in each structure.

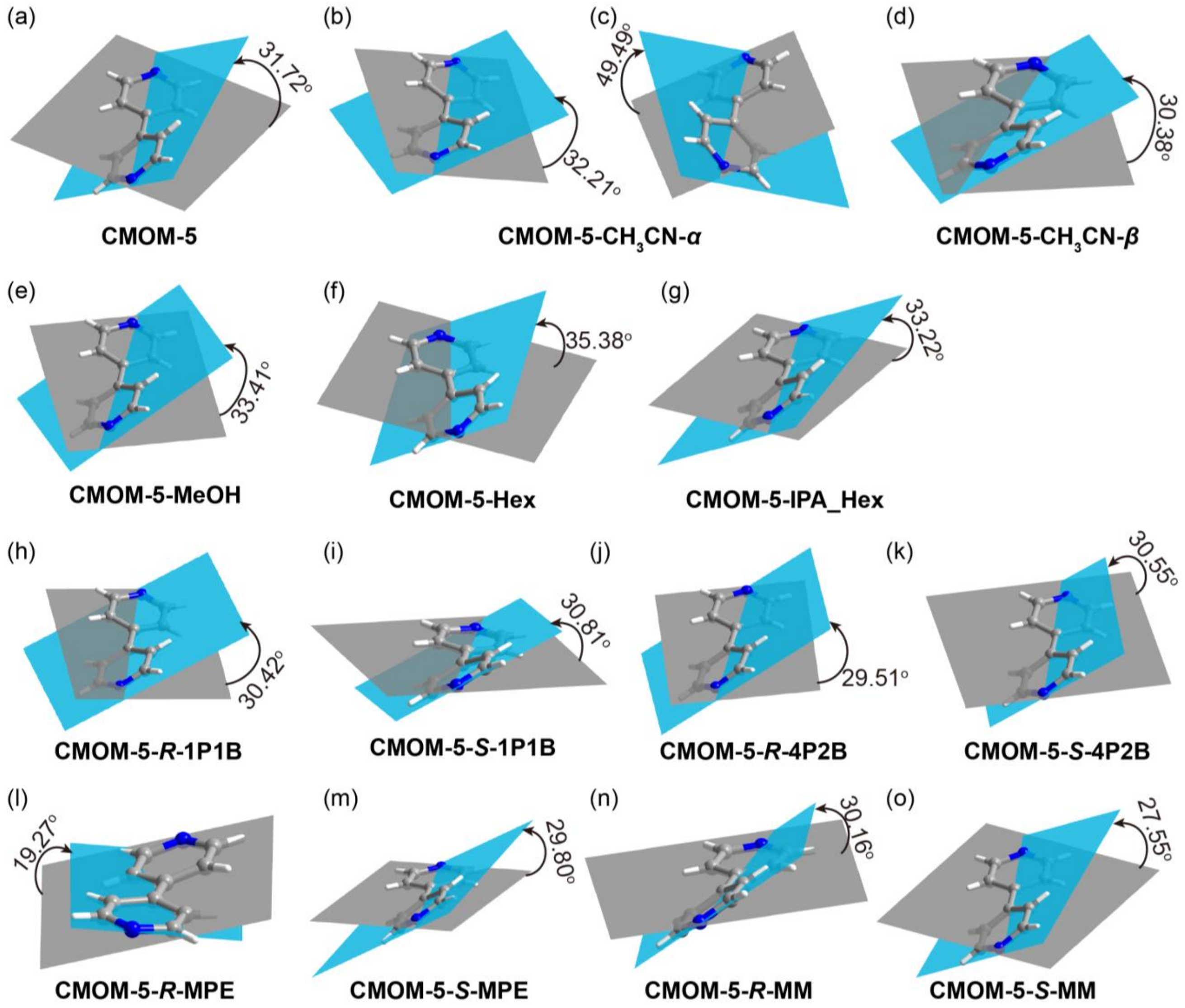

Figure S11. The dihedral angles between the pyridine planes of bipy coordinated in each structure. (b) and (c) are the dihedral angles in **CMOM-5-CH$_3$CN-*α*** corresponding to the disordered part 2 and part 1.

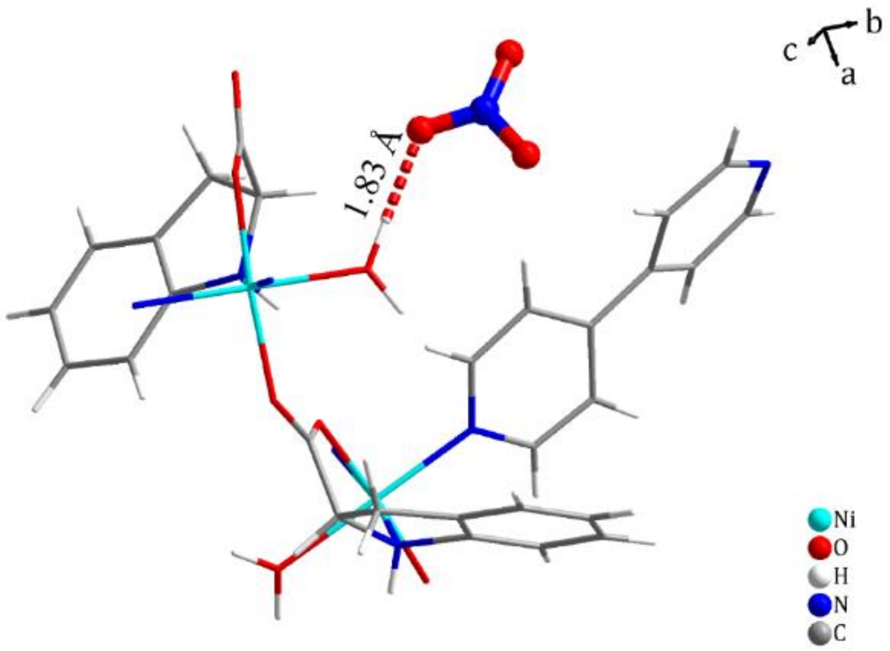

Figure S12. The hydrogen bond interaction between host framework and nitrate anion in **CMOM-5**. The hydrogen bond interactions were marked by red dotted lines.

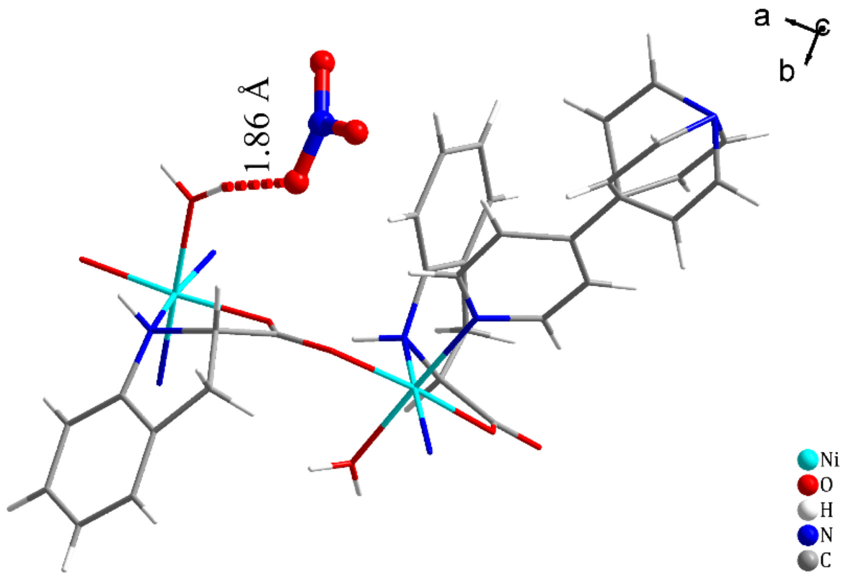

Figure S13. The hydrogen bond interactions between host framework and nitrate anion in **CMOM-5-CH_3CN-α**. The hydrogen bond interactions were marked by red dotted lines.

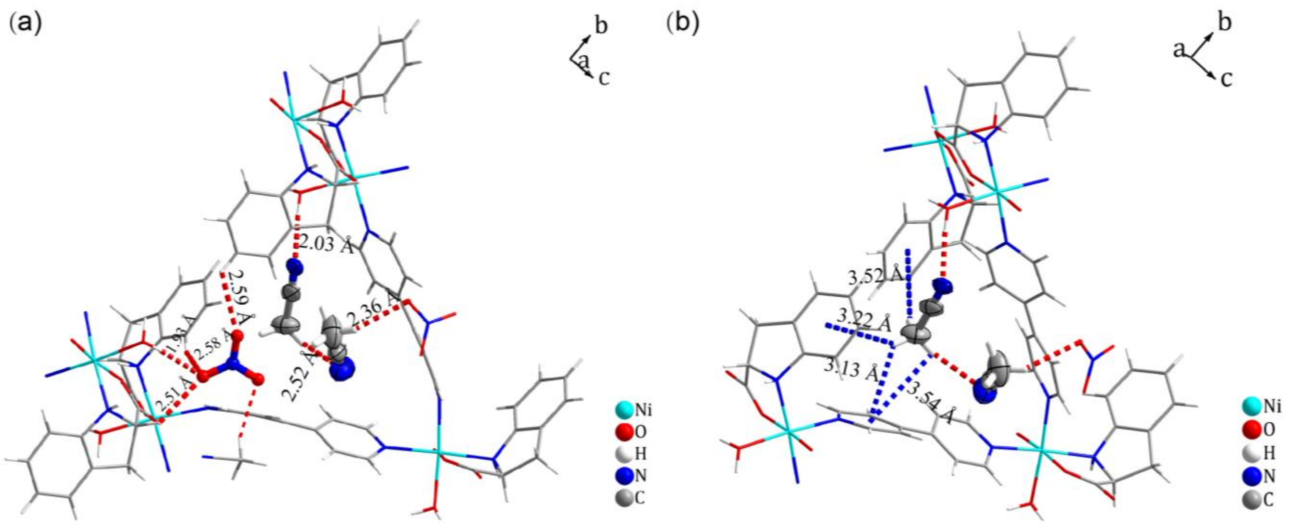

Figure S14. The structure of **CMOM-5-CH3CN-*β***. (a) The hydrogen bond interactions among host framework, nitrate anions and acetonitrile molecules. (b) the C-H···π interactions between the acetonitrile and the host framework. The hydrogen bond interactions were marked by red dotted lines, and the C-H···π interactions were marked by blue dotted lines. The non-hydrogen atoms of acetonitrile were drawn in thermal ellipsoids at 50% probability.

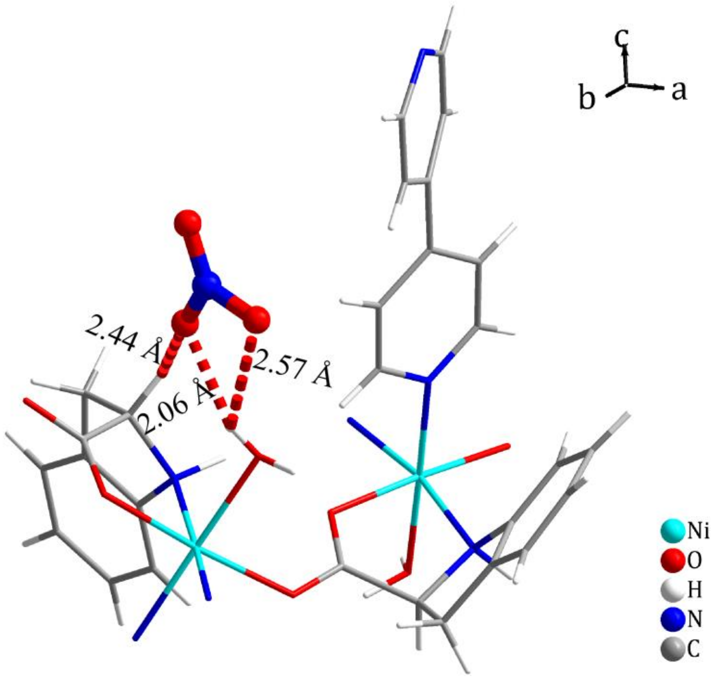

Figure S15. The hydrogen bond interaction between host framework and nitrate anion in **CMOM-5-MeOH**. The hydrogen bond interactions were marked by red dotted lines.

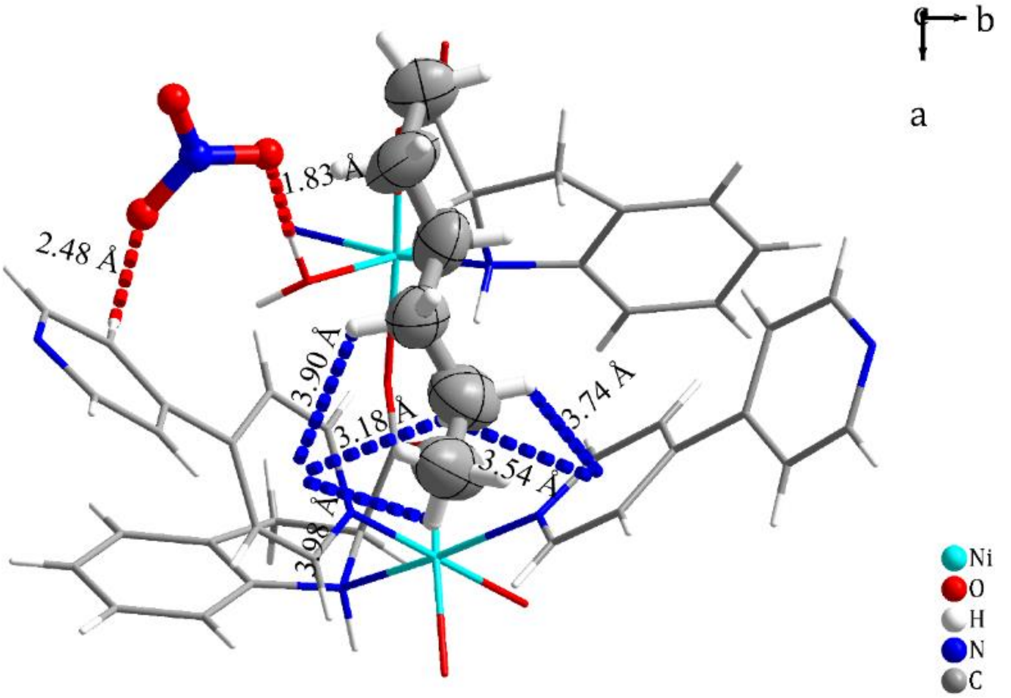

Figure S16. The intermolecular interactions among host framework, nitrate anion and hexane in **CMOM-5-Hex**. The hydrogen bond interactions were marked by red dotted lines and the C-H···π interaction was marked by blue dotted line.

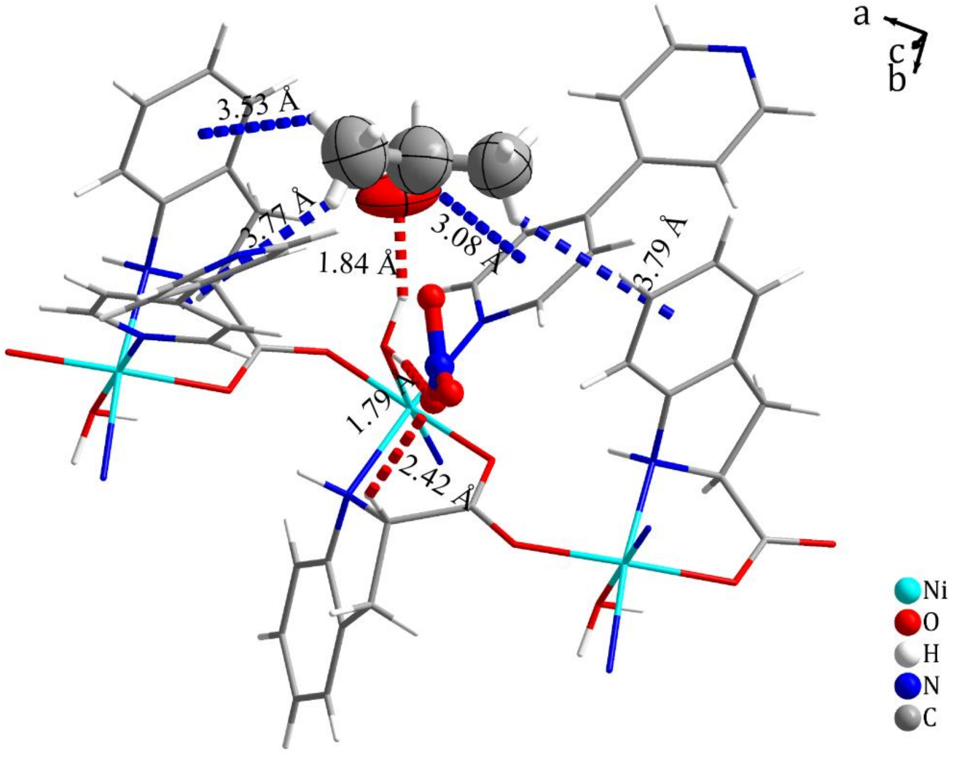

Figure S17. The hydrogen bond interactions among host framework, nitrate anion and IPA in **CMOM-5-IPA_Hex**. The hydrogen bond interactions were marked by red dotted lines, and the C-H···π interaction was marked by blue dotted line.

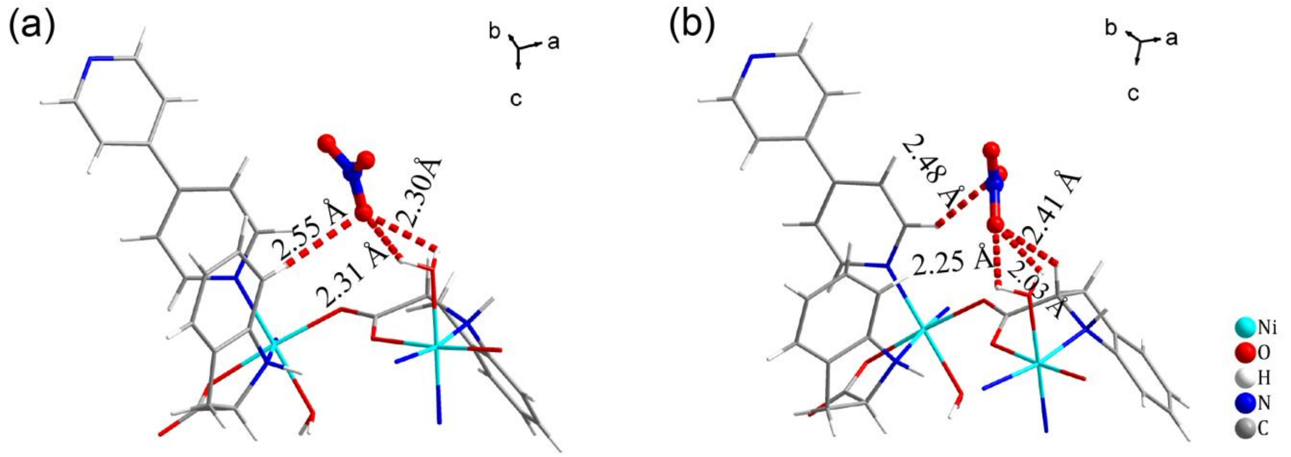

Figure S18. The hydrogen bond interactions between the host framework and the nitrate anion in **CMOM-5-*S*-1P1B**. (a) and (b) are corresponding to part 1 and part 2 of the disordered nitrate anion. The hydrogen bond interactions were marked by red dotted lines.

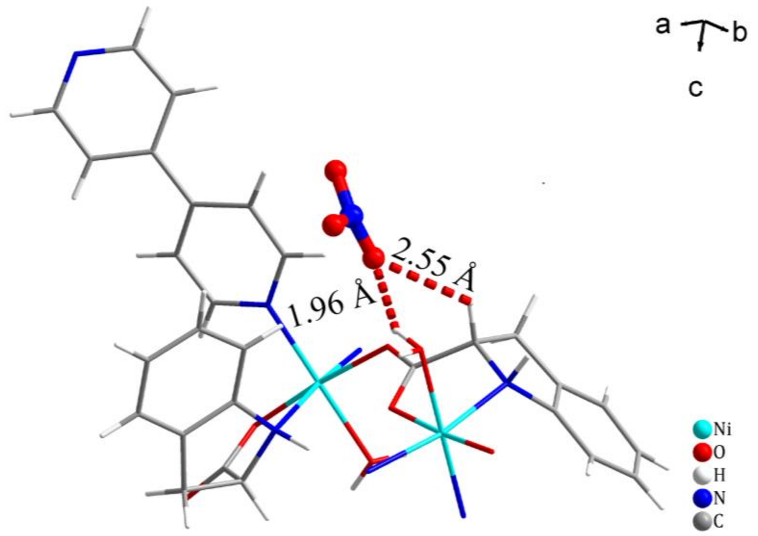

Figure S19. The hydrogen bond interactions between the host framework and counter anion in **CMOM-5-*S*-MPE**. The hydrogen bond interactions were marked by red dotted lines.

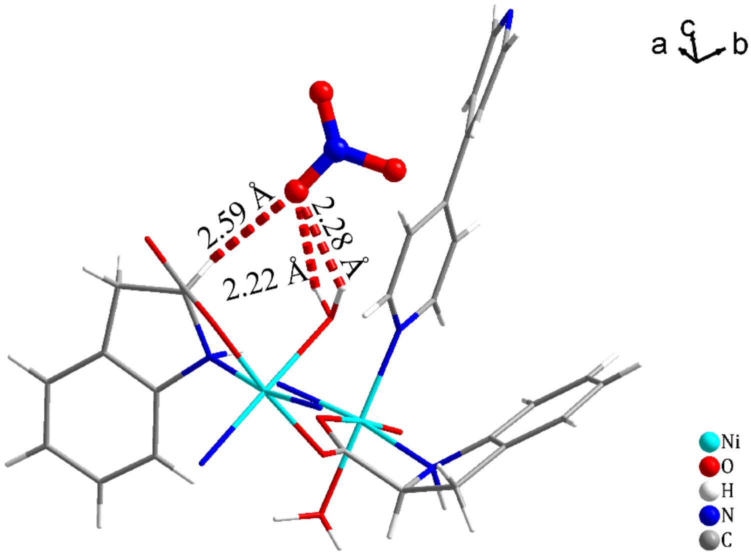

Figure S20. The hydrogen bond interactions between the host framework and counter anion in **CMOM-5-*R*-MM**. The hydrogen bond interactions were marked by red dotted lines.

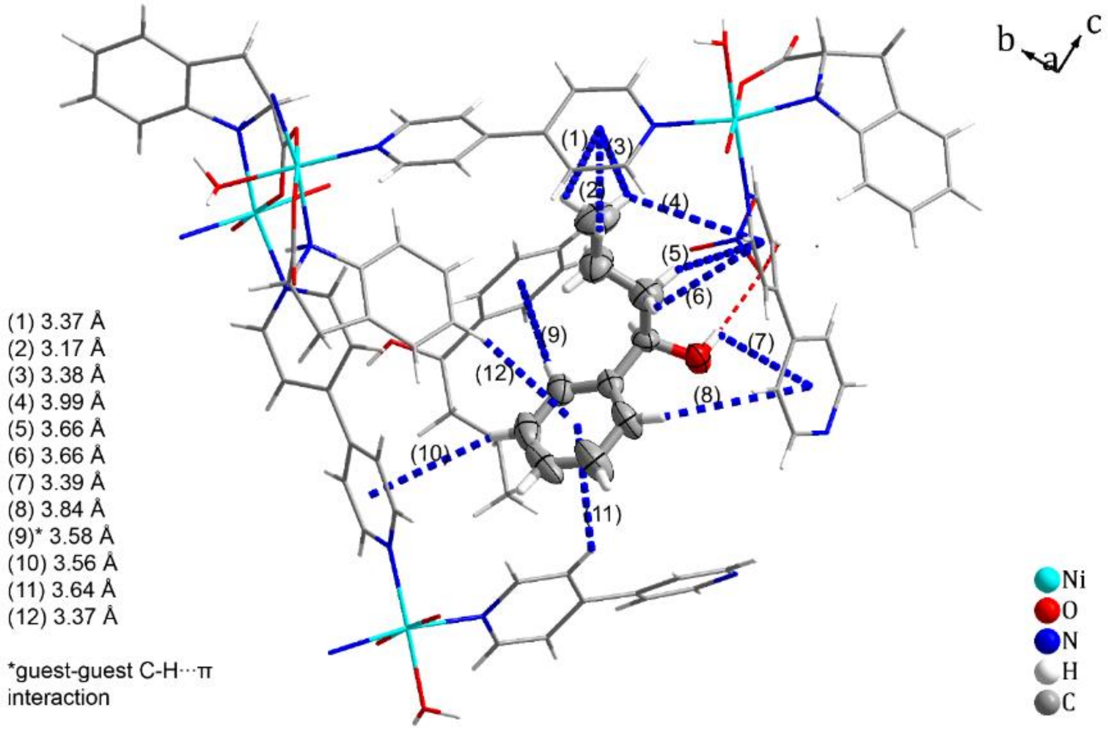

Figure S21. The intermolecular C-H···π interactions in **CMOM-5-*R*-1P1B**. The distance between hydrogen and the aromatic center was labelled. The hydrogen bond interactions were marked by red dotted lines, and the C-H···π interaction was marked by blue dotted line.

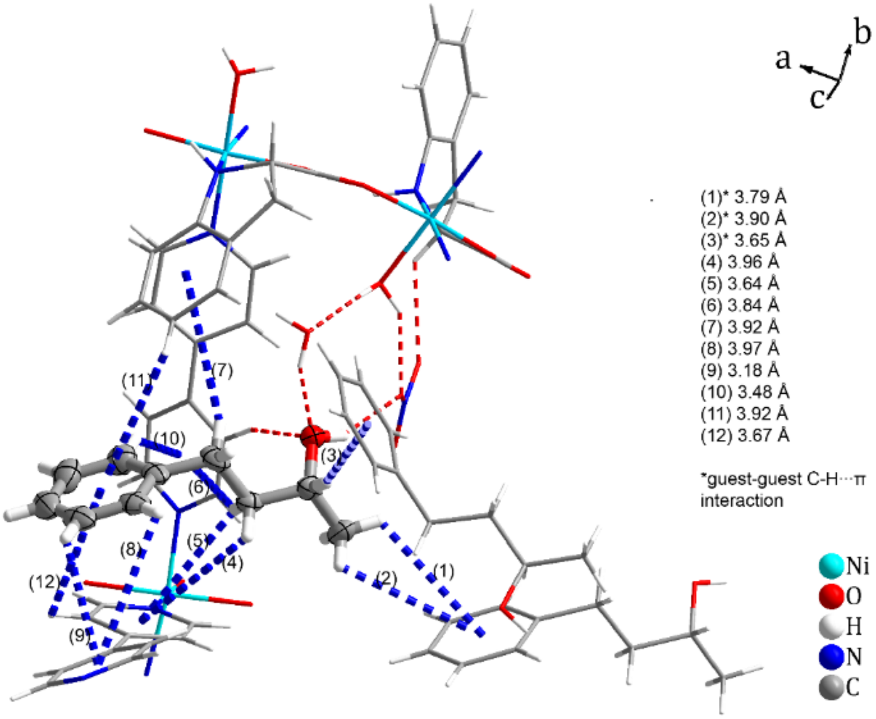

Figure S22. The intermolecular C-H···π interactions in **CMOM-5-*R*-4P2B**. The distance between hydrogen and the aromatic center was labelled. The hydrogen bond interactions were marked by red dotted lines, and the C-H···π interaction was marked by blue dotted line.

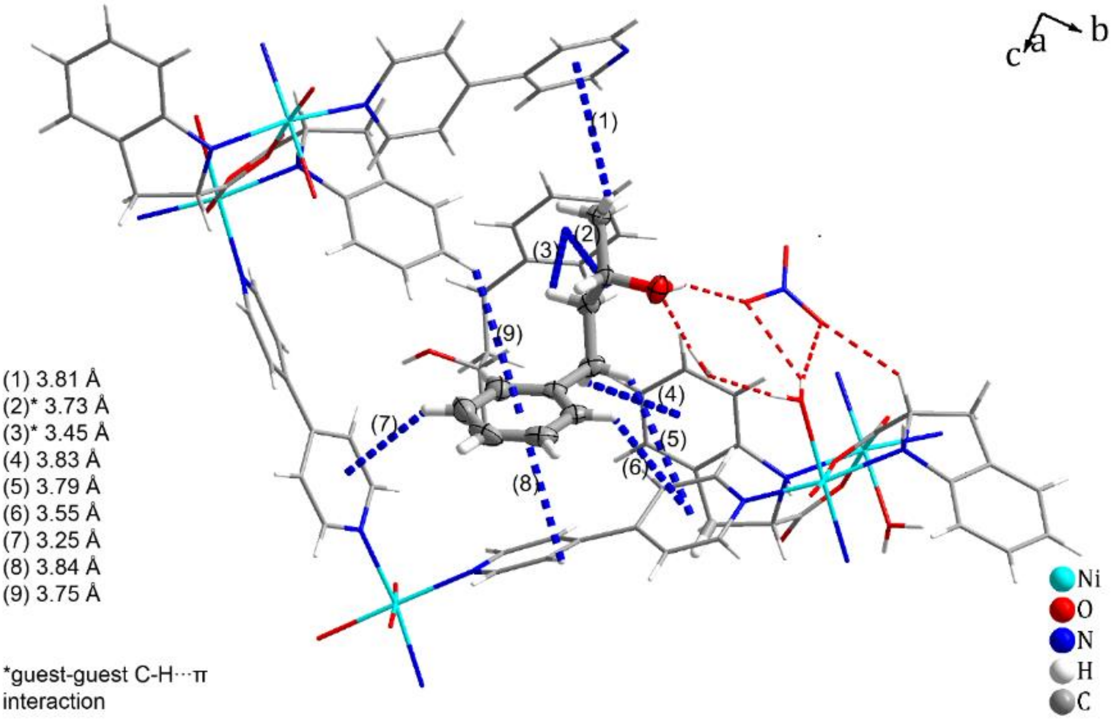

Figure S23. The intermolecular C-H···π interactions in **CMOM-5-*S*-4P2B**. The distance between hydrogen and the aromatic center was labelled. The hydrogen bond interactions were marked by red dotted lines, and the C-H···π interaction was marked by blue dotted line.

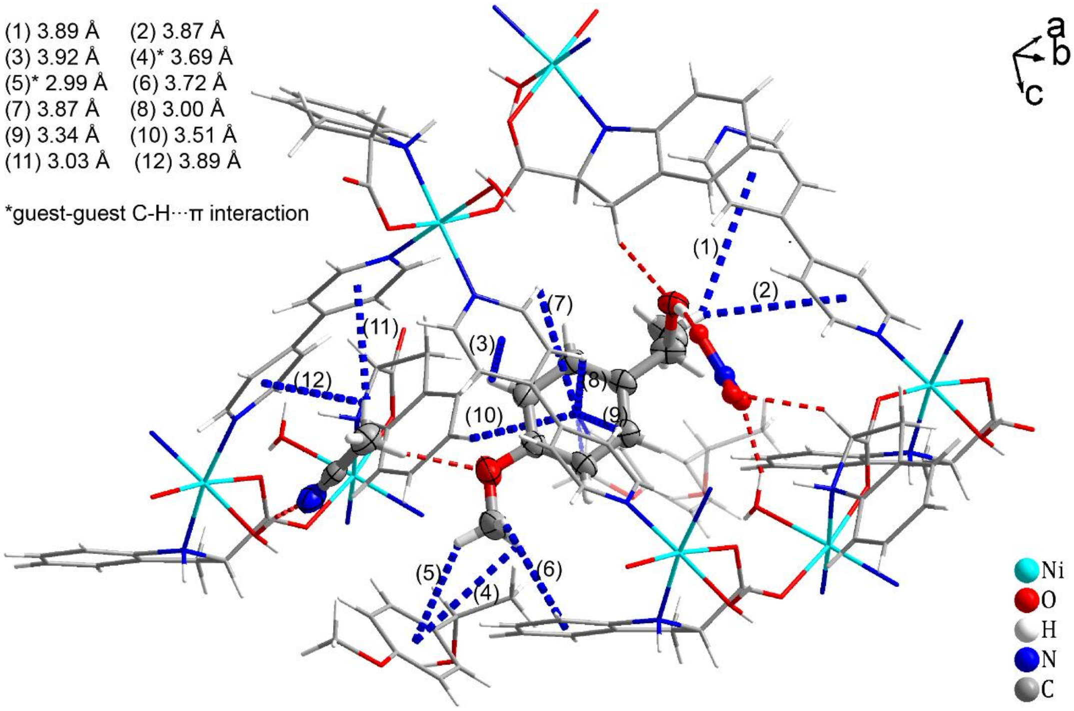

Figure S24. The intermolecular C-H···π interactions in **CMOM-5-*R*-MPE**. The distance between hydrogen and the aromatic center was labelled. The hydrogen bond interactions were marked by red dotted lines, and the C-H···π interaction was marked by blue dotted line.

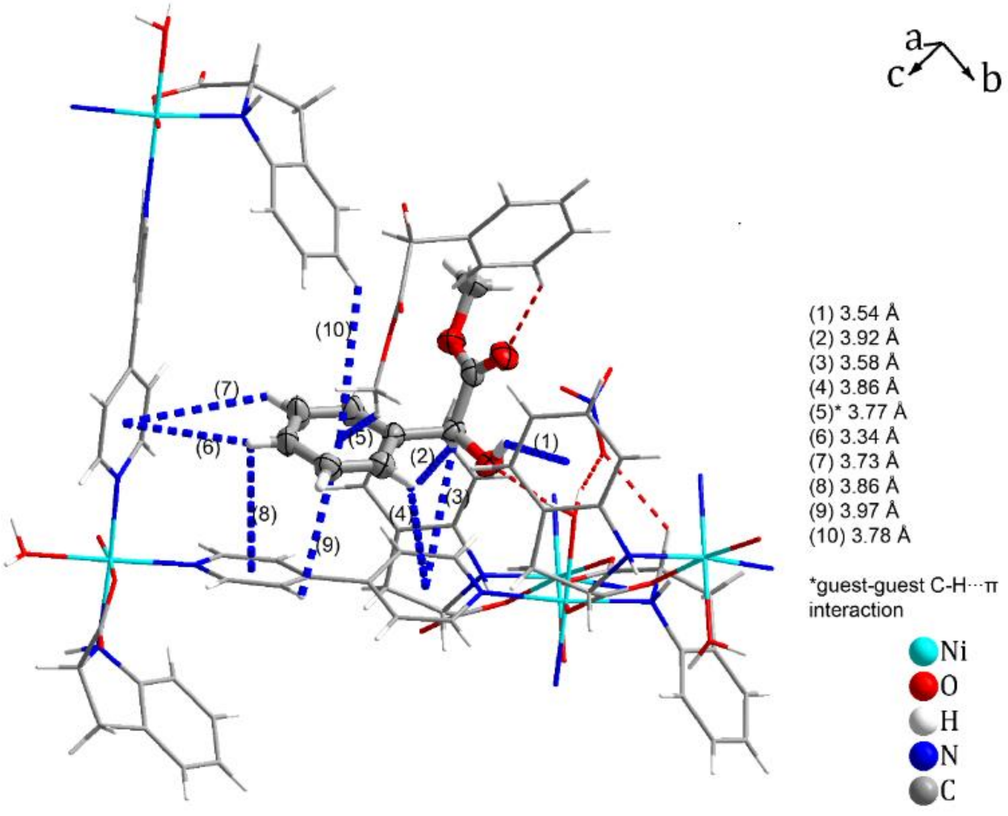

Figure S25. The intermolecular C-H···π interactions in **CMOM-5-*S*-MM**. The distance between hydrogen and the aromatic center was labelled. The hydrogen bond interactions were marked by red dotted lines, and the C-H···π interaction was marked by blue dotted line.

Table S13. Hydrogen bond parameters in **CMOM-5**.

**Hydrogen bond**	**Donor-H [Å]**	**Acceptor-H [Å]**	**Donor-Acceptor [Å]**	**Angle [°]**	**Symmetry operator**
N1-H1···O2*	0.85(5)	2.20(5)	2.938(6)	144(5)	1/2+x,1/2-y,1-z
O1-H1A···O5	0.95	1.83	2.761(11)	164	
C3-H3A···O3*	0.99	2.45	2.872(9)	105	
C10-H10···O3*	0.95	2.46	3.052(8)	120	1/2+x,1/2-y,1-z
C12-H12···O2*	0.95	2.35	2.988(9)	124	
C12-H12···O1*	0.95	2.51	3.367(8)	150	-1/2+x,1/2-y,1-z
C17-H17···O2*	0.95	2.56	3.057(9)	113	1-x,1/2+y,1/2-z

*The intra molecular hydrogen bond.

Table S14. Hydrogen bond parameters in **CMOM-5-CH_3CN-*α***.

**Hydrogen bond**	**Donor-H [Å]**	**Acceptor-H [Å]**	**Donor-Acceptor [Å]**	**Angle [°]**	**Symmetry operator**
N1-H1···O1*	1.00	2.18	2.907(11)	128	-1/2+x,3/2-y,1-z
O3-H3B···O4B	0.87(8)	1.86(8)	2.72(2)	166(7)	
C3-H3C···O2*	0.99	2.47	2.891(12)	105	
C10-H10···O1*	0.95	2.35	2.97(2)	123	
C10-H10···O3*	0.95	2.44	3.307(15)	152	1/2+x,3/2-y,1-z
C12-H12···O2*	0.95	2.46	3.042(18)	119	-1/2+x,3/2-y,1-z

*The intra molecular hydrogen bond.

Table S15. Hydrogen bond parameters in **CMOM-5-CH_3CN-*β***.

**Hydrogen bond**	**Donor-H [Å]**	**Acceptor-H [Å]**	**Donor-Acceptor [Å]**	**Angle [°]**	**Symmetry operator**
N1-H1···O3*	1.00	2.19	2.906(5)	127	1/2+x,1/2-y,1-z
O1-H1A···N5	0.90	2.03	2.928(8)	173	
O1-H1B···O4	0.90	1.93	2.797(6)	161	
C2-H2···O4	1.00	2.51	3.170(8)	124	1/2+x,1/2-y,1-z
C3-H3A···O2*	0.99	2.50	2.887(7)	103	
C6-H6···O5	0.95	2.59	3.238(9)	126	1/2+x,3/2-y,1-z
C8-H8···O4	0.95	2.58	3.464(8)	155	1+x,y,z
C10-H10···O2*	0.95	2.47	2.995(6)	115	
C12-H12···O3*	0.95	2.52	3.038(7)	114	-1/2+x,1/2-y,1-z
C17-H17···O1*	0.95	2.51	3.372(7)	152	1/2-x,1-y,1/2+z
C17-H17···O3*	0.95	2.33	2.944(7)	122	1/2-x,1-y,1/2+z
C19-H19···O2*	0.95	2.55	3.082(7)	116	-x,1/2+y,3/2-z
C21-H21C···N6	0.98	2.53	3.416(14)	151	1/2-x,1-y,1/2+z
C23-H23B···O6	0.98	2.36	3.237(16)	149	

*The intra molecular hydrogen bond.

Table S16. Hydrogen bond parameters in **CMOM-5-MeOH**.

Hydrogen bond	Donor-H [Å]	Acceptor-H [Å]	Donor-Acceptor [Å]	Angle [°]	Symmetry operator
N1-H1···O1*	1.00	2.19	2.930(7)	129	-1/2+x,3/2-y,1-z
O3-H3A···O4	0.88	2.06	2.830(19)	145	
O3-H3A···O5	0.88	2.57	3.241(14)	134	
C2-H2···O4	1.00	2.44	3.171(15)	129	
C3-H3C···O2*	0.99	2.35	2.818(10)	108	
C12-H12···O1*	0.95	2.54	3.037(12)	113	
C17-H17···O3*	0.95	2.40	3.232(12)	146	1/2-x,1-y,1/2+z
C17-H17···O1*	0.95	2.32	2.954(13)	124	1-x,-1/2+y,3/2-z
C19-H19···O2*	0.95	2.49	3.062(11)	119	3/2-x,1-y,1/2+z

*The intra molecular hydrogen bond.

Table S17. Hydrogen bond parameters in **CMOM-5-Hex**.

Hydrogen bond	Donor-H [Å]	Acceptor-H [Å]	Donor-Acceptor [Å]	Angle [°]	Symmetry operator
N1-H1···O2*	1.00	2.17	2.937(9)	132	1/2+x,3/2-y,1-z
O3-H3A···O4	0.89	1.83	2.678(9)	158	
O3-H3C···O1*	0.99	2.40	2.837(11)	106	
C10-H10···O1*	0.95	2.52	3.084(11)	118	
C12-H12···O2*	0.95	2.31	2.957(11)	125	-1/2+x,3/2-y,1-z
C12-H12···O3*	0.95	2.43	3.214(11)	139	-1/2+x,3/2-y,1-z
C17-H17···O2*	0.95	2.52	3.020(11)	113	1/2-x,1-y,1/2+z
C18-H18···O5	0.95	2.48	3.367(13)	156	-1/2+x,3/2-y,1-z
C19-H19···O1*	0.95	2.54	3.013(11)	111	-x,-1/2+y,3/2-z

*The intra molecular hydrogen bond.

Table S18. Hydrogen bond parameters in **CMOM-5-IPA_Hex**.

Hydrogen bond	Donor-H [Å]	Acceptor-H [Å]	Donor-Acceptor [Å]	Angle [°]	Symmetry operator
N1-H1···O2*	1.00	2.17	2.914(11)	131	1/2+x,3/2-y,1-z
O3-H3A···O7	0.96	1.84	2.748(16)	156	
O3-H3B···O4	0.96	1.79	2.745(19)	172	
C2-H2···O4	1.00	2.42	3.137(19)	128	1/2+x,3/2-y,1-z
C3-H3C···O1*	0.99	2.40	2.845(17)	106	
C10-H10···O1*	0.95	2.50	3.010(14)	114	
C17-H17···O2*	0.95	2.31	2.951(13)	125	1/2-x,1-y,-1/2+z
C17-H17···O3*	0.95	2.52	3.357(13)	147	1/2-x,1-y,-1/2+z
C19-H19···O1*	0.95	2.49	3.069(14)	120	-x,-1/2+y,1/2-z

*The intra molecular hydrogen bond.

Table S19. Hydrogen bond parameters in **CMOM-5-*R*-1P1B**.

Hydrogen bond	Donor-H [Å]	Acceptor-H [Å]	Donor-Acceptor [Å]	Angle [°]	Symmetry operator
N1-H1···O1*	1.00	2.31	3.015(9)	126	1/2+x,1/2-y,1-z
O3-H3A···O1*	0.94(8)	2.54(10)	2.889(8)	102(8)	
O3-H3A···O4	0.94(8)	1.84(9)	2.745(10)	160(8)	
O7-H7A···O5	0.84	2.01	2.843(11)	174	3/2-x,1-y,1/2+z
C3-H3C···O2*	0.99	2.44	2.869(12)	106	
C6-H6···O4	0.95	2.45	3.288(13)	147	1/2+x,1/2-y,1-z
C9-H9···O6	0.95	2.50	3.418(14)	162	1-x,-1/2+y,1/2-z
C10-H10···O1*	0.95	2.53	3.034(12)	113	
C12-H12···O2*	0.95	2.57	3.023(11)	109	1/2+x,1/2-y,1-z
C17-H17···O2*	0.95	2.51	3.083(10)	119	1/2-x,1-y,1/2+z
C17-H17···O6	0.95	2.54	3.287(13)	136	1/2-x,1-y,1/2+z
C18-H18···O5	0.95	2.54	3.402(13)	152	3/2-x,1-y,1/2+z
C19-H19···O3*	0.95	2.46	3.248(11)	140	3/2-x,1-y,1/2+z
C19-H19···O1*	0.95	2.30	2.947(10)	125	1-x,1/2+y,3/2-z

*The intra molecular hydrogen bond.

Table S20. Hydrogen bond parameters in **CMOM-5-*S*-1P1B**.

Hydrogen bond	Donor-H [Å]	Acceptor-H [Å]	Donor-Acceptor [Å]	Angle [°]	Symmetry operator
N1-H1···O1*	1.00	2.18	2.919(6)	129	1/2+x,3/2-y,1-z
O3-H3A···O4A	0.91	2.30	2.756(14)	111	
O3-H3A···O4B	0.91	2.25	2.59(4)	101	
O3-H3B···O4A	0.91	2.31	2.756(14)	110	
O3-H3B···O4B	0.91	2.03	2.59(4)	118	
C2-H2···O4B	1.00	2.41	3.18(6)	132	
C3-H3C···O2*	0.99	2.43	2.856(9)	106	
C6-H6···O4A	0.95	2.55	3.429(17)	153	1/2+x,3/2-y,1-z
C10-H10···O2*	0.95	2.56	3.050(9)	112	1/2+x,3/2-y,1-z
C12-H12···O1*	0.95	2.54	3.024(10)	112	
C17-H17···O3*	0.95	2.44	3.266(8)	145	3/2-x,1-y,-1/2+z
C17-H17···O1*	0.95	2.31	2.949(9)	124	1-x,-1/2+y,1/2-z
C19-H19···O2*	0.95	2.51	3.081(8)	119	1/2-x,1-y,-1/2+z
C19-H19···O5B	0.95	2.48	3.28(4)	142	1/2-x,1-y,-1/2+z

*The intra molecular hydrogen bond.

Table S21. Hydrogen bond parameters in **CMOM-5-*R*-4P2B**.

Hydrogen bond	Donor-H [Å]	Acceptor-H [Å]	Donor-Acceptor [Å]	Angle [°]	Symmetry operator
N1-H1···O2*	1.00	2.29	3.017(2)	129	-1/2+x,3/2-y,1-z
O3-H3A···O8	0.87	1.85	2.686(3)	160	
O3-H3B···O4	0.87	2.07	2.830(4)	145	
O7-H7A···O4	0.84	1.95	2.724(4)	152	
O8-H8B···O7	0.87	1.92	2.727(4)	153	
C2-H2···O6	1.00	2.51	3.280(4)	134	-1/2+x,3/2-y,1-z
C3-H3C···O1*	0.99	2.42	2.856(3)	106	
C10-H10···O1*	0.95	2.50	2.987(3)	112	
C11-H11···O7	0.95	2.45	3.347(4)	158	3/2-x,1-y,1/2+z
C12-H12···O2*	0.95	2.55	3.055(3)	113	1/2+x,3/2-y,1-z
C17-H17···O6	0.95	2.43	3.238(4)	143	5/2-x,1-y,1/2+z
C17-H17···O1*	0.95	2.46	3.053(3)	120	2-x,-1/2+y,3/2-z
C19-H19···O2*	0.95	2.34	2.973(3)	124	3/2-x,1-y,1/2+z
C19-H19···O3*	0.95	2.57	3.386(3)	144	3/2-x,1-y,1/2+z
C23-H23B···O7*	0.99	2.53	2.888(5)	101	

*The intra molecular hydrogen bond.

Table S22. Hydrogen bond parameters in **CMOM-5-*S*-4P2B**.

Hydrogen bond	Donor-H [Å]	Acceptor-H [Å]	Donor-Acceptor [Å]	Angle [°]	Symmetry operator
N1-H1···O1*	1.00	2.22	2.952(4)	129	1/2+x,3/2-y,1-z
O3-H3A···O4	0.89(3)	2.01(3)	2.869(5)	162(3)	
O3-H3A···O6	0.89(3)	2.49(5)	3.110(7)	128(4)	
O3-H3B···O8	0.90(4)	1.80(4)	2.700(4)	172(4)	
O7-H7A···O6	0.84	1.92	2.662(8)	146	
O8-H8A···O7	0.87	1.89	2.759(5)	174	
C2-H2···O4	1.00	2.54	3.244(5)	128	
C3-H3C···O2*	0.99	2.41	2.848(4)	106	
C10-H10···O2*	0.95	2.54	2.989(4)	109	1/2+x,3/2-y,1-z
C12-H12···O1*	0.95	2.57	3.077(4)	114	
C17-H17···O3*	0.95	2.50	3.309(5)	143	3/2-x,1-y,-1/2+z
C17-H17···O1*	0.95	2.34	2.966(4)	123	1-x,-1/2+y,1/2-z
C18-H18···O2*	0.95	2.47	3.053(4)	119	1/2-x,1-y,-1/2+z

*The intra molecular hydrogen bond.

Table S23. Hydrogen bond parameters in **CMOM-5-*R*-MPE**.

**Hydrogen bond**	**Donor-H [Å]**	**Acceptor-H [Å]**	**Donor-Acceptor [Å]**	**Angle [°]**	**Symmetry operator**
N1-H1···O2*	1.00	2.30	2.991(11)	125	1/2+x,1/2-y,1-z
O3-H3A···O4	0.87	1.94	2.759(14)	155	
O3-H3B···N5	0.87	2.11	2.911(17)	153	
O7-H7A···O5	0.84	2.01	2.839(18)	170	
C2-H2···O6	1.00	2.40	3.257(18)	144	1/2+x,1/2-y,1-z
C3-H3D···O7	0.99	2.41	3.284(19)	147	1/2-x,1-y,1/2+z
C10-H10···O1*	0.95	2.42	3.021(16)	121	
C12-H12···O2*	0.95	2.50	3.047(16)	117	-1/2+x,1/2-y,1-z
C17-H17···O2*	0.95	2.46	3.063(17)	121	1/2-x,1-y,1/2+z
C19-H19···O1*	0.95	2.41	3.014(18)	121	-x,1/2+y,3/2-z
C30-H30A···O8	0.98	2.30	3.25(2)	162	-1/2+x,3/2-y,1-z

*The intra molecular hydrogen bond.

Table S24. Hydrogen bond parameters in **CMOM-5-*S*-MPE**.

**Hydrogen bond**	**Donor-H [Å]**	**Acceptor-H [Å]**	**Donor-Acceptor [Å]**	**Angle [°]**	**Symmetry operator**
N1-H1···O2*	1.00	2.19	2.929(5)	130	1/2+x,3/2-y,1-z
O3-H3B···O4	0.87	1.96	2.749(10)	151	
C2-H2···O4	1.00	2.55	3.249(11)	127	1/2+x,3/2-y,1-z
C3-H3C···O1*	0.99	2.45	2.867(7)	104	
C10-H10···O1*	0.95	2.56	3.032(7)	111	
C12-H12···O2*	0.95	2.54	3.043(7)	113	-1/2+x,3/2-y,1-z
C17-H17···O2*	0.95	2.35	2.980(7)	123	1/2-x,1-y,-1/2+z
C17-H17···O3*	0.95	2.49	3.330(7)	147	1/2-x,1-y,-1/2+z
C19-H19···O1*	0.95	2.51	3.088(7)	120	-x,-1/2+y,1/2-z

*The intra molecular hydrogen bond.

Table S25. Hydrogen bond parameters in **CMOM-5-*R*-MM**.

**Hydrogen bond**	**Donor-H [Å]**	**Acceptor-H [Å]**	**Donor-Acceptor [Å]**	**Angle [°]**	**Symmetry operator**
N1-H1···O1*	1.00	2.25	2.967(7)	127	-1/2+x,1/2-y,1-z
O3-H3A···O4	0.98	2.28	2.742(14)	108	
O3-H3B···O4	0.98	2.22	2.742(14)	113	
C2-H2···O4	1.00	2.59	3.290(15)	127	
C3-H3C···O2*	0.99	2.47	2.870(11)	104	
C10-H10···O2*	0.95	2.59	3.019(10)	107	-1/2+x,1/2-y,1-z
C17-H17···O2*	0.95	2.46	3.053(10)	120	3/2-x,1-y,-1/2+z
C19-H19···O3*	0.95	2.49	3.344(10)	149	1/2-x,1-y,-1/2+z
C19-H19···O1*	0.95	2.35	2.989(10)	125	1-x,1/2+y,1/2-z

*The intra molecular hydrogen bond.

Table S26. Hydrogen bond parameters in **CMOM-5-*S*-MM**.

Hydrogen bond	Donor-H [Å]	Acceptor-H [Å]	Donor-Acceptor [Å]	Angle [°]	Symmetry operator
N1-H1···O2*	1.00	2.21	2.939(7)	128	-1/2+x,3/2-y,1-z
O3-H3A···O4	0.86(6)	1.90(6)	2.734(8)	165(7)	
O3-H3A···O7	0.90(7)	1.92(7)	2.809(9)	172(8)	
O7-H7A···O8*	0.84	2.18	2.653(9)	115	
C2-H2···O4	1.00	2.51	3.172(10)	123	-1/2+x,3/2-y,1-z
C3-H3C···O1*	0.99	2.43	2.860(10)	106	
C10-H10···O1*	0.95	2.55	3.012(9)	110	
C12-H12···O2*	0.95	2.53	3.031(9)	113	1/2+x,3/2-y,1-z
C17-H17···O2*	0.95	2.31	2.957(9)	125	3/2-x,1-y,1/2+z
C17-H17···O3*	0.95	2.41	3.247(9)	146	3/2-x,1-y,1/2+z
C19-H19···O1*	0.95	2.50	3.071(9)	118	2-x,-1/2+y,3/2-z
C24-H24···O7*	0.95	2.43	2.789(12)	102	
C27-H27···O8	0.95	2.47	3.264(13)	141	1/2+x,3/2-y,1-z
C28-H28···O9*	0.95	2.57	3.054(11)	112	

*The intra molecular hydrogen bond.

Thermogravimetric Analysis

Thermogravimetric analyses (TGA) were performed under N_2 (60 mL per minute) using a TA Instruments Q50 system. Samples were loaded into aluminium sample pans and heated at 10 K min^{-1} from room temperature to 550 °C.

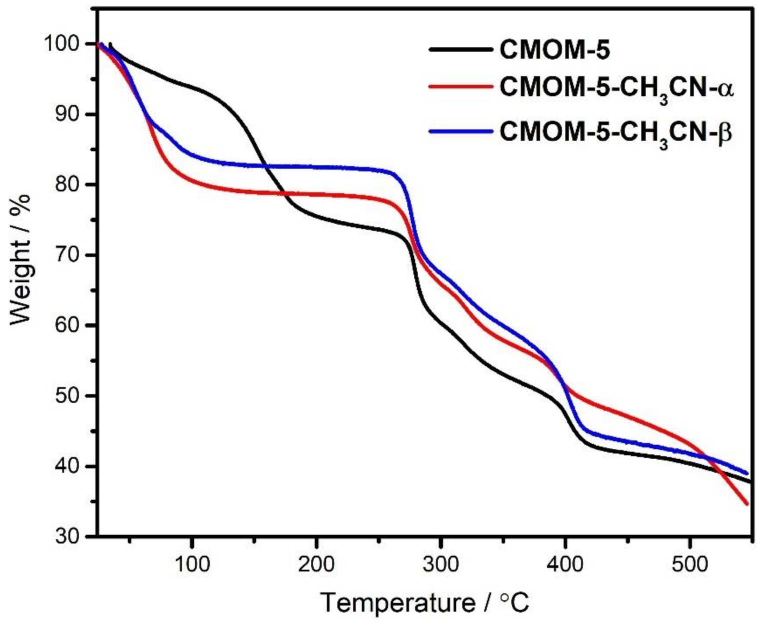

Figure S26. The TGA curves of **CMOM-5**, **CMOM-5-CH_3CN-*α*** and **CMOM-5-CH_3CN-*β***.

Powder X-ray Diffusion Analysis

Powder X-ray Diffusion (PXRD) data was collected at the room temperature on a PANalytical Empyream (Cu Kα, λ = 1.5418 Å) with a 1D PIXcel strip detector. Experiments were conducted in continuous scanning mode with the goniometer in the theta-theta orientation. Incident beam optics included the Fixed Divergences slit with anti-scatter slit PreFIX module, with a 1/8° divergence slit and a 1/4° anti-scatter slit, as well as a 10 mm fixed incident beam mask and a Soller slit (0.04 rad). Divergent beam optics included a P7.5 anti-scatter slit, a Soller slit (0.04 rad), and a Ni *β* filter. The data were collected in the range of 2θ = 3-50°. Raw data was then evaluated using the X'Pert HighScore Plus™ software V4.1 (PANalytical, The Netherlands).

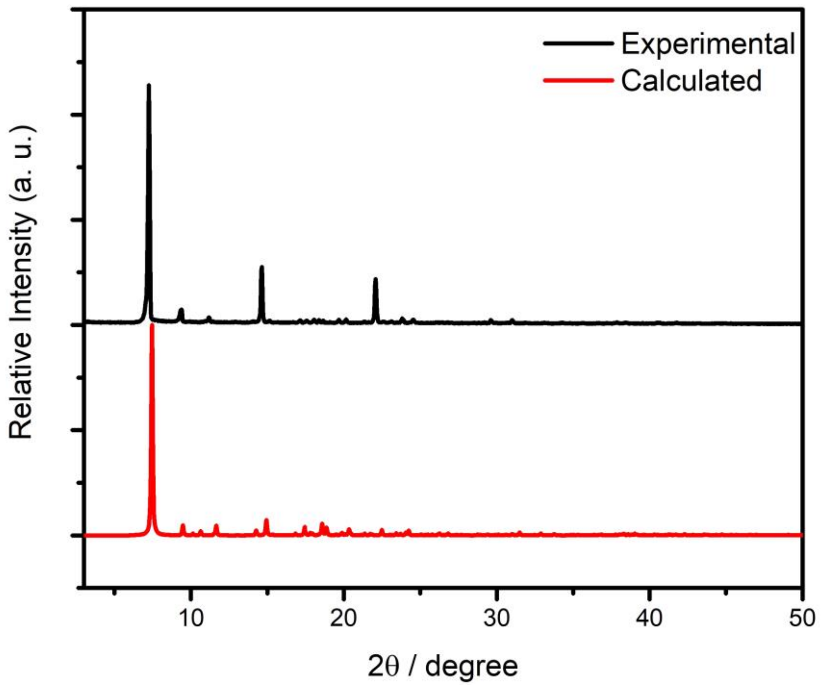

Figure S27. The PXRD patterns of **CMOM-5-MeOH**.

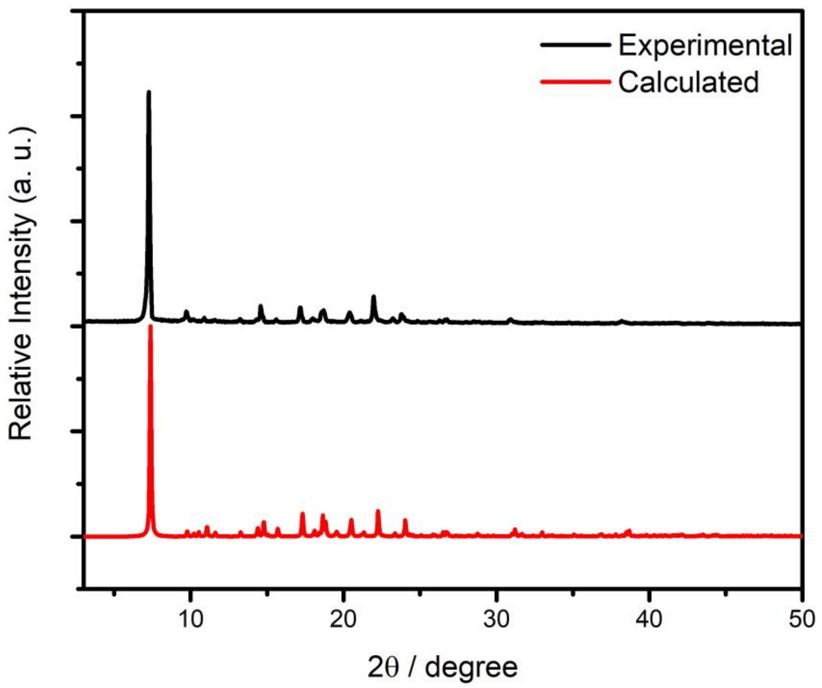

Figure S28. The PXRD patterns of **CMOM-5-Hex**.

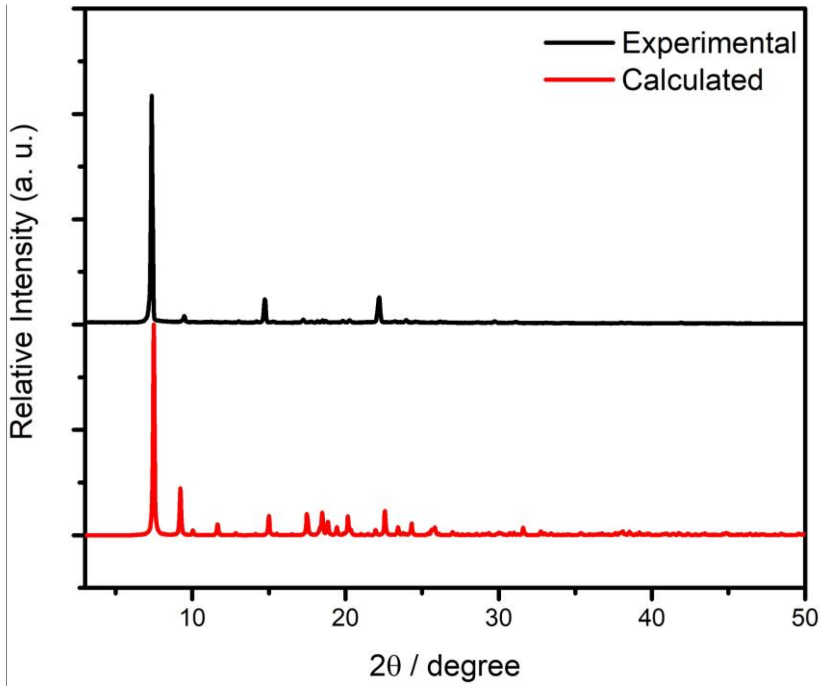

Figure S29. The PXRD patterns of **CMOM-5-IPA_Hex**.

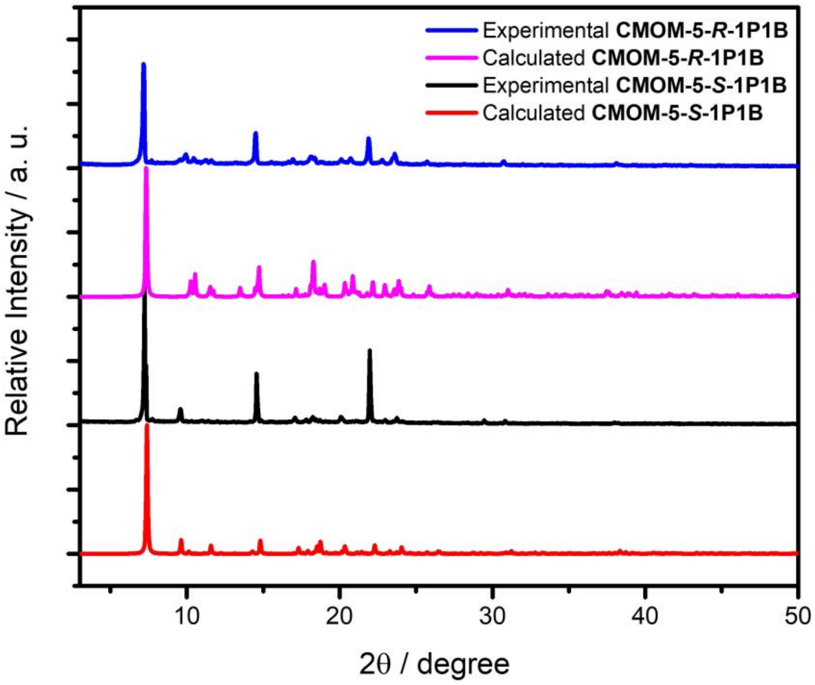

Figure S30. The PXRD patterns of **CMOM-5-*R*-1P1B** and **CMOM-5-*S*-1P1B**.

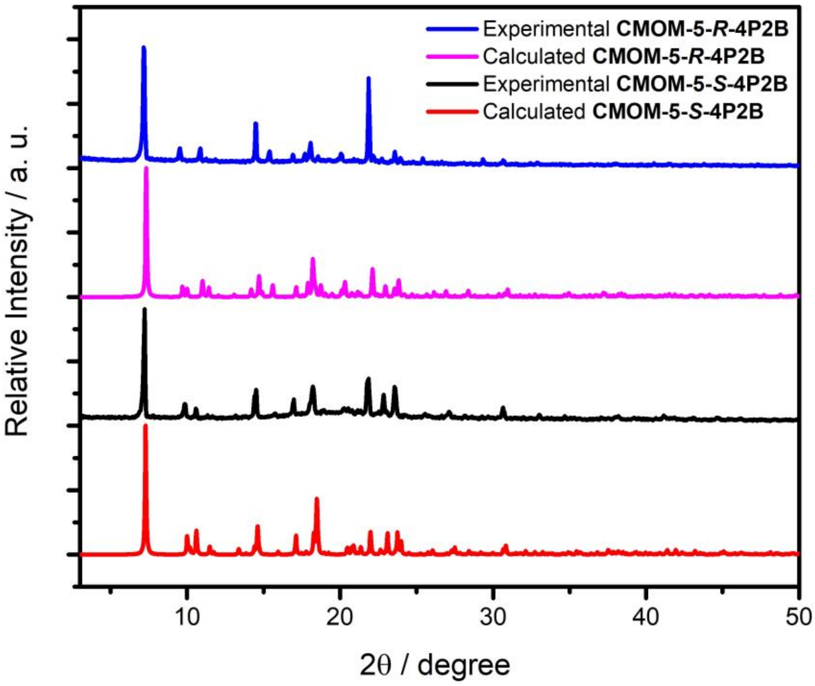

Figure S31. The PXRD patterns of **CMOM-5-*R*-4P2B** and **CMOM-5-*S*-4P2B**.

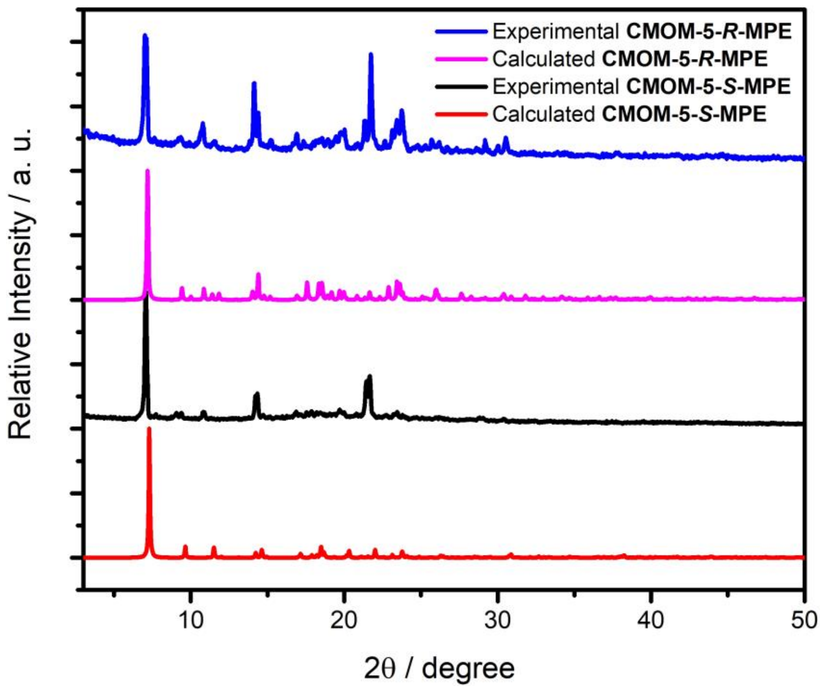

Figure S32. The PXRD patterns of **CMOM-5-*R*-MPE** and **CMOM-5-*S*-MPE**.

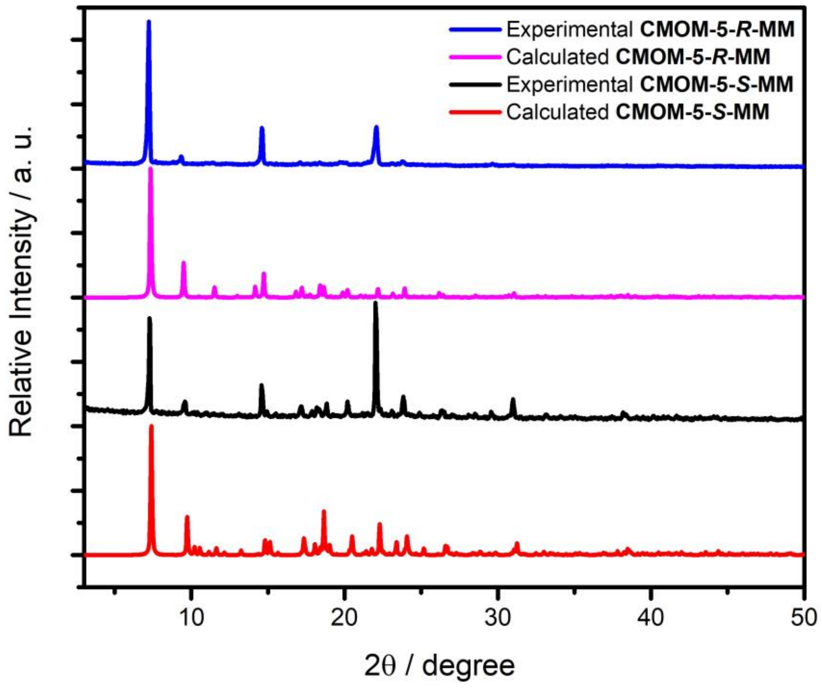

Figure S33. The PXRD patterns of **CMOM-5-*R*-MM** and **CMOM-5-*S*-MM**.

NMR Spectra

The NMR spectra were recorded on a JEOL ECX400 NMR spectrometer. Crystal sample (about 4 mg) with chiral molecules loaded were fileted from the solution. When the toluene was volatilized away, dissolving the sample in a mixture of 3 mL deuterium chloride (1M solution in D_2O) and 3 mL D_6-dimethyl sulfoxide (d_6-DMSO) in an NMR tube. The chemical shift of d_6-DMSO was labelled at 2.50 ppm for all data. The multiple peaks around 3.86 ppm was attributed to deuterium chloride.[33]

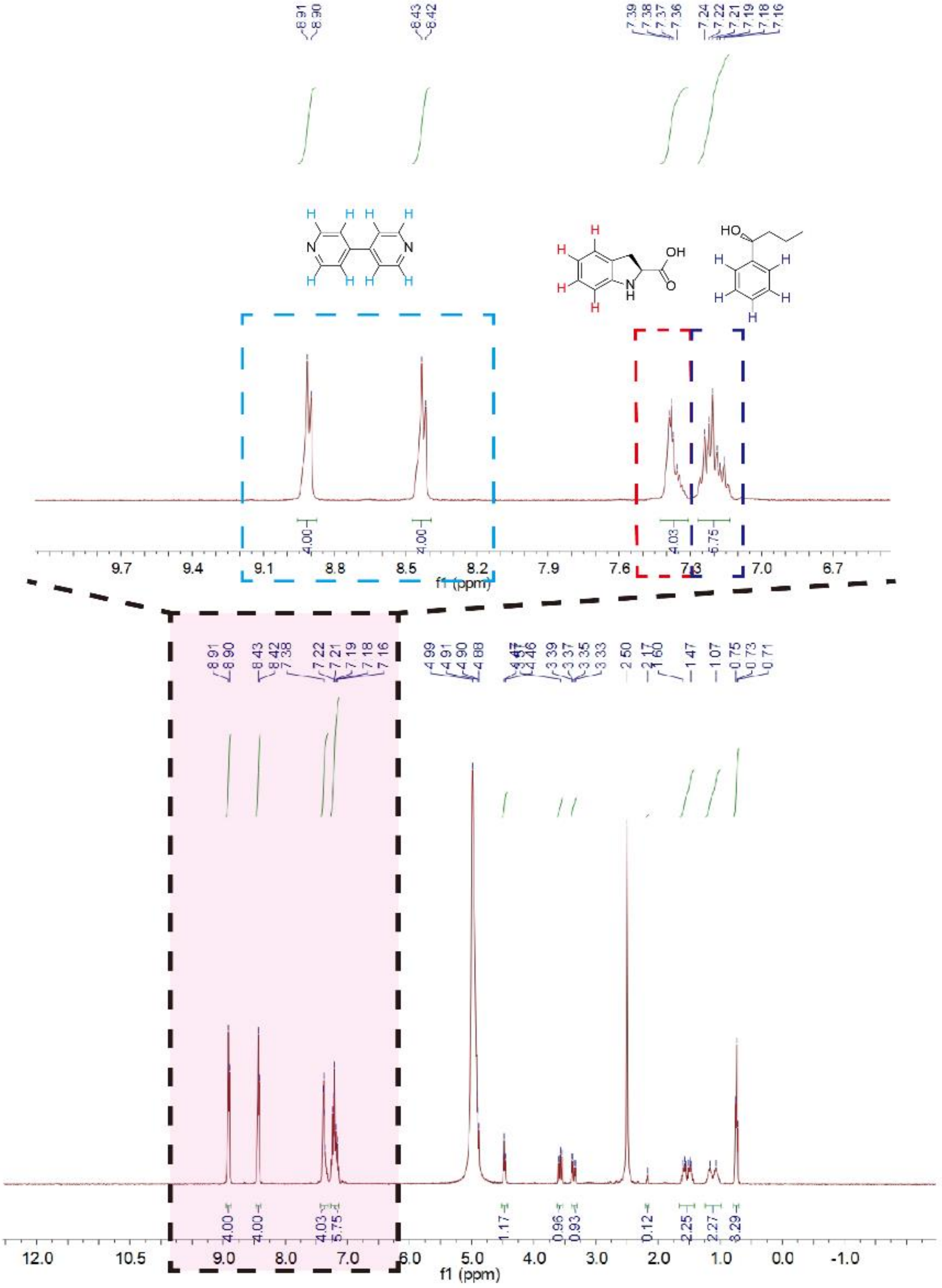

Figure S34. ^{1}H NMR spectrum of digested **CMOM-5-*R*-1P1B**. The ratio among bipy: *S*-IDEC: *R*-1P1B was solved to be 1:1.01:1.15.

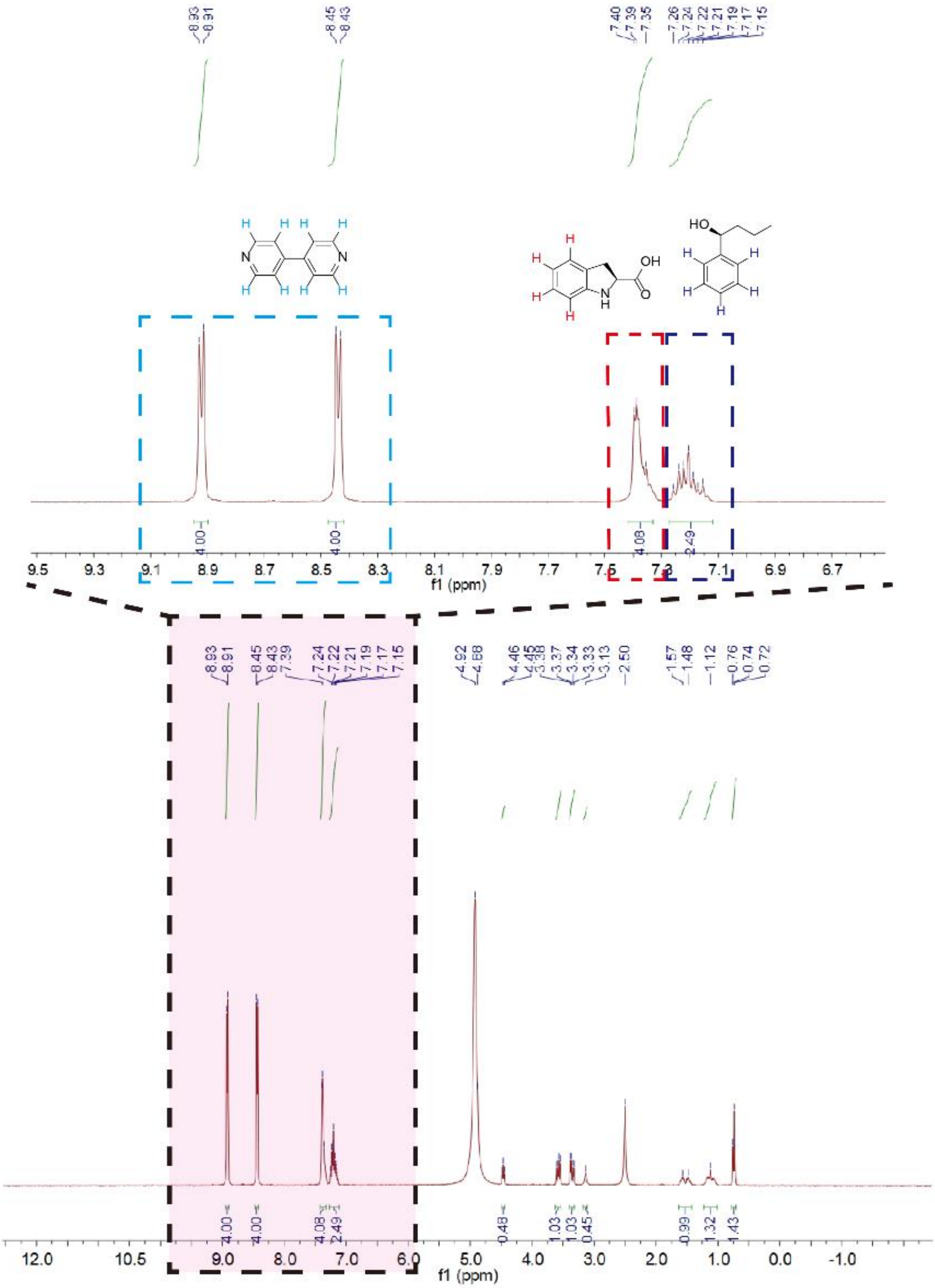

Figure S35. ^{1}H NMR spectrum of digested **CMOM-5-*S*-1P1B**. The ratio among bipy: *S*-IDEC: *S*-1P1B was solved to be 1:1.02:0.5.

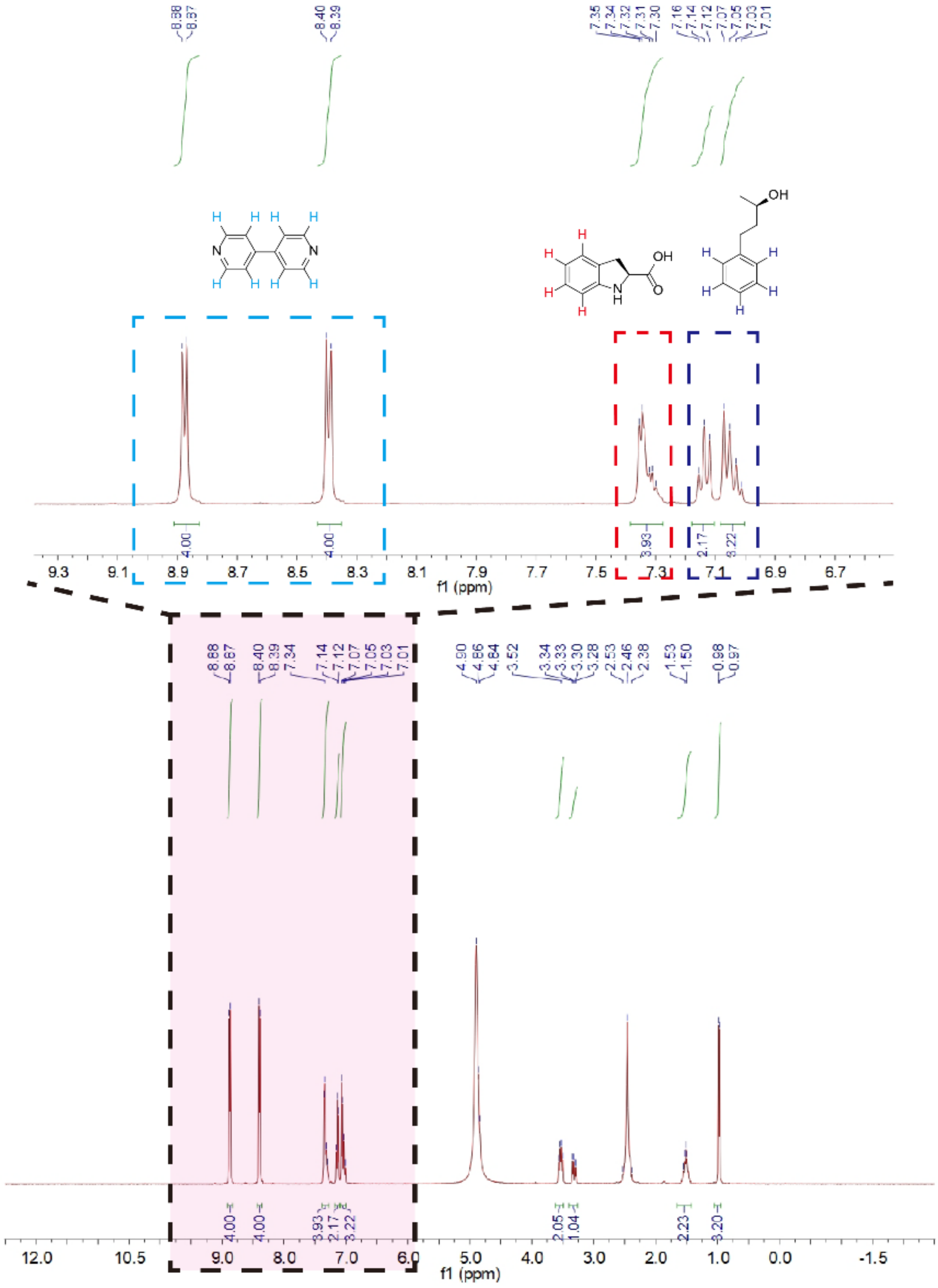

Figure S36. ^{1}H NMR spectrum of digested **CMOM-5-*R*-4P2B**. The ratio among bipy: *S*-IDEC: *R*-4P2B was solved to be 1:0.98:1.07.

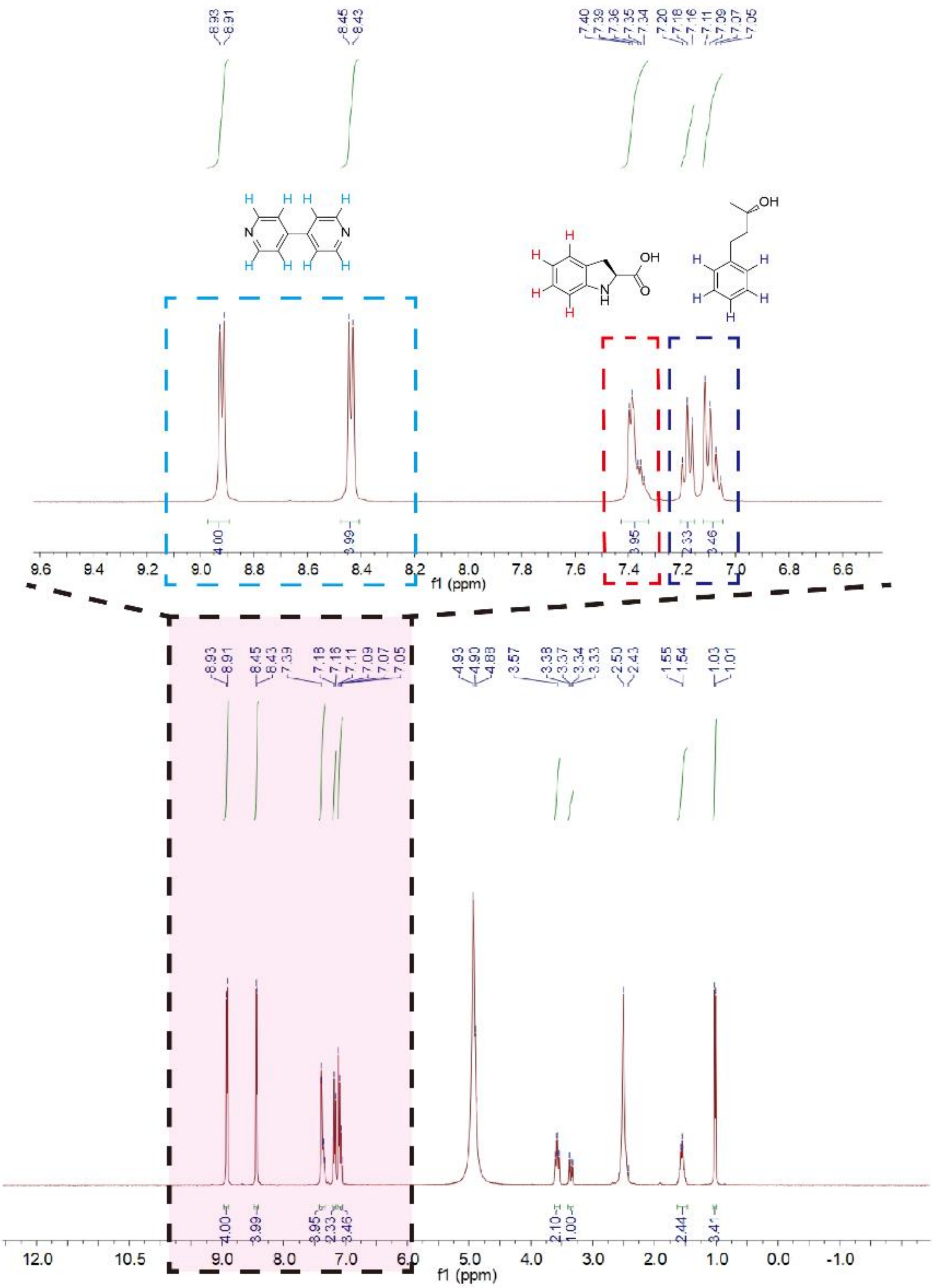

Figure S37. ^{1}H NMR spectrum of digested **CMOM-5-*S*-4P2B**. The ratio among bipy: *S*-IDEC: *S*-4P2B was solved to be 1:0.99:1.15.

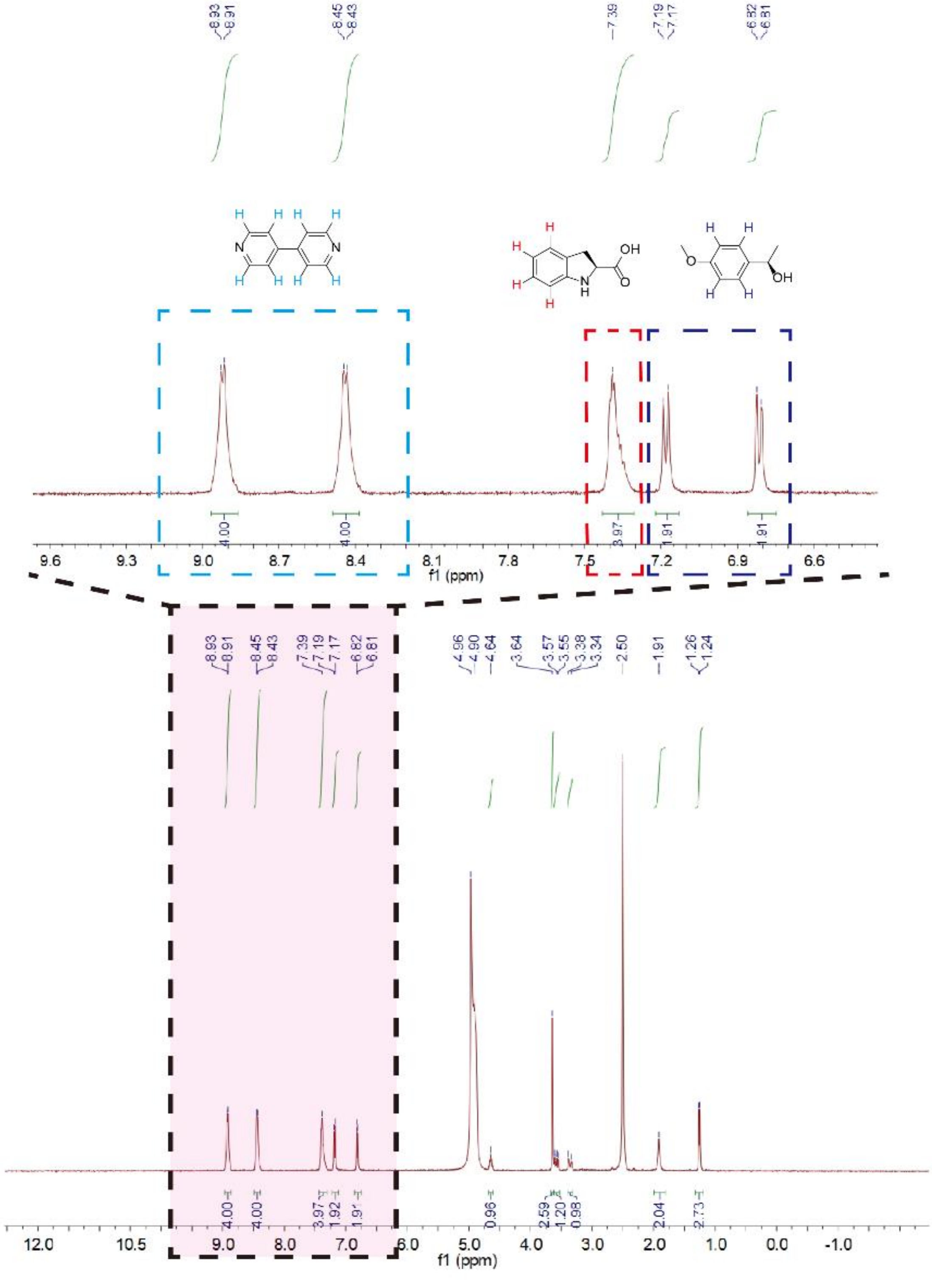

Figure S38. ^{1}H NMR spectrum of digested **CMOM-5-*R*-MPE**. The ratio among bipy: *S*-IDEC: *R*-MPE was solved to be 1:0.99:0.96.

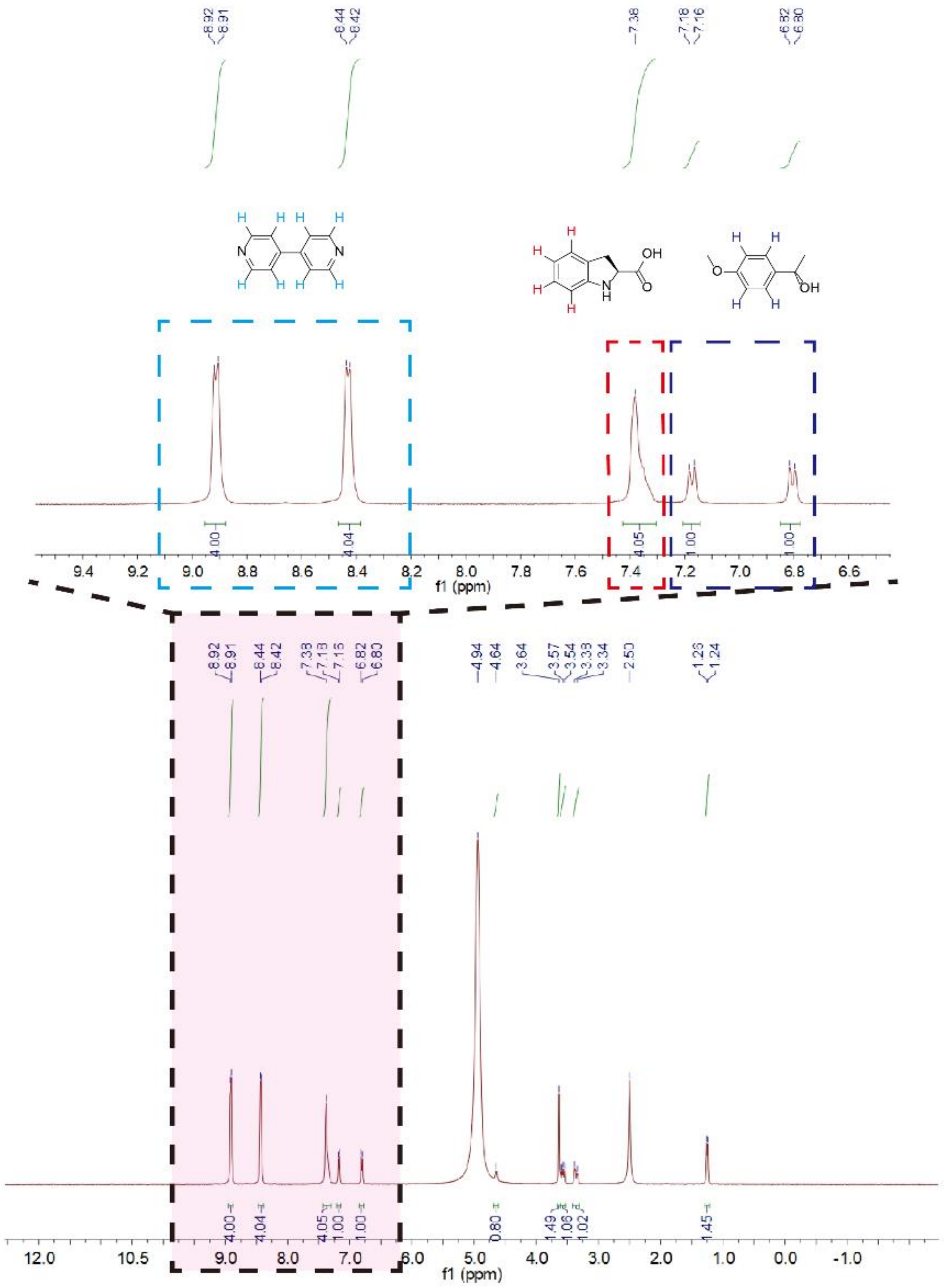

Figure S39. ^{1}H NMR spectrum of digested **CMOM-5-*S*-MPE**. The ratio among bipy: *S*-IDEC: *S*-MPE was solved to be 1:1.01:0.5.

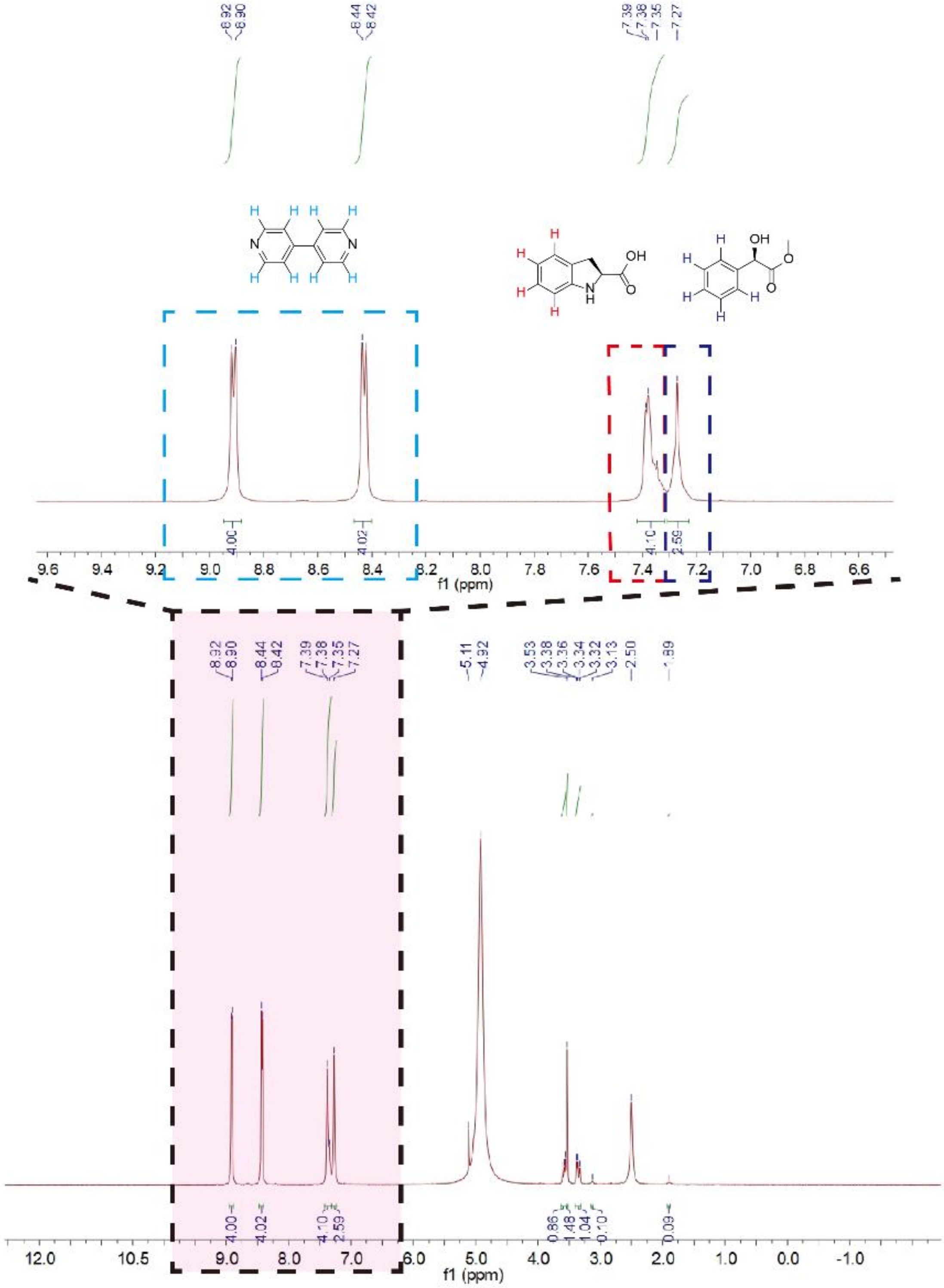

Figure S40. ^{1}H NMR spectrum of digested **CMOM-5-*R*-MM**. The ratio among bipy: *S*-IDEC: *R*-MM was solved to be 1:1.03:0.52.

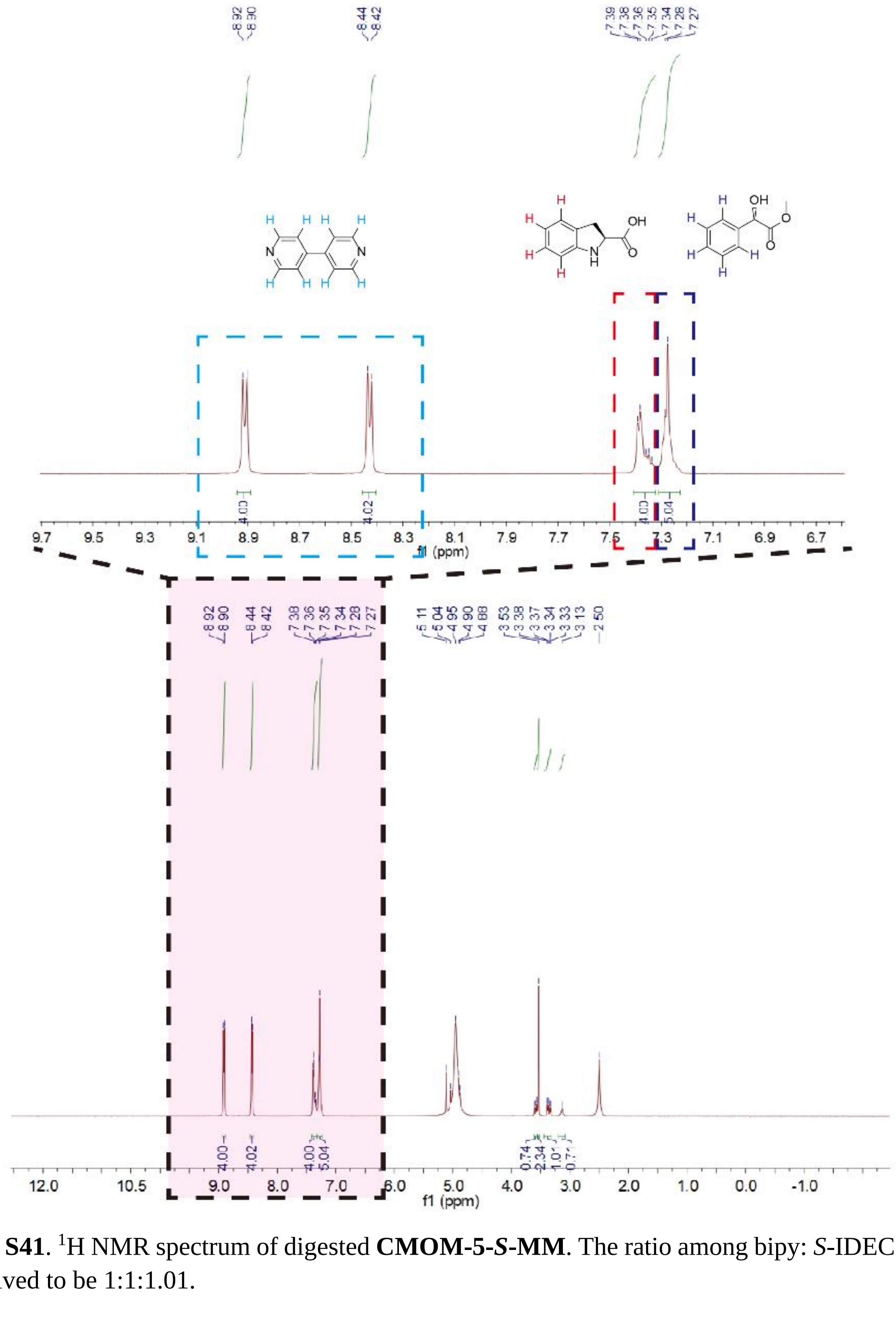

Figure S41. ^{1}H NMR spectrum of digested **CMOM-5-*S*-MM**. The ratio among bipy: *S*-IDEC: *S*-MM was solved to be 1:1:1.01.

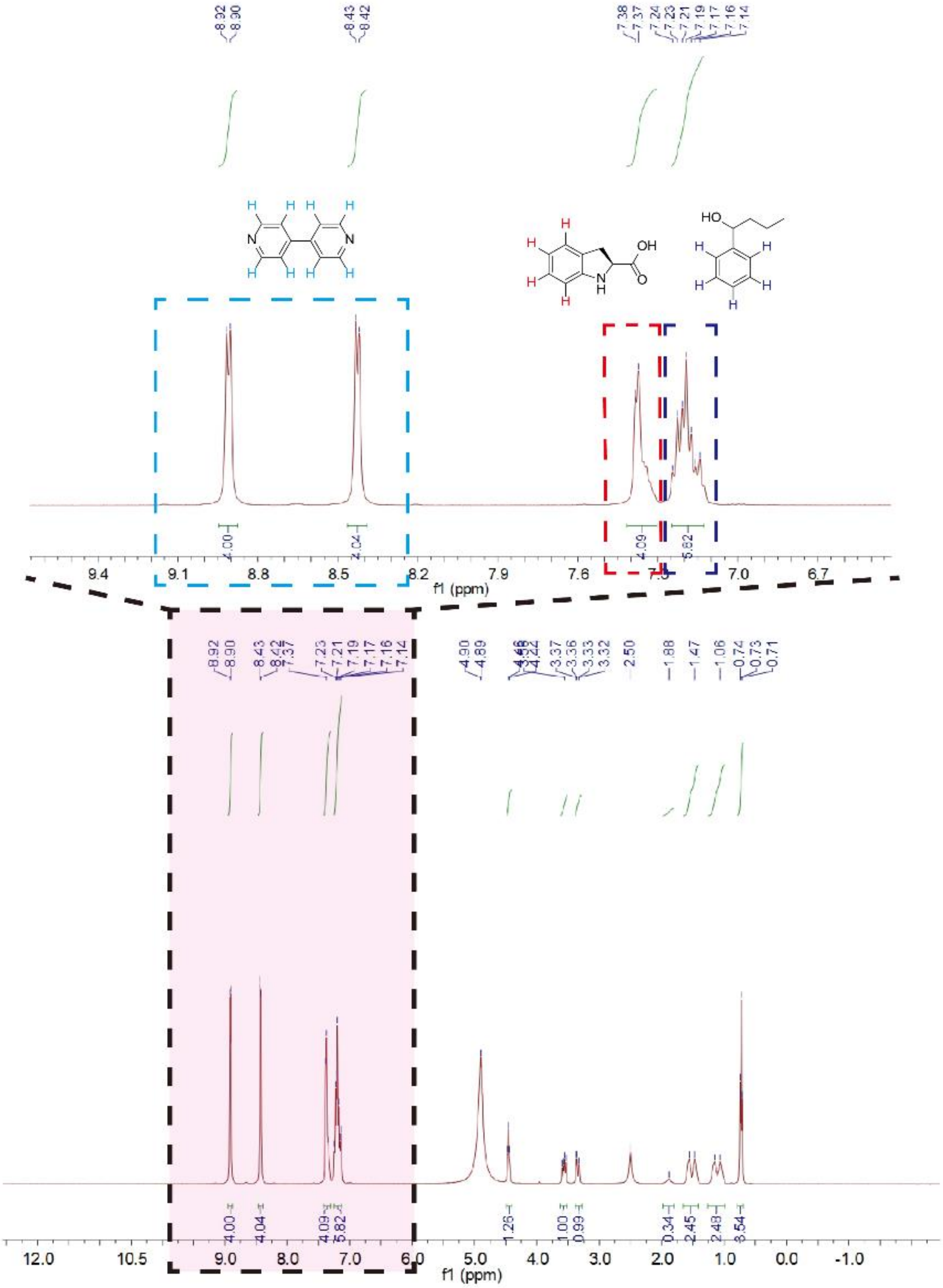

Figure S42. ^{1}H NMR spectrum of the digested **CMOM-5** collected from 1P1B chiral resolution experiment. The ratio among bipy: *S*-IDEC: 1P1B was solved to be 1:1.02:1.16.

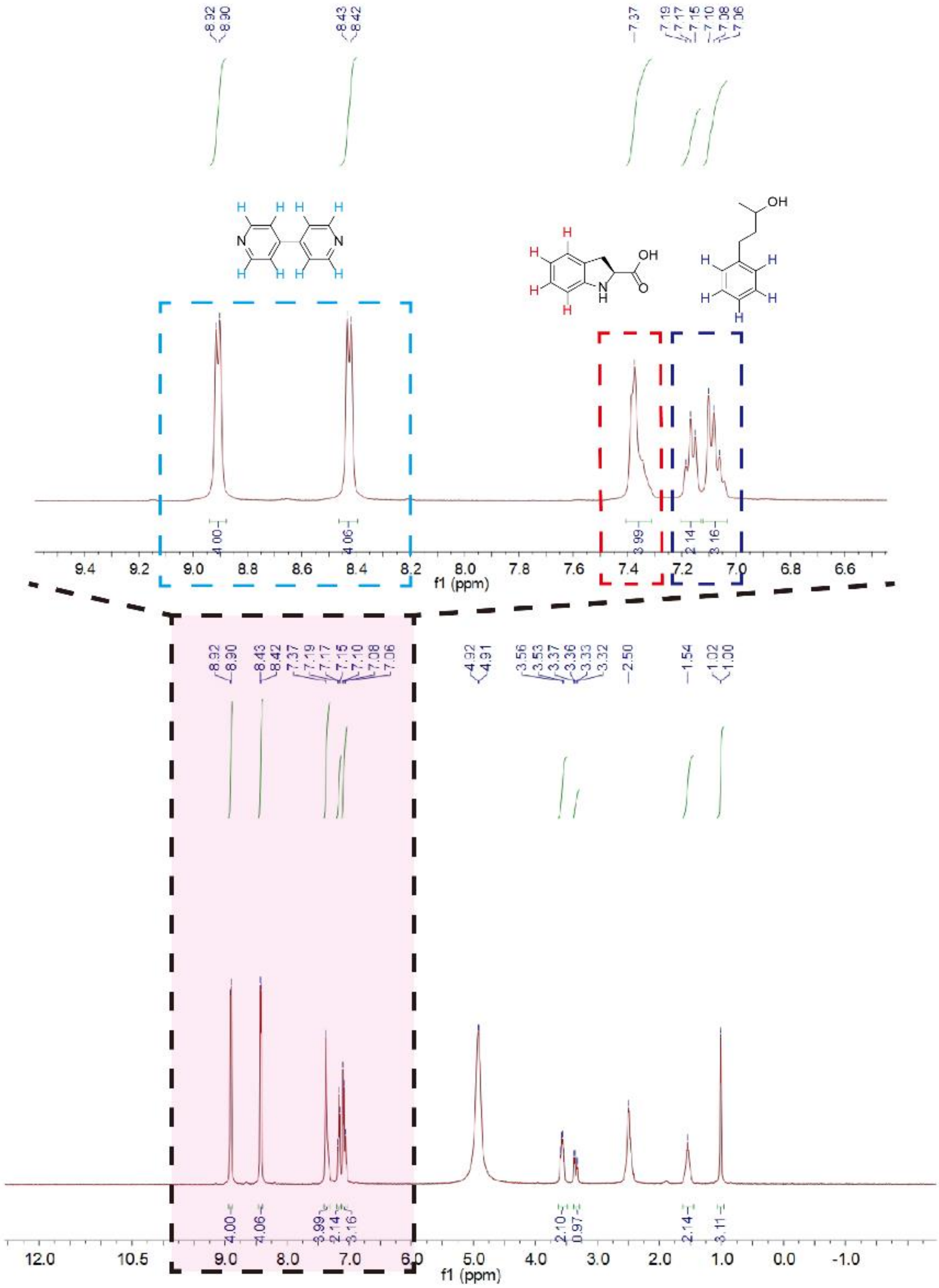

Figure S43. ^{1}H NMR spectrum of the digested **CMOM-5** collected from 4P2B chiral resolution experiment. The ratio among bipy: *S*-IDEC: 4P2B was solved to be 1:1:1.05.

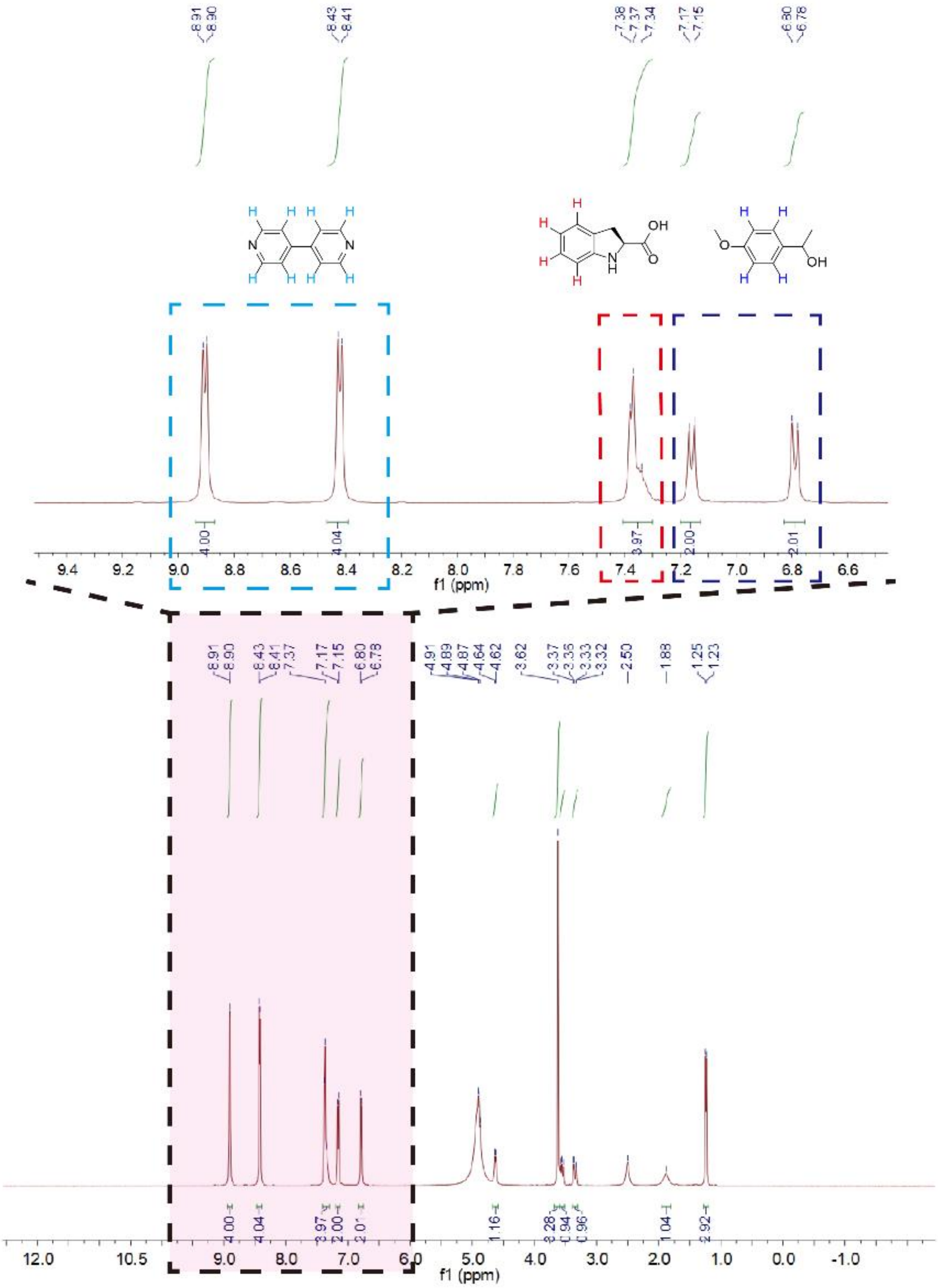

Figure S44. ^{1}H NMR spectrum of the digested **CMOM-5** collected from MPE chiral resolution experiment. The ratio among bipy: *S*-IDEC: MPE was solved to be 1:0.99:1.

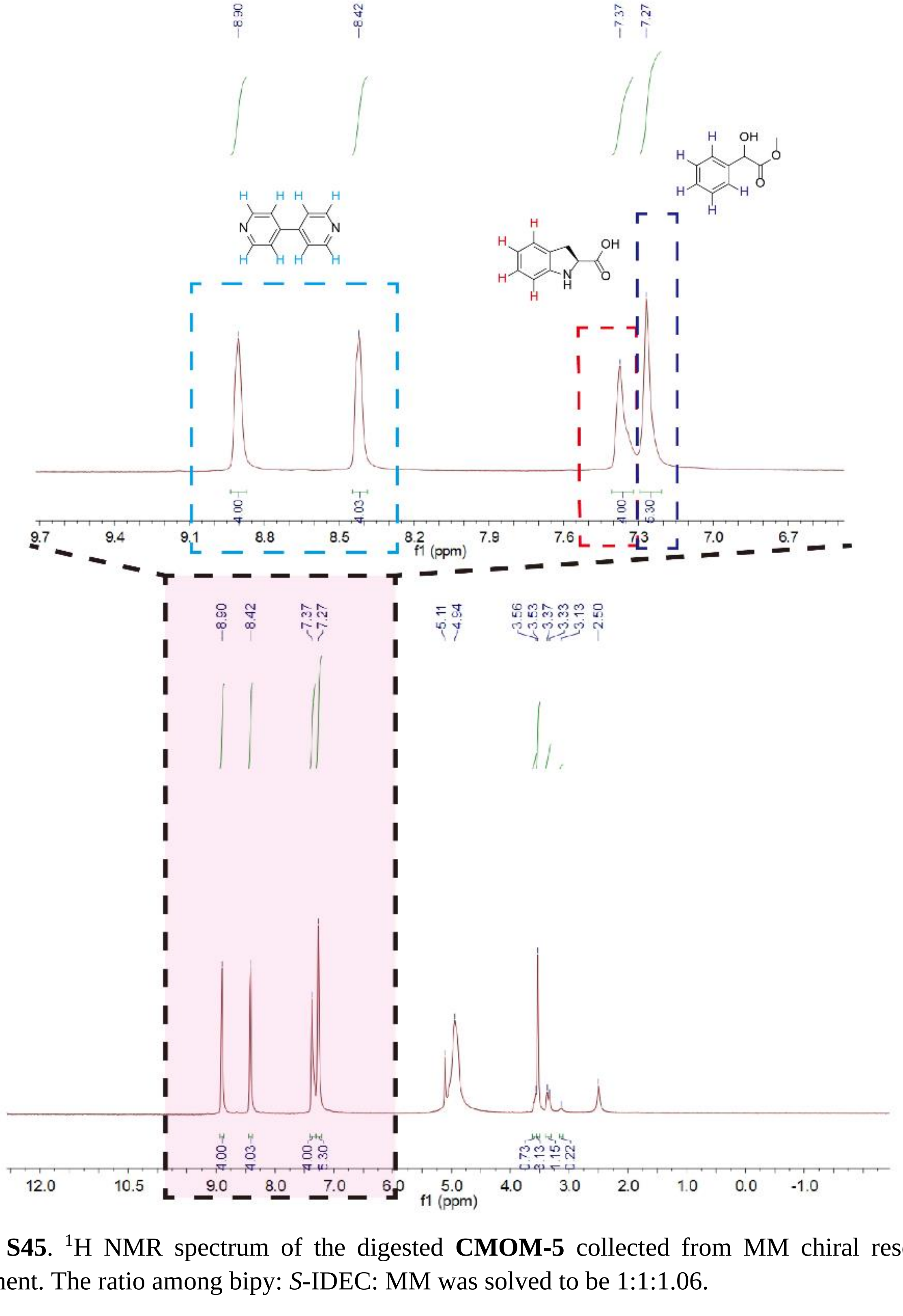

Figure S45. ^{1}H NMR spectrum of the digested **CMOM-5** collected from MM chiral resolution experiment. The ratio among bipy: *S*-IDEC: MM was solved to be 1:1:1.06.

HPLC Methods for the Analysis of Chiral Resolution Experiment Samples

High performance liquid chromatography (HPLC) characterization for the chiral resolution experiments was done by the use of a Shimadzu HPLC system. The HPLC system was consist of LC-20AT prominence liquid chromatograph, DGU-20A5R degassing unit, SIL-20A HT prominence auto sampler, SPD-20A prominence UV/Vis detector, and CTO-20AC prominence column oven. Specific methods for the analysis of each type of chiral molecules were list as the follows.

Samples of 1P1B were dissolved in methanol and analysed by the Daicel CHIRALPAK IC column (4.6 mm × 250 mm ID) at 25 °C. The injection volume is 5 μL. The eluent was n-hexane and IPA (n-hexane/IPA = 98/2). The flow rate was set at 1.0 mL/min. The wavelength of UV detector was set at 215 nm.

Samples of 4P2B were dissolved in methanol and analysed by the Daicel CHIRALPAK IB column (4.6 mm × 250 mm ID) at 25 °C. The injection volume is 5 μL. The eluent was n-hexane and IPA (n-hexane/IPA = 9/1). The flow rate was set at 0.8 mL/min constantly. The wavelength of UV detector was set at 250 nm.

Samples of MPE were dissolved in methanol and analysed by the Daicel CHIRALPAK IB column (4.6 mm × 250 mm ID) at 25 °C. The injection volume is 5 μL. The eluent was n-hexane and IPA (n-hexane/IPA = 98/2). The flow rate was set at 0.8 mL/min. The wavelength of UV detector was set at 220 nm.

Samples of MM were dissolved in methanol and analysed by the Daicel CHIRALPAK IB column (4.6 mm × 250 mm ID) at 25 °C. The injection volume is 5 μL. The eluent was n-hexane and IPA (n-hexane/IPA = 9/1). The flow rate was set at 0.8 mL/min constantly. The wavelength of UV detector was set at 250 nm.

<Chromatogram>

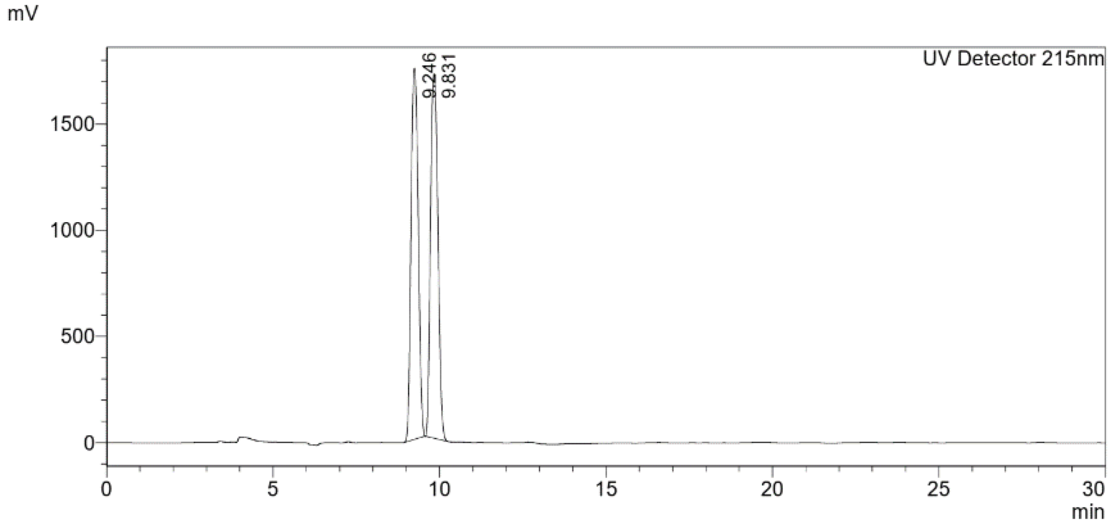

<Peak Table>

UV Detector 215nm

Peak#	Ret. Time	Area	Height	Conc.	Unit	Mark	Name
1	9.246	26207363	1746119	49.688		M	
2	9.831	26536811	1714779	50.312		M	
Total		52744174	3460898				

Figure S46. The HPLC chromatogram of racemic 1P1B.

<Chromatogram>

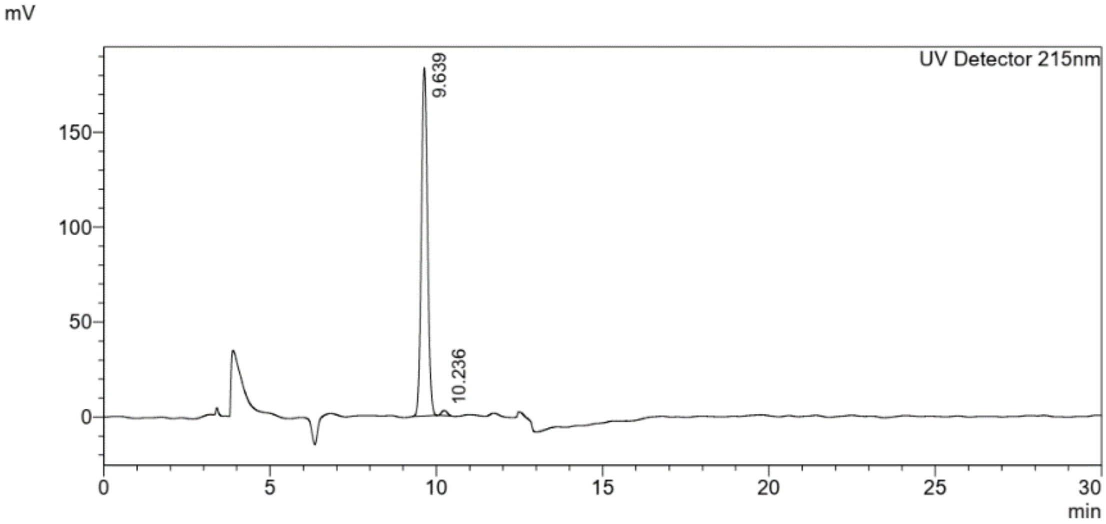

<Peak Table>

UV Detector 215nm

Peak#	Ret. Time	Area	Height	Conc.	Unit	Mark	Name
1	9.639	2339291	183200	98.714		M	
2	10.236	30475	2703	1.286		M	
Total		2369766	185902				

Figure S47. The HPLC chromatogram of *R*-1P1B.

<Chromatogram>

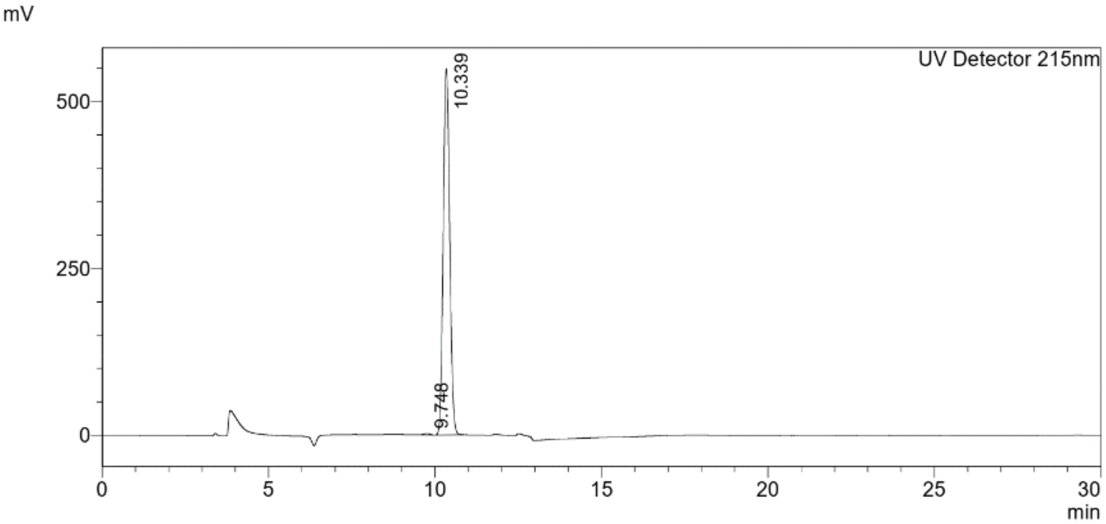

<Peak Table>

UV Detector 215nm

Peak#	Ret. Time	Area	Height	Conc.	Unit	Mark	Name
1	9.748	21125	1889	0.286		M	
2	10.339	7364962	547805	99.714		M	
Total		7386087	549695				

Figure S48. The HPLC chromatogram of *S*-1P1B.

<Chromatogram>

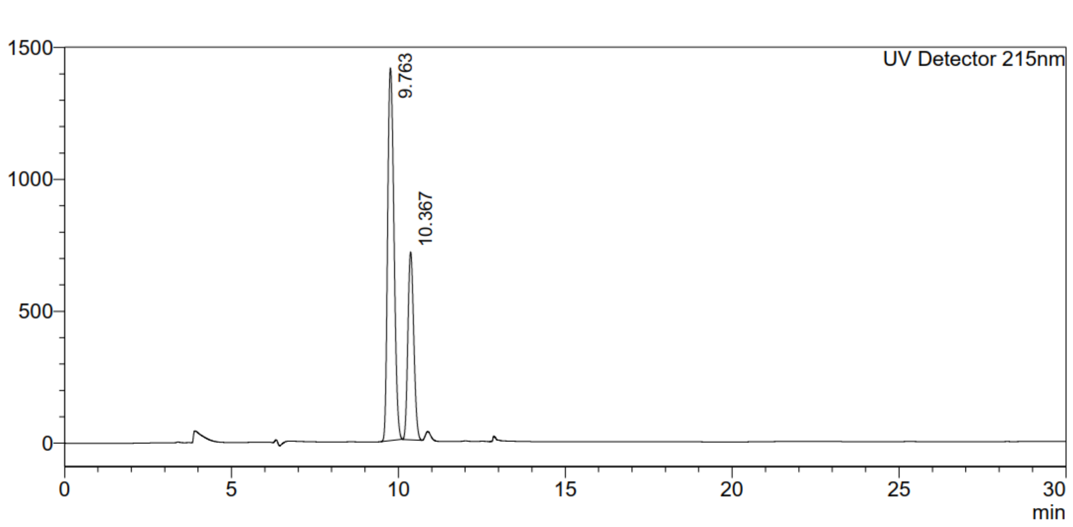

<Peak Table>

UV Detector 215nm

Peak#	Ret. Time	Area	Height	Conc.	Unit	Mark	Name
1	9.763	18594621	1410862	68.118		M	
2	10.367	8703138	709708	31.882		M	
Total		27297759	2120570				

Figure S49. The HPLC chromatogram of the solution extracted from 1P1B encapsulated **CMOM-5**.

<Chromatogram>

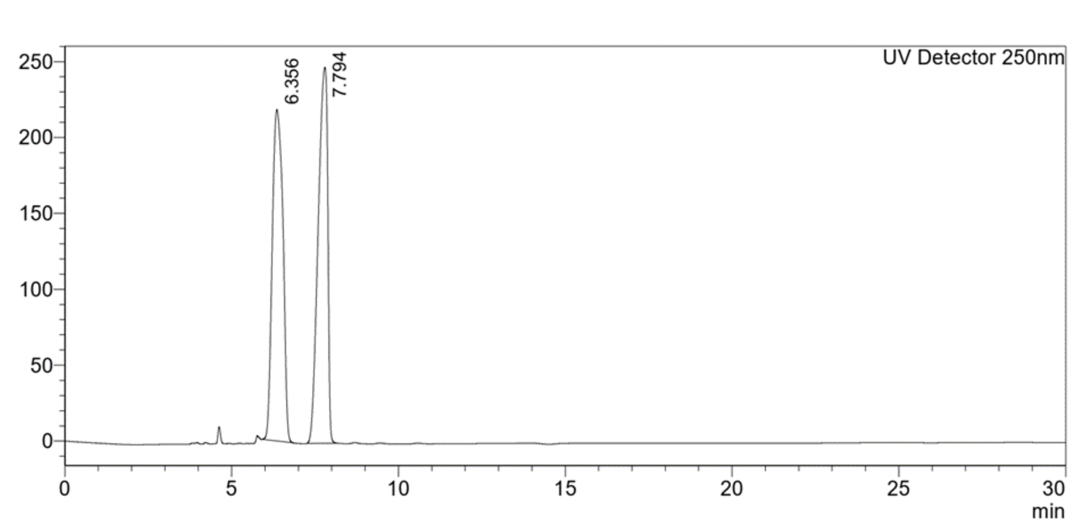

<Peak Table>

UV Detector 250nm

Peak#	Ret. Time	Area	Height	Conc.	Unit	Mark	Name
1	6.356	4839846	218304	49.571		M	
2	7.794	4923672	247577	50.429		M	
Total		9763518	465881				

Figure S50. The HPLC chromatogram of racemic 4P2B.

<Chromatogram>

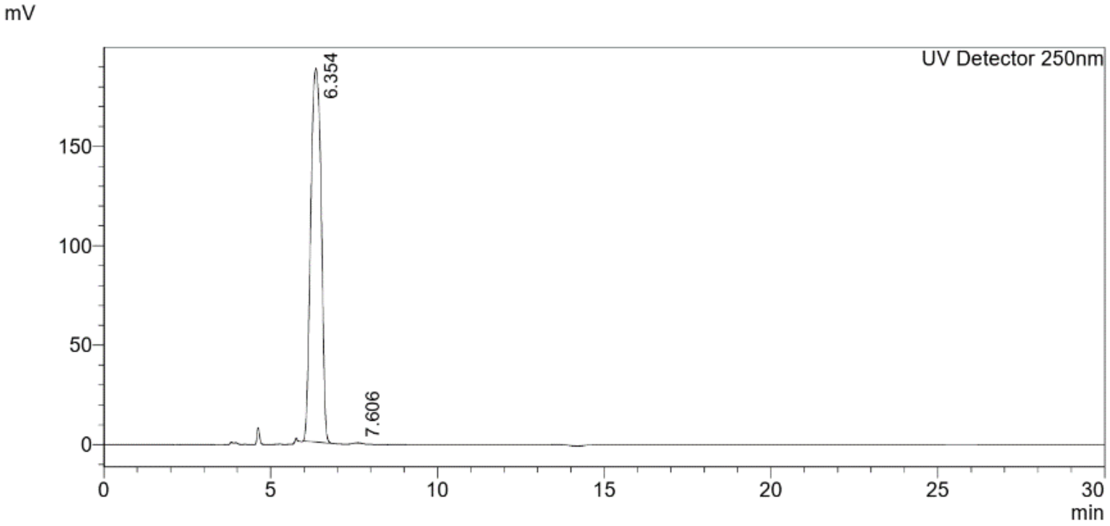

<Peak Table>

UV Detector 250nm

Peak#	Ret. Time	Area	Height	Conc.	Unit	Mark	Name
1	6.354	4075353	187859	99.932		M	
2	7.606	2775	253	0.068		M	
Total		4078128	188112				

Figure S51. The HPLC chromatogram of *R*-4P2B.

<Chromatogram>

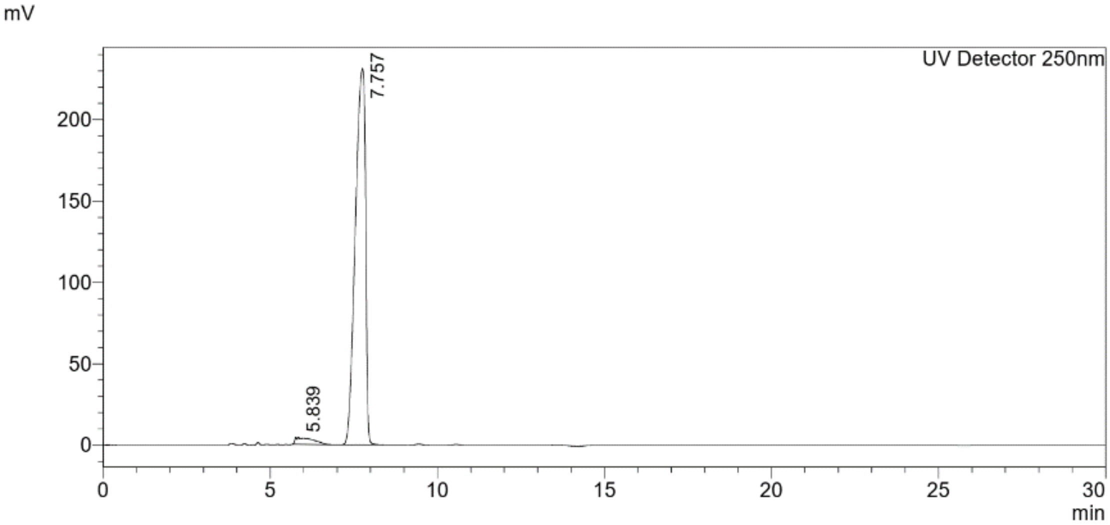

<Peak Table>

UV Detector 250nm

Peak#	Ret. Time	Area	Height	Conc.	Unit	Mark	Name
1	5.839	152159	4351	3.014		M	
2	7.757	4896675	231153	96.986		M	
Total		5048835	235504				

Figure S52. The HPLC chromatogram of *S*-4P2B.

<Chromatogram>

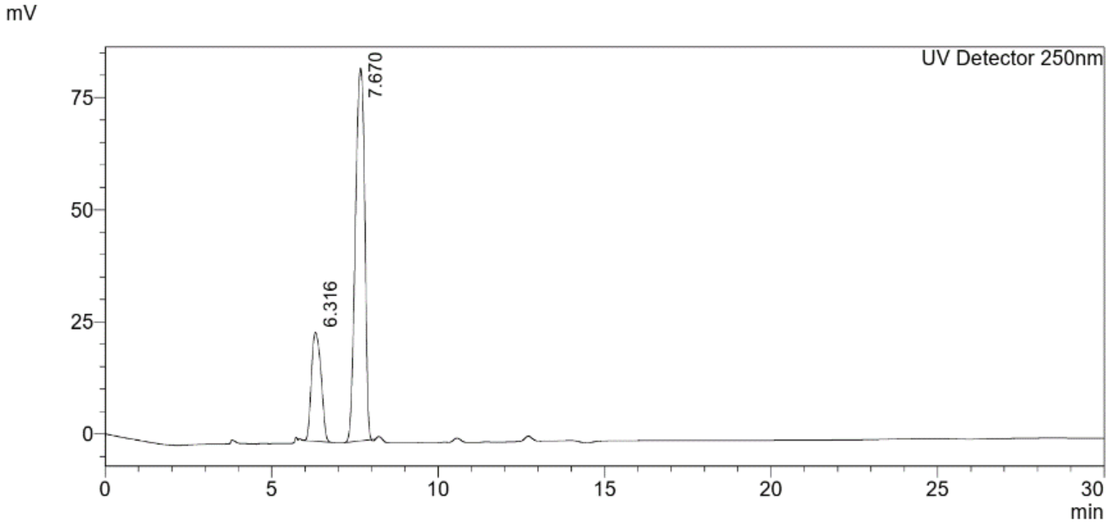

<Peak Table>

UV Detector 250nm

Peak#	Ret. Time	Area	Height	Conc.	Unit	Mark	Name
1	6.316	488101	24241	23.223		M	
2	7.670	1613701	83101	76.777		M	
Total		2101802	107342				

Figure S53. The HPLC chromatogram of the solution extracted from 4P2B encapsulated **CMOM-5**.

<Chromatogram>

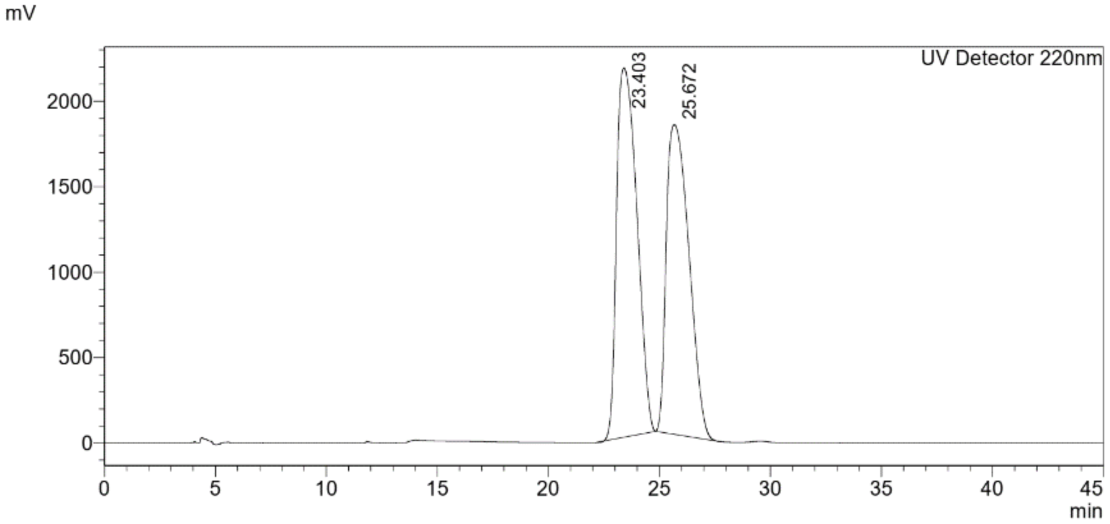

<Peak Table>

UV Detector 220nm

Peak#	Ret. Time	Area	Height	Conc.	Unit	Mark	Name
1	23.403	137291456	2160878	51.632		M	
2	25.672	128614078	1811989	48.368		M	
Total		265905535	3972867				

Figure S54. The HPLC chromatogram of racemic MPE.

<Chromatogram>

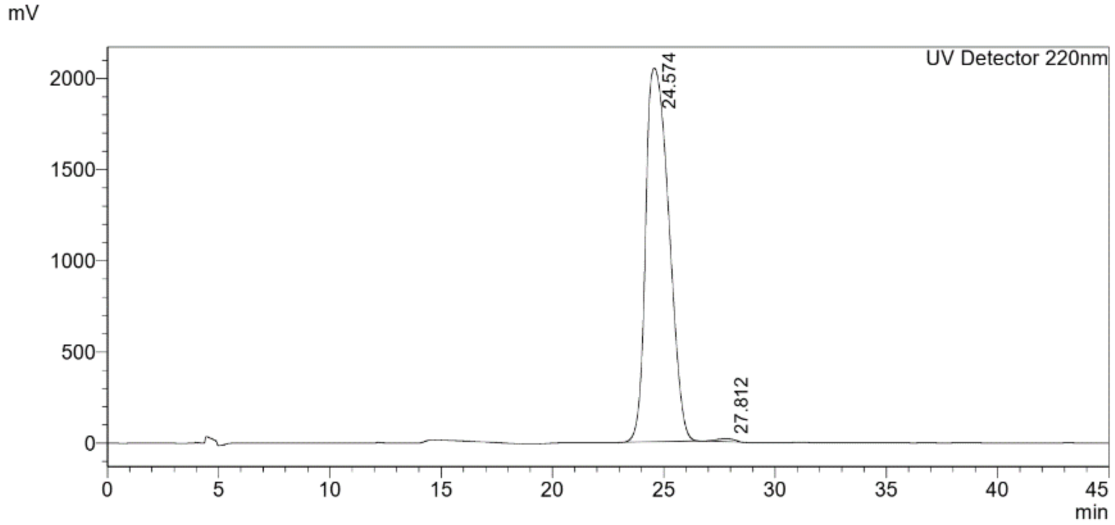

<Peak Table>

UV Detector 220nm

Peak#	Ret. Time	Area	Height	Conc.	Unit	Mark	Name
1	24.574	148677851	2048775	99.417		M	
2	27.812	871524	15155	0.583		M	
Total		149549376	2063931				

Figure S55. The HPLC chromatogram of *R*-MPE.

<Chromatogram>

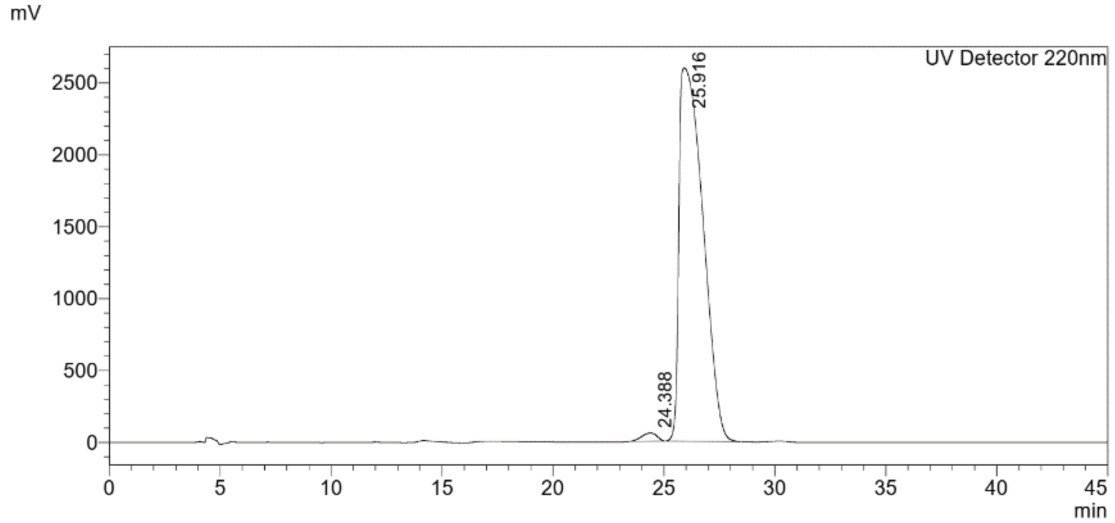

<Peak Table>

UV Detector 220nm

Peak#	Ret. Time	Area	Height	Conc.	Unit	Mark	Name
1	24.388	2615312	55623	1.329		M	
2	25.916	194212832	2596500	98.671		M	
Total		196828144	2652124				

Figure S56. The HPLC chromatogram of *S*-MPE.

<Chromatogram>

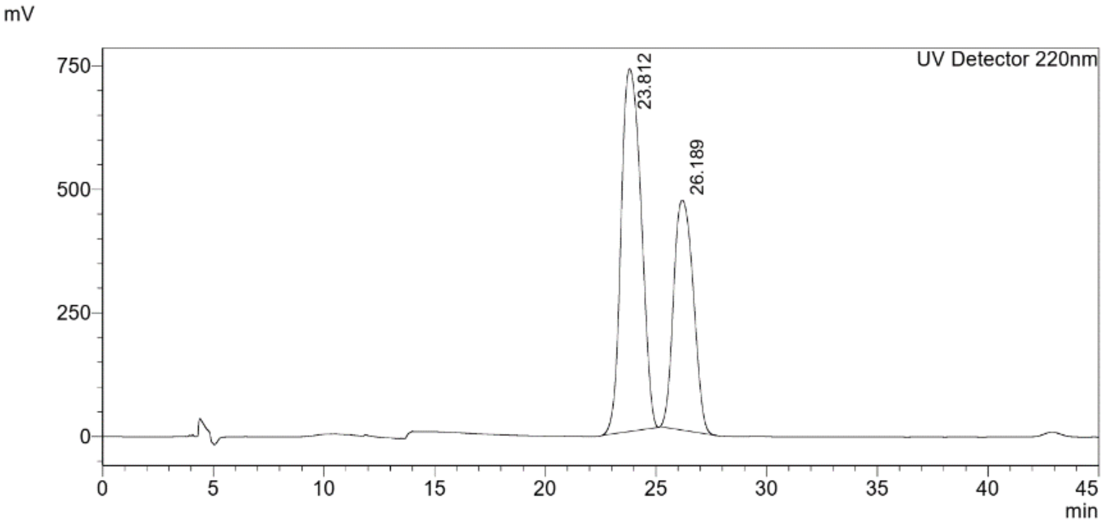

<Peak Table>

UV Detector 220nm

Peak#	Ret. Time	Area	Height	Conc.	Unit	Mark	Name
1	23.812	46034785	733647	61.456		M	
2	26.189	28872067	465223	38.544		M	
Total		74906851	1198869				

Figure S57. The HPLC chromatogram of the solution extracted from MPE encapsulated **CMOM-5**.

<Chromatogram>

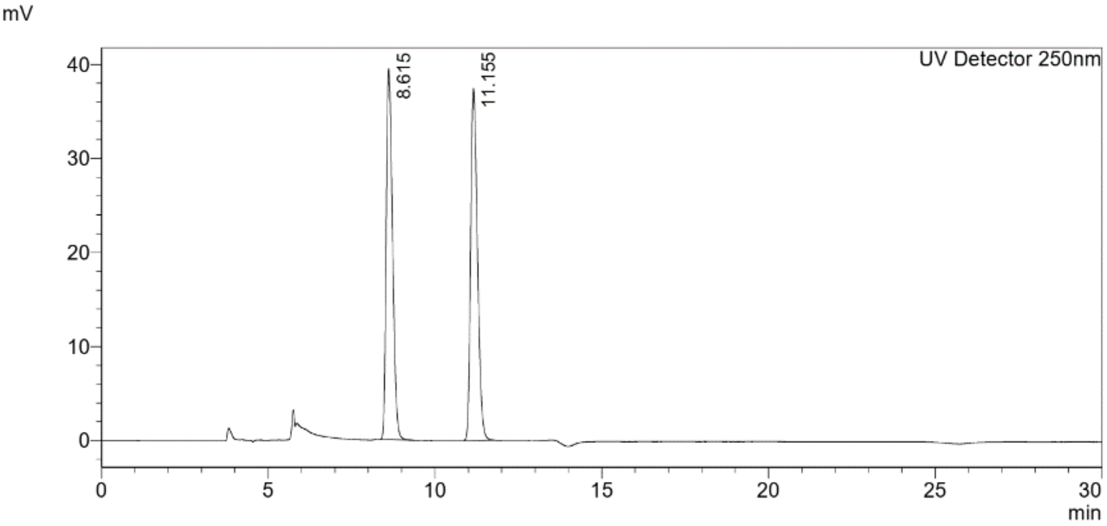

<Peak Table>

UV Detector 250nm

Peak#	Ret. Time	Area	Height	Conc.	Unit	Mark	Name
1	8.615	519774	39413	49.429		M	
2	11.155	531779	37439	50.571		M	
Total		1051553	76852				

Figure S58. The HPLC chromatogram of racemic MM.

<Chromatogram>

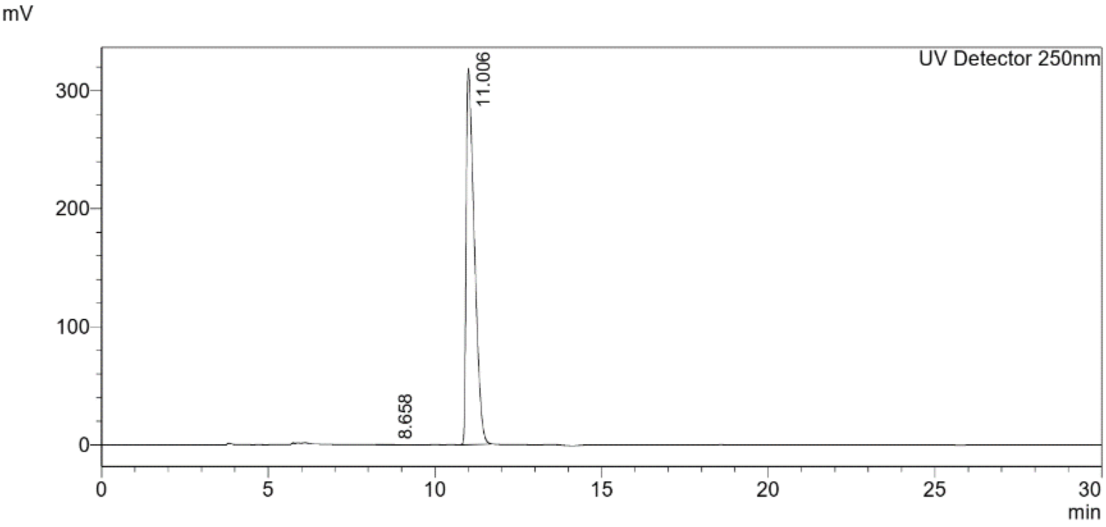

<Peak Table>

UV Detector 250nm

Peak#	Ret. Time	Area	Height	Conc.	Unit	Mark	Name
1	8.658	299	34	0.006		M	
2	11.006	5440668	318542	99.994		M	
Total		5440967	318576				

Figure S59. The HPLC chromatogram of *R*-MM.

<Chromatogram>

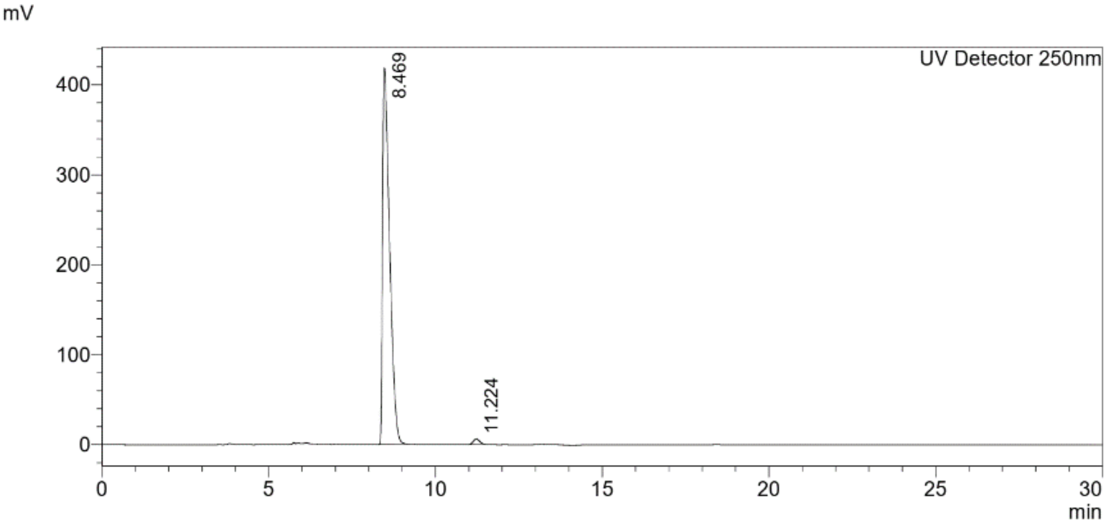

<Peak Table>

UV Detector 250nm

Peak#	Ret. Time	Area	Height	Conc.	Unit	Mark	Name
1	8.469	6125066	417843	98.772		M	
2	11.224	76162	5866	1.228		M	
Total		6201227	423710				

Figure S60. The HPLC chromatogram of *S*-MM.

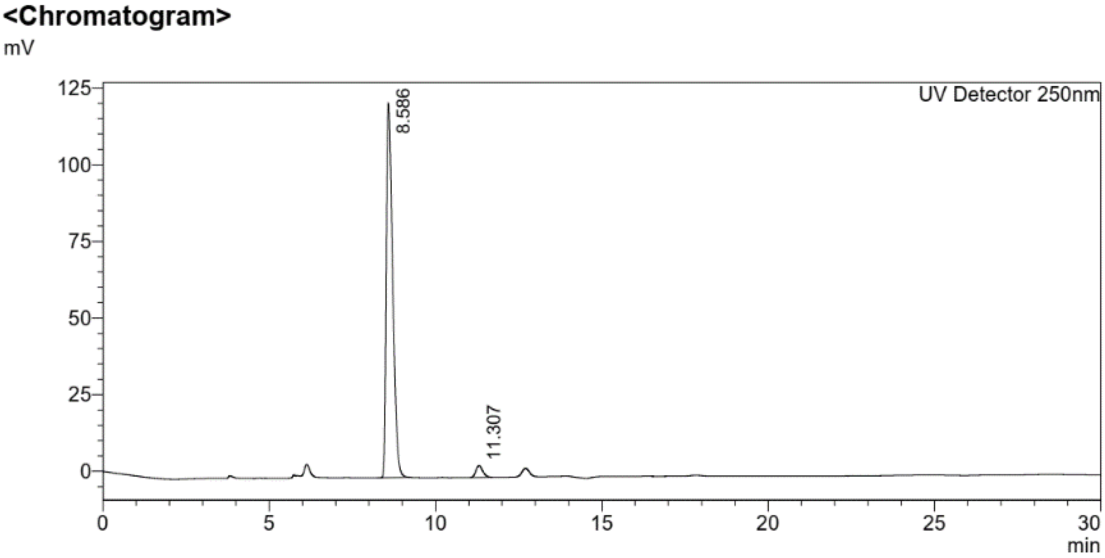

<Peak Table>

UV Detector 250nm

Peak#	Ret. Time	Area	Height	Conc.	Unit	Mark	Name
1	8.586	1640197	121947	96.763		M	
2	11.307	54876	3805	3.237		M	
Total		1695073	125752				

Figure S61. The HPLC chromatogram of the solution extracted from MM encapsulated **CMOM-5**.

Table S27. Chiral resolution performances of CMOM-5 *versus* the other relevant CMOM pair.

Materials	Ligands	*ee*, %				Reference
		1P1B	4P2B	MPE	MM	
**CMOM-5**	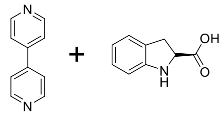	36.2[a]	53.6[b]	22.9[a]	93.5[b]	This work
**Δ_{12}-PCC-57**	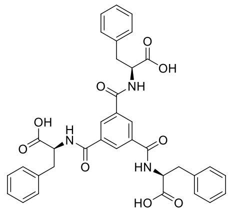	NM	99.9[b]	99.9[b]	NM	34
**$[DyNaL(H_2O)_4]_6(H_2O)$**	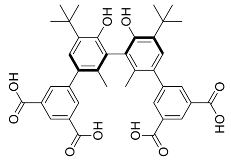	NM	NM	NM	93.1[b]	35

[a] *R* > *S*; [b] *S* > *R*; NM: not mentioned.

References

1. Ramadhar, T. R.; Zheng, S.-L.; Chen, Y.-S.; Clardy, J. Analysis of Rapidly Synthesized Guest-Filled Porous Complexes with Synchrotron Radiation: Practical Guidelines for the Crystalline Sponge Method. *Acta Cryst.* **2015**, *A 71*, 46-58. DOI: 10.1107/S2053273314019573.
2. Groom, C. R.; Bruno, I. J.; Lightfoot, M. P.; Ward, S. C. The Cambridge Structural Database. *Acta Cryst.* **2016,** *B 72*, 171-179. DOI: 10.1107/S2052520616003954.
3. Matsia, S.; Tsave, O.; Hatzidimitriou, A.; Gabriel, C.; Salifoglou, A. The Aqueous Structural Speciation of Binary Thallium-Hydroxycarboxylic Acid Systems. Structure-Chemical (Bio)Reactivity Correlations. *J. Inorg. Biochem.* **2021**, *222*, 111469. DOI: 10.1016/j.jinorgbio.2021.111469.
4. Zhang, G.; Wang, B.; Wei, Y.; Zhang, Q.; Cai, K.; Zhang, X.; Sun, Z.; Shi, Y. A Pair of Nonporous Homochiral Cobalt-Based Coordination Polymers for Enantioselective Recognition And Electrocatalysis. *Inorg. Chem. Commun.* **2016**, *69*, 70-74. DOI: 10.1016/j.inoche.2016.04.034.
5. Moriya, M.; Tominaga, S.; Hashimoto, T.; Tanifuji, K.; Matsumoto, T.; Ohki, Y.; Tatsumi, K.; Kaneshiro, J.; Uesu, Y.; Sakamoto, W.; Yogo, T. Non-Centrosymmetric Coordination Polymer with a Highly Hindered Octahedral Copper Center Bridged by Mandelate. *Inorg. Chem.* **2012**, *51* (8), 4689–4693. DOI: 10.1021/ic202669r.
6. Zhang, S. Y.; Yang, C. X.; Shi, W.; Yan, X. P.; Cheng, P.; Wojtas L.; Zaworotko M. J. A Chiral Metal-Organic Material that Enables Enantiomeric Identification and Purification. *Chem* **2017**, *3* (2), 281-289. DOI: 10.1016/j.chempr.2017.07.004.
7. Novitchi, G.; Pilet, G.; Luneau, D. 1D Co^{II} and Ni^{II} Chiral Polymers That Exhibit Ferromagnetic Interactions. *Euro. J. Inorg. Chem.* **2011**, *2011* (31), 4869–4877. DOI: 10.1002/ejic.201100534.
8. Guo, W.-Y.; Li, M.-L.; Shi, Y.-J.; Song, H.-H.; Yu, H.-T. Architectural Diversity of Six Coordination Compounds Based on (S)-(+)-Mandelic Acid And Different N-Donor Auxiliary Ligands: Syntheses, Structures, And Luminescent Properties. *J. Coord. Chem.* **2015**, 68, 4224-4241. DOI: 10.1080/00958972.2015.1100300.
9. Zhang, S.-Y.; Wojtas, L.; Zaworotko, M. J. Structural Insight into Guest Binding Sites in a Porous Homochiral Metal–organic Material. *J. Am. Chem. Soc.* **2015**, *137* (37), 12045–12049. DOI: 10.1021/jacs.5b06760.
10. Li, X.; Feng, Q.; Liu, M.; Deng, Q.; Jia, Z.; Sun, Y.; Zuo, X.; Shi, J.; Song, H.-H.; Yu, H.-T. D/L-Mandelic Acid-Based Three New Pb(II) Coordination Polymers: Influence of Temperature And 5,5′-Dimethyl-2,2′-Dipyridyl Ancillary Ligand on Constructuring Enantiomeric Coordination Polymers. *Inorg. Chem. Commun.* **2020**, *112*, 107730. DOI: 10.1016/j.inoche.2019.107730.
11. Tay, H. M.; Hua, C. A Structural Study into Halogenated Derivatives of Mandelic Acid as Building Blocks of Chiral Coordination Polymers. *J. Coord. Chem.* **2022**, *75* (11-14), 1656-1669. DOI: 10.1080/00958972.2022.2053958.
12. Taniguchi, Y.; Miwa, M.; Kitada, N. Crystalline Sponge X-ray Analysis Coupled with Supercritical Fluid Chromatography: A Novel Analytical Platform for the Rapid Separation, Isolation, and Characterization of Analytes. *Analyst* **2021**, *146* (17), 5230–5235. DOI: 10.1039/d1an00948f.
13. Wu, J.; Li, B.; Wang, H.; Lai, Y.; Ye, Y.; Zou, Y.; Tian, J.; Xu, Y. How the Magnetic Field Impacts the Chiroptical Activities of Helical Copper Enantiomers. *New J. Chem.* **2021**, *45* (42), 20021-20027. DOI: 10.1039/D1NJ03803F
14. Shiroo, I.; Yasuhiko, Y.; Yoshie, I.; Toshio, T. Structure of Aquachloro(4-hydroxy-L-prolinato)copper(II). *Bull. Chem. Soc. Jpn.* **1987**, *60* (3), 899-902. DOI: 10.1246/bcsj.60.899
15. Mikhalyova, E. A.; Kolotilov, S. V.; Cador, O.; Pointillart, F.; Golhen, S.; Ouahab, L.; Pavlishchuk,

V. V. Magnetic Properties and Circular Dichroism of 1D Chains Built from Chiral Mononuclear and Non-Chiral Trinuclear Cu(II) Complexes with α-Aminocarboxylates. *Inorg. Chim. Acta*. **2010**, *363* (13), 3453-3460. DOI: 10.1016/j.ica.2010.06.052

16. Ng, C.-H.; Fun, H.-K.; Teo, S.-B.; Teoh, S.-G.; Chinnnakali, K. Bis(L-Prolinato-N,O)zinc(II). *Acta Cryst.* **1995**, *C* 51, 244-245. DOI: 10.1107/S0108270194009340
17. Abendrot, M.; Chęcińska, L.; Kusz, J.; Lisowska, K.; Zawadzka, K.; Felczak, A.; Kalinowska-Lis, U. Zinc(II) Complexes with Amino Acids for Potential Use in Dermatology: Synthesis, Crystal Structures, and Antibacterial Activity. *Molecules*, **2020**, *25* (4), 951. DOI: 10.3390/molecules25040951
18. Karpagam, S.; Kartikeyan, R.; Nachiyar, P. P.; Velusamy, M.; Kannan, M.; Krishnan, M.; Chitgupi, U.; Lovell, J. F.; Akbarsha, M. A.; Rajendiran, V. ROS-mediated Cell Death Induced by Mixed Ligand Copper(II) Complexes of L-Proline and Diimine: Effect of Co-Ligand. *J. Coord. Chem.* **2019**, *72* (18), 3102-3127. DOI: 10.1080/00958972.2019.1680834
19. Estrader, M.; Diaz, C.; Ribas, J.; Solans, X.; Font-Bardía, M. Synthesis, Characterization and Magnetic Properties of Six New Copper(II) Complexes with Aminoacids as Bridging Ligand, Exhibiting Ferromagnetic Coupling. *Inorg. Chim. Acta* **2008**, *361* (14-15), 3963-3969. DOI: 10.1016/j.ica.2008.03.028
20. Ingleson, M. J.; Bacsa, J.; Rosseinsky, M. J. Homochiral H-Bonded Proline based Metal Organic Frameworks. *Chem. Commun.*, **2007**, (29) 3036-3038. DOI: 10.1039/B706557D
21. Yukawa, Y. Structures and Properties of (L-Prolinato)Copper(II) Complexes Prepared from Different Solvents. *J. Chem. Soc., Dalton Trans.*, **1992**, (22), 3217-3221. DOI: 10.1039/DT9920003217
22. Narvekar, K. U.; Srinivasan B. R. CCDC 2014925: Experimental Crystal Structure Determination, 2020, DOI: 10.5517/ccdc.csd.cc25mpl3
23. Narvekar, K. U.; Srinivasan B. R. CCDC 2039516: Experimental Crystal Structure Determination, 2020, DOI: 10.5517/ccdc.csd.cc26g8vt
24. Hu, T.; Liu, T.; Zhang, Z.; Wang, Y.; Yang, Y.; Young, D. J.; Hu, C.; Lang, J.-P. Precise Control of Chirality Transfer by Adjusting the Alkyl Substituents of Guests. *Dyes Pigm.* **2018**, *160*, 692-699. DOI: 10.1016/j.dyepig.2018.09.004.
25. Akazome, M.; Takahashi, T.; Ogura, K. Enantiomeric Inclusion of α-Hydroxy Esters by (*R*)-(1-Naphthyl)glycyl-(*R*)-phenylglycine and the Crystal Structures of the Inclusion Cavities. *J. Org. Chem.* **1999**, *64* (7), 2293-2300. DOI: 10.1021/jo9818778.
26. Mravik, A.; Böcskei, Z.; Katona, Z.; Markovits, I.; Pokol, G.; Menyhárd, D. K.; Fogassy, E. A New Optical Resolution Method: Coordinative Resolution of Mandelic Acid Esters. The Crystal Structure of Calcium Hydrogen (2*R*,3*R*)-O,O′-Dibenzoyl Tartrate–2(*R*)-(–)-Methyl Mandelate. *Chem. Commun.* **1996**, (16), 1983-1984. DOI: 10.1039/CC9960001983.
27. Albrecht, M.; Borba, A.; Barbu-Debus, K. L.; Dittrich, B.; Fausto, R.; Grimme, S.; Mahjoub, A.; Nedić, M.; Schmitt, U.; Schrader, L.; Suhm, M. A.; Zehnacker-Rentien, A.; Zischang, J. Chirality Influence on the Aggregation of Methyl Mandelate. *New J. Chem.* **2010**, *34* (7), 1266-1285. DOI: 10.1039/c0nj00142b.
28. Krause, L.; Herbst-Irmer, R.; Sheldrick, G. M.; Stalke, D. Comparison of Silver and Molybdenum Microfocus X-ray Sources for Single-Crystal Structure Determination. *J. Appl. Cryst.* **2015**, *48*, 3-10. DOI: 10.1107/S1600576714022985.

29. Sheldrick, G. M. SHELXT – Integrated space-group and crystalstructure determination. *Acta Cryst.* **2015**, *A* 71, 3-8. DOI: 10.1107/S2053273314026370
30. Sheldrick, G. M. Crystal structure refinement with SHELXL. *Acta Cryst.* **2015** *C* **71**, 3-8. DOI: 10.1107/S2053229614024218
31. Dolomanov, O. V.; Bourhis, L. J.; Gildea, R. J.; Howard, J. A. K.; Puschmann, H. OLEX2: a Complete Structure Solution, Refinement and Analysis Program. *J. Appl. Cryst.* **2009**, *42*, 339-341. DOI: 10.1107/S0021889808042726.
32. Spek A. L. PLATON SQUEEZE: A Tool for the Calculation of the Disordered Solvent Contribution to the Calculated Structure Factors. *Acta Cryst.* **2015**, *C 71*, 9-18. DOI: 10.1107/S2053229614024929
33. Fulmer, G. R.; Miller, A. J. M.; Sherden, N. H.; Gottlieb, H. E.; Nudelman, A.; Stoltz, B. M.; Bercaw, J. E.; Goldberg, K. I. NMR Chemical Shifts of Trace Impurities: Common Laboratory Solvents, Organics, and Gases in Deuterated Solvents Relevant to the Organometallic Chemist, *Organometallics*, **2010**, *29* (9), 2176-2179, DOI: 10.1021/om100106e.
34. Zhu, C.; Tang, H.; Yang, K.; Fang, Y.; Wang, K.-Y.; Xiao, Z.; Wu, X.; Li, Y.; Powell, J. A.; Zhou, H.-C. Homochiral Dodecanuclear Lanthanide "cage in Cage" for Enantioselective Separation. *J. Am. Chem. Soc.* **2021**, *143* (32), 12560–12566. DOI: 10.1021/jacs.1c03652.
35. Peng, Y.; Gong, T.; Cui, Y. A Homochiral Porous Metal–organic Framework for Enantioselective Adsorption of Mandelates and Photocyclizaton of Tropolone Ethers. *Chem. Commun.* **2013**, *49* (74), 8253. DOI: 10.1039/c3cc43549k.
